# Dual Agonist/Antagonist Modulation of α9-Containing Nicotinic Acetylcholine Receptors by 2‑Ammoniumethyl Ethers of Stilbenol and Stilbenol Analogues

**DOI:** 10.1021/acs.jmedchem.5c02173

**Published:** 2025-12-08

**Authors:** Alessandro Giraudo, Han-Shen Tae, Andrew Hung, Katrin Richter, Bhavana Shivankar, Edoardo Armano, Veronika Grau, Marco Pallavicini, David J. Adams, Cristiano Bolchi

**Affiliations:** † Department of Pharmaceutical Sciences, 9304 Università Degli Studi of Milan, Milano I-20133, Italy; ‡ Molecular Horizons, Faculty of Science, Medicine and Health, 8691 University of Wollongong, Wollongong, NSW 2522, Australia; § School of Science, STEM College, 683873 RMIT University, Melbourne, Victoria 3001, Australia; ∥ Department of General and Thoracic Surgery, Laboratory of Experimental Surgery, 9175 Justus-Liebig-University, German Center for Lung Research [DZL], Cardio-Pulmonary Institute [CPI], Giessen 35385, Germany; ⊥ Department of Natural Sciences, Bonn-Rhein-Sieg University of Applied Sciences, Rheinbach 53359, Germany; # Physical and Materials Chemistry Division, CSIR-National Chemical Laboratory, Pune 41108, India

## Abstract

2-(Cyclohexyldimethylammoniumethyl)­ether of 4-stilbenol (**2**), and its styryl-modified analogues **21** and **22**, were identified as lead compounds from a series targeting human α9α10, α9, and α7 nicotinic acetylcholine receptors (nAChRs). Compounds **2** and **21** exhibited potent, and subtype-selective modulation of α9-containing receptors, with low nanomolar IC_50_ values and dual agonist/antagonist activity in a concentration-dependent manner. In contrast, compound **22** acted as a selective, pure antagonist. Molecular dynamics (MD) simulations of compound **21** supported a concentration-dependent allosteric mechanism, with orthosteric binding at low concentrations and vestibular site interaction at higher levels. In a human monocytic cell line, all three compounds inhibited ATP-induced IL-1β release at nanomolar concentrations. These findings identify α9α10-selective ligands as promising scaffolds for the development of nonopioid analgesics and immunomodulators, with favorable selectivity over α7 nAChRs to minimize CNS-related side effects.

## Graphical abstract

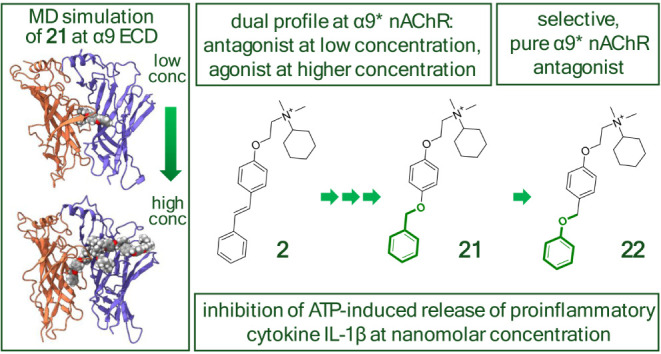

## Introduction

Mammalian nonmuscle nicotinic acetylcholine receptors (nAChRs) are ligand-gated ion channels composed of five protein subunits, selected from α2−α7, α9, α10, and β2−β4. These subunits assemble into distinct receptor subtypes, forming either heteromeric pentamers, containing all α subunits (e.g., α9α10) or at least one α and one β subunit (e.g., α3β2), or homomeric pentamers, composed entirely of α7 or α9 subunits. Notably, α9 subunits, when paired with α10 subunits, form heteropentameric nAChRs with two possible stoichiometries, (α9)_2_(α10)_3_ and (α9)_3_(α10)_2_, both exhibiting pharmacological profiles similar to homomeric α9 nAChRs.^1,2^

Nonmuscle nAChRs play a crucial role in diverse physiological processes. Over the past few decades, extensive research has focused on the ionotropic modulation of nAChR subtypes highly expressed in the brain, particularly heteromeric α4β2 and homomeric α7 nAChRs, as potential treatment for neurological disorders.^3–10^ However, despite significant efforts, few drugs targeting these receptors have reached clinical approval.^11,12^ More recently, α7 and α9* receptors (where * denotes the possible presence of α10 subunits) have gained attention as noncanonical nicotinic receptors (NCNRs).^13,14^ These receptors are expressed in various non-neuronal cells, particularly immune cells, where their function, linked to the cholinergic anti-inflammatory system (CAS), appears to involve metabotropic rather than ionotropic signaling.^14,15^ This renewed interest in α7 and α9* nAChRs as druggable targets has opened new avenues for pain and inflammation therapy, highlighting the need for selective ligands that effectively target these receptors with minimized off-target effects. The potential metabotropic function of these receptors further underscores the importance of developing and characterizing candidate ligands. Subtype selectivity of these ligands and expression pattern of the selected receptor subtype as limited as possible to the targeted tissues are key conditions to avoid off-target effects.

Compared to α4β2 and α7 nAChRs, α9* nAChRs offer a distinct advantage due to their more restricted expression, which excludes the central nervous system (CNS) and is confined to specific peripheral tissues, including immune cells.^16^ Therefore, targeting α9* nAChRs is a promising strategy for pain and inflammation modulation, potentially avoiding the failures that hindered α4β2 ligands as analgesics and that similarly threatened the development of α7 ligands for these indications.^17–19^

The composition and stoichiometry of the nAChRs involved in immune cell modulation are complex, incorporating α7, α9, and/or α10 subunits.^14,20^ While α7 forms homopentamers, α9 and α10 subunits can assemble into either homopentamers or heteropentamers with varying α9/α10 ratios.^16^ Given this complexity, the rational development of ligands with anti-inflammatory and analgesic activity requires thorough pharmacological characterization, beginning with electrophysiological analysis of their effects on α7 and α9* receptors. Previously, we reported that specific modifications of MG624 (**1**, 2-triethylammonium ethyl ether of 4-stilbenol) (Chart [Fig chart1]), an α7 and α9* nAChR antagonist with high α7-nAChR affinity (*K*i = 104 nM) and partial agonist activity at high supra-micromolar concentrations, shifted its selectivity toward α9* nAChRs.^21^ In particular, compounds **2**, **3**, and **4** (Chart [Fig chart1]), featuring a significantly enlarged ammonium head (**2**), a rigidified O–N linker within a six-membered nitrogen heterocycle (**3**), or a saturated vinylene bridge (**4**), exhibited α9* antagonism with reduced affinity and loss of inhibitory activity at the homopentameric α7 nAChR compared to **1**. At high concentrations (10–100 μM), these compounds also displayed intrinsic agonist activity at α9* nAChRs and significantly less to no agonist activity at α7 nAChRs.

**1 chart1:**
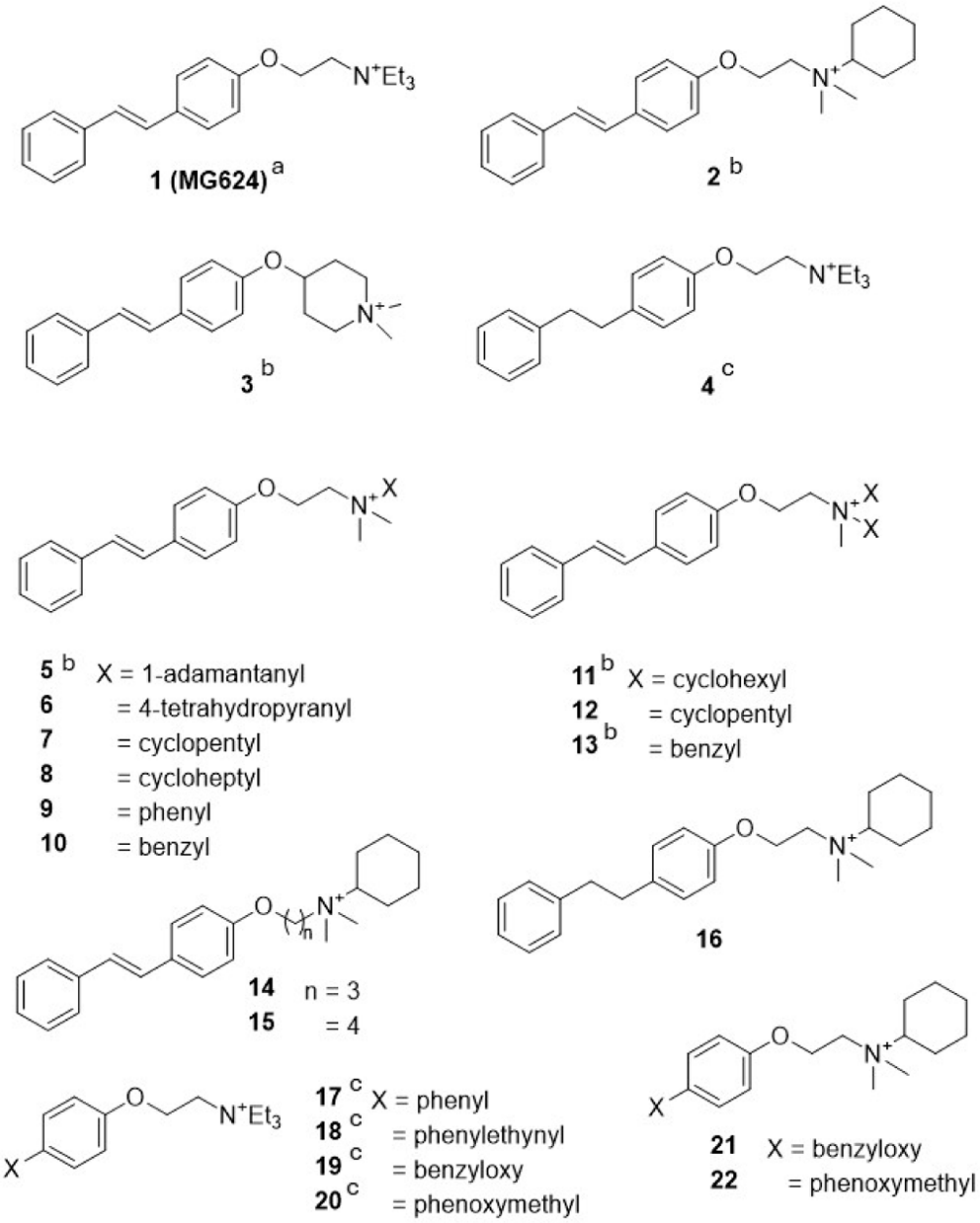
Compounds **1–4** and Analogues Modified at the Ammonium Head (**5–13**), at the O–N Linker (**14, 15**), and by Replacing the Styryl Moiety (**16–22**)

a Ref 21 and 23.
b Ref 21.
c Ref 23.

Despite numerous recent studies, the extent to which metabotropic regulation of CAS is mediated by α7 versus α9* nAChRs remains unclear. Furthermore, it is uncertain whether a compound’s agonist or antagonist activity at the ionotropic function of these receptors reliably predicts its ability to modulate CAS.^14,22^ To address these questions, we built on compounds **2**, **3**, and **4**, selective for α9* over α7 nAChRs, to design a focused compound library. Compounds were evaluated alone or in the presence of ACh at (h) α9α10 nAChRs heterologously expressed in *Xenopus laevis* oocytes, quantitatively assessing their ability to activate or suppress acetylcholine (ACh)-evoked responses. For selected compounds of particular interest, pharmacological characterization was expanded to include their effects on homomeric α9 and α7 nAChRs and their role in CAS regulation. The library includes newly synthesized compounds and previously reported ones to explore the impact of steric bulk increases and modifications to the ammonium head (compounds **5**–**13**, Chart [Fig chart1]), elongation of the alkylene linker in **2** (compounds **14** and **15**, Chart [Fig chart1]), hybridization of **2** with **4** (compound **16**, Chart [Fig chart1]), and alterations to the terminal styryl portion of **1** or **2** (compounds **17–22**, Chart [Fig chart1]). This approach aims to refine our understanding of how these modifications influence the ionotropic aspects of the target receptors and whether this correlates with NCNR-mediated CAS modulation.

## Results

### Chemistry

Compounds **6–10** and **12** were synthesized following the procedure outlined in Scheme 1. Commercially available (*E*)-4-hydroxystilbene was *O*-alkylated with 1,2-dibromoethane under basic conditions, yielding compound **23,** which was then converted into compound **24** via a Finkelstein reaction. Commercially available secondary amines were then reacted with intermediate **24** to afford the corresponding tertiary amines (compounds **25–30**). These were subsequently treated with either iodomethane or benzyl bromide to produce the respective quaternary ammonium iodides (compounds **6**, **7**, **8**, **9**, and **12**) or bromide (compound **10**). The synthesis of compounds **1–5**, **11** and **13** followed previously reported methods.^21,23^

**Scheme 1:**
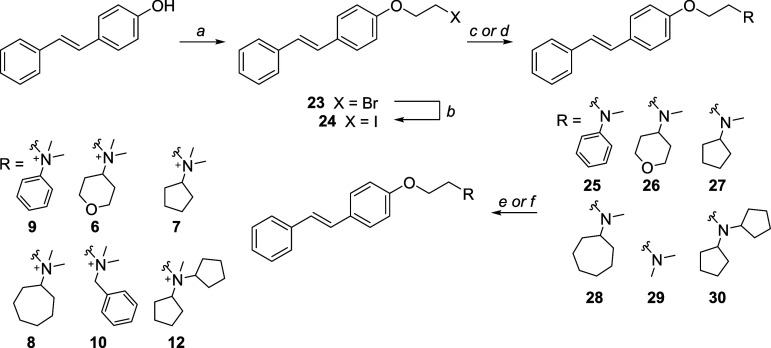
Reagents and Conditions[Fn sch1-fn1]

i (a) 1,2-Dibromoethane, K_2_CO_3_, methylethylketone, reflux; (b) NaI, acetone, reflux; (c) *N*-Methylaniline, K_2_CO_3_, CH_3_CN, reflux (**25**); 5 M dimethylamine in EtOH, THF, 45 °C (**29**); (d) *N*-methyl-*N*-(tetrahydro-2H-pyran-4yl)­amine (**26**), *N*-methylcyclopentanamine (**27**), *N*-methylcycloheptanamine (**28**), dicyclopentylamine (**30**), toluene, 65 °C; (e) iodomethane, CH_2_Cl_2_, rt (**6**, **7**, **8**, **12**) or reflux (**9**); (f) benzyl bromide, THF, reflux (**10**).

Compounds **14** and **15** were synthesized as outlined in Scheme 2. (*E*)-4-Hydroxystilbene underwent a Mitsunobu reaction with 3-chloro-1-propanol, yielding compound **31** or was *O*-alkylated with 1,4-dibromobutane under basic conditions, producing compound **32**. The resulting halo-derivatives were then subjected to a Finkelstein reaction, converting them into the corresponding iodo-derivatives, **33** and **34**. These intermediates were subsequently used to alkylate *N*-methylcyclohexanamine, yielding the tertiary amines **35** and **36**, which were then quaternized to afford compounds **14** and **15**.

**Scheme 2:**
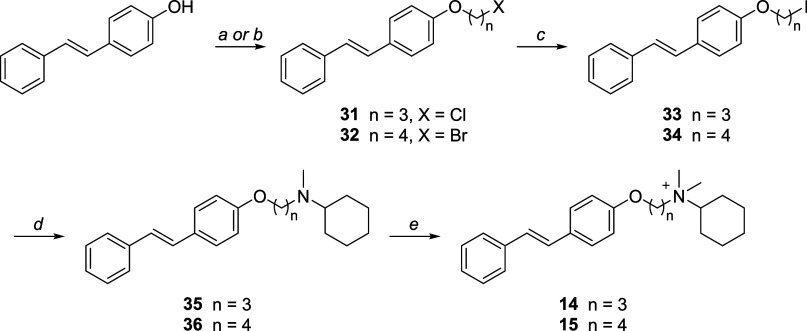
Reagents and Conditions[Fn sch2-fn1]

i (a) 3-Chloro-1-propanol, PPh_3_, DIAD, dry THF, 0 °C–rt (**31**); (b) 1,4-dibromobutane, NaOH, DMSO, rt (**32**); (c) NaI, acetone, reflux; (d) *N*-methylcyclohexanamine, toluene, reflux; (e) iodomethane, CH_2_Cl_2_, rt.

Compound **16** was synthesized as outlined in Scheme 3. *N*-Methylcyclohexanamine was alkylated with compound **24** in toluene, yielding the tertiary amine **37**. This intermediate was then hydrogenated to produce compound **38**, which was subsequently quaternized with iodomethane to afford compound **16**.

**Scheme 3:**
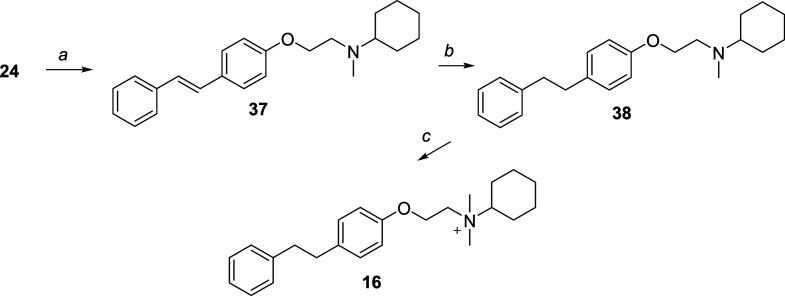
Reagents and Conditions[Fn sch3-fn1]

i (a) *N*-Methylcyclohexanamine, toluene, reflux; (b) H_2_, Pd/C, MeOH, rt; (c) iodomethane, CH_2_Cl_2_, rt.

Compounds **17–20** were synthesized following previously reported methods.^23^

Compound **21** was synthesized as described in Scheme 4. Hydroquinone was first *O*-benzylated to 4-benzyloxyphenol (**39**), which was then *O*-2-bromoethylated to yield compound **40**. This intermediate underwent a Finkelstein reaction to produce the corresponding iodo-derivative (**41**). The resultant compound was then used to alkylate *N*-methylcyclohexanamine, yielding tertiary amine **42**, which was quaternized with iodomethane to afford compound **21**.

**Scheme 4:**
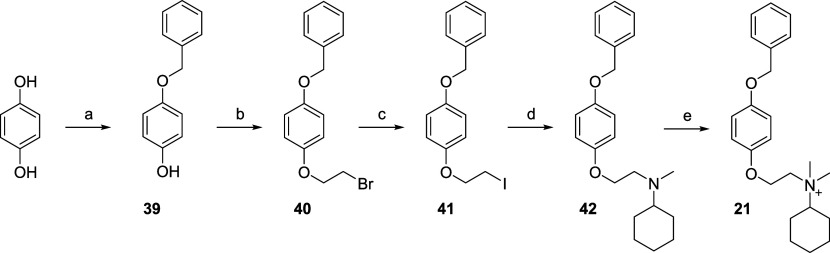
Reagents and Conditions[Fn sch4-fn1]

i (a) K_2_CO_3_, benzyl bromide, acetone, reflux; (b) K_2_CO_3_, 1,2-dibromoethane, acetone, reflux; (c) NaI, acetone, reflux; (d) *N*-methylcyclohexanamine, toluene, reflux; (e) iodomethane, CH_2_Cl_2_, rt.

Compound **22** was synthesized as outlined in Scheme 5. Commercially available 4-hydroxybenzaldehyde was reduced with sodium borohydride, yielding compound **43**. The phenolic moiety was then alkylated with 1,2-dibromoethane (**44**), followed by Finkelstein reaction to produce compound **45.** This intermediate was subsequently used to alkylate *N*-methylcyclohexanamine, affording the tertiary amine **46.** The resulting compound underwent a Mitsunobu reaction with phenol to form compound **47**, which was finally quaternized with iodomethane to yield compound **22**.

**Scheme 5:**
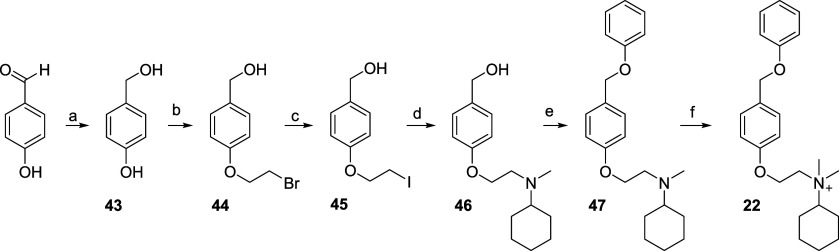
Reagents and Conditions[Fn sch5-fn1]

i (a) NaBH_4_, MeOH, 0 °C—rt; (b) K_2_CO_3_, 1,2-dibromoethane, acetone, reflux; (c) NaI, acetone, reflux; (d) *N*-methylcyclohexanamine, toluene, reflux; (e) phenol, Ph_3_P, DIAD, THF, 0 °C —rt; (f) iodomethane, CH_2_Cl_2_, rt.

### Two-Electrode Voltage Clamp Electrophysiology at Human α7, α9, and α9α10 nAChRs Expressed in *Xenopus* oocytes

The activity of all compounds (**1**–**22**) was first evaluated at the heteromeric hα9α10 nAChR subtype, heterologously expressed in *X. laevis* oocytes, using the two-electrode voltage clamp technique. Additionally, five compounds of the series were tested on the homomeric hα7 and hα9 nAChRs. They were the leads **1** and **2** and the three new compounds combining the enlarged ammonium head of **2** with saturation of vinylene bridge (**16**) or replacement of styryl moiety with benzyloxy (**21**) or phenoxymethyl residue (**22**). All the twenty-two compounds inhibited ACh-evoked currents mediated by the α7, α9, and α9α10 nAChRs (Figures 1 and 2; Tables 1–3). When applied alone (≤30 μM), only compounds **11**, **20**, and **22** failed to activate the hα9α10 receptor (Table 1). Similarly, none of the five selected compounds (**1**, **2**, **16**, **21**, or **22**) induced currents at hα7 (Table 3). In contrast, compound **22** was the only one among these five that did not elicit any current at the hα9 receptor (Table 2). Both antagonistic and agonistic effects were reversible and concentration-dependent across all three receptor subtypes.

**Figure 1:**
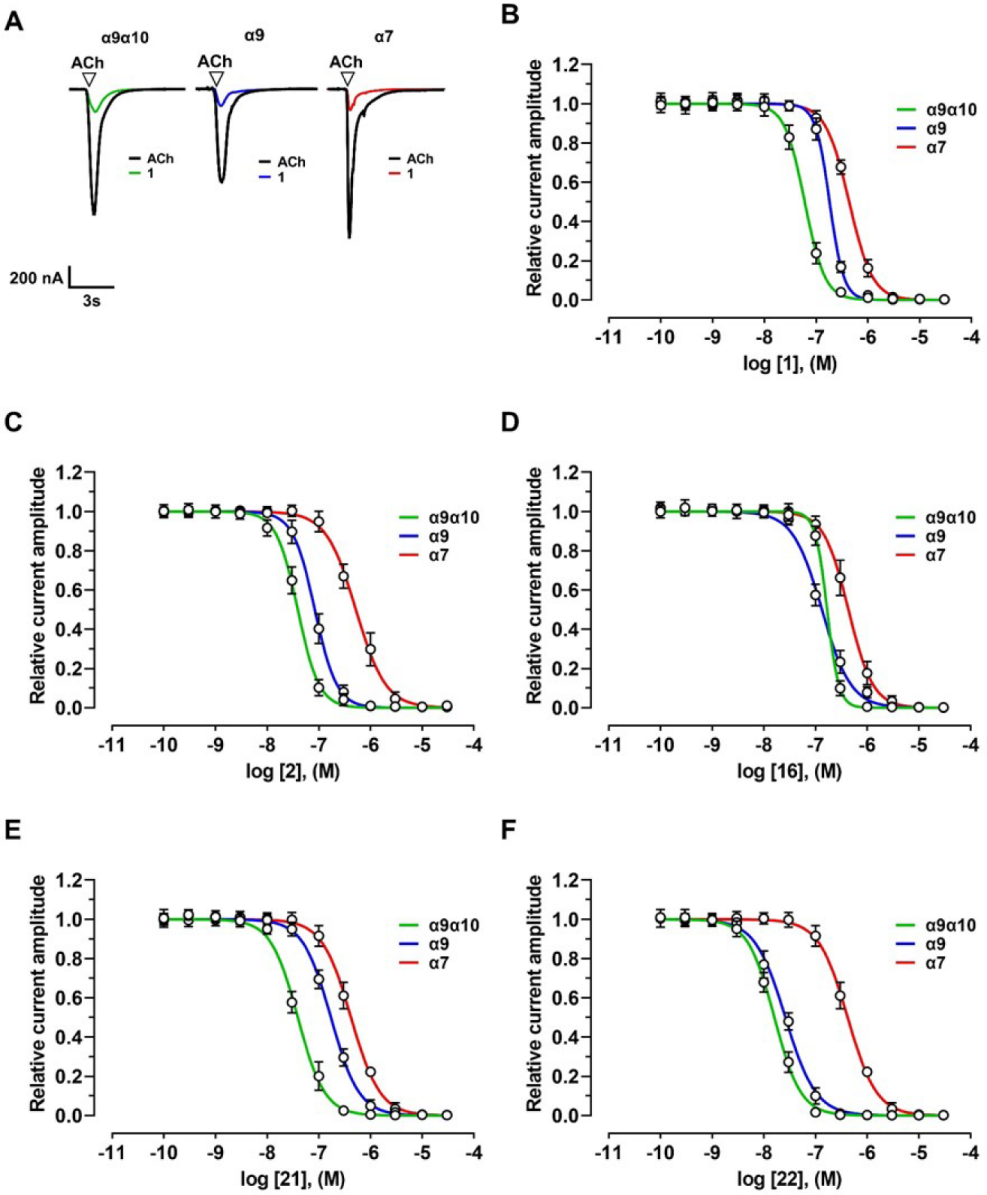
Effects of compounds **1**, **2**, **16**, **21**, and **22** on human (h) α9α10, α9, and α7 nAChRs. (**A**) Representative superimposed ACh-evoked currents recorded at hα9α10, hα9, and hα7 at a holding potential of −80 mV. Currents were elicited by 6 μM (hα9α10), 50 μM (hα9), and 100 μM (hα7) ACh, in the absence and presence of compound **1** at 100 nM, 300 nM, and 1 μM, respectively. ▽ indicates the time of ACh application; Black indicates application of ACh alone; Green, Blue, and Red, represent coapplication of ACh with compound **1** following a 5 min preincubation with compound **1** alone. (**B–F**) Concentration–response relationships for compounds **1** (*n* = 6–12), **2** (*n* = 9–15), **16** (*n* = 6–7), **21** (*n* = 6–9), and **22** (*n* = 6) at hα9α10, hα9, and hα7 nAChRs. Experiments were conducted using 6 μM, 50 μM, and 100 μM ACh, respectively. Current amplitudes were normalized to the response evoked by ACh alone. Corresponding IC_50_ and Hill coefficient (*n*_H_) values are reported in Tables 1 and 2.

**Figure 2:**
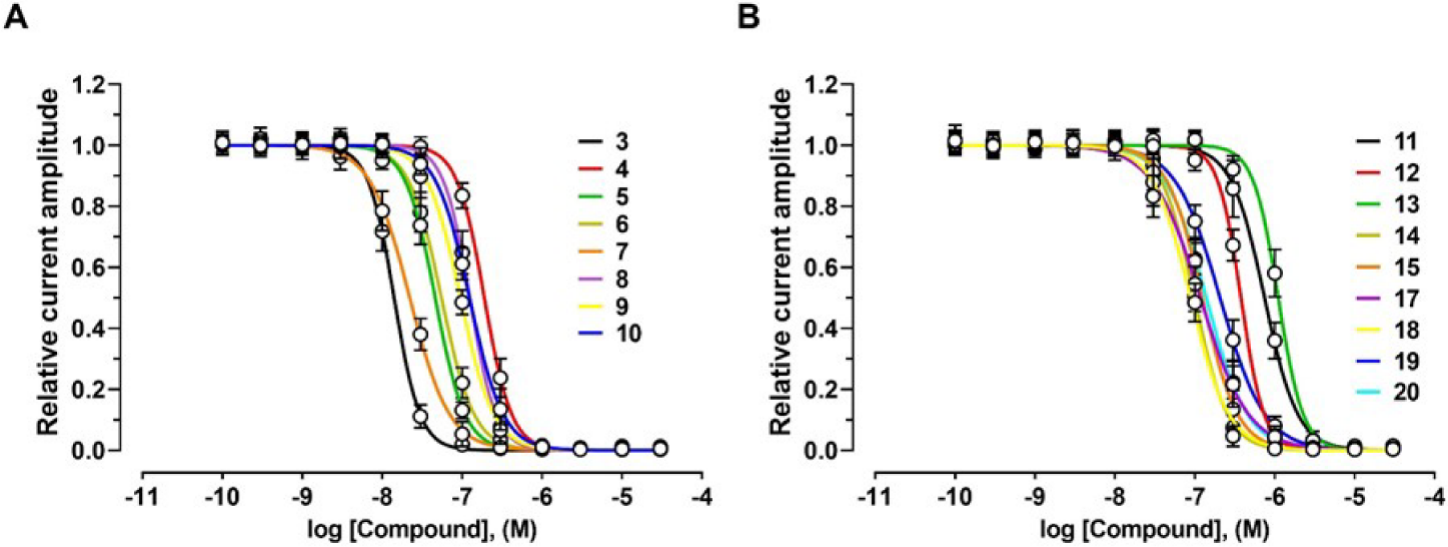
Effects of compounds **3–15** and **17–20** on human (h) α9α10 nAChRs. (**A**, **B**) Concentration–response relationships for compounds **3–15** and **17–20** at hα9α10 nAChRs (*n* = 6–12 oocytes), recorded in the presence of 6 μM ACh. Current amplitudes were normalized to the response elicited by 6 μM ACh alone. The corresponding IC_50_ and Hill coefficient (*n*_H_) values are presented in Table 1.

**Table 1:** Pharmacological Activity of Compounds (Cpd) at hα9α10 nAChRs^a^,^b^,^c^,^f^

Cpd	IC_50_ ^d^ (nM)	*n*_H_ ^d^	IC_50_ ratio to Cpd 1	EC_50_ ^e^ (nM)	*n*_H_ ^e^	EC_50_ ratio to Cpd 1
**1**	60.2	2.2	1.0	385.3	3.9	1.0
	(57.9–62.6)	(2.1–2.4)		(364.1–406.5)	(3.3–4.5)	
	[12]	[12]		[5]	[5]	
**2**	39.1	2.1	0.6	272.7	3.9	0.7
	(37.6–40.6)	(1.9–2.3)		(264.6–287.7)	(3.3–4.5)	
	[11]	[11]		[7]	[7]	
**3**	14.1	2.7	0.2	200.3	2.3	0.6
	(13.6–14.6)	(2.5–2.9)		(186.1–215.2)	(2.0–2.6)	
	[10]	[10]		[5]	[5]	
**4**	189.5	2.5	3.1	1140.0	1.2	3.0
	(183.0–196.2)	(2.4–2.7)		(1037.0–1253.0)	(1.1–1.3)	
	[8]	[8]		[5]	[5]	
**5**	46.0	2.3	0.8	217.6	4.5	0.6
	(44.4–47.7)	(2.2–2.5)		(203.6–231.6)	(3.8–5.2)	
	[10]	[10]		[5]	[5]	
**6**	55.0	2.1	0.9	184.8	2.4	0.5
	(52.7–57.5)	(2.0–2.3)		(176.0–194.1)	(2.2–2.7)	
	[6]	[6]		[6]	[6]	
**7**	22.1	1.7	0.4	174.8	1.6	0.5
	(20.9–23.4)	(1.6–1.9)		(163.–187.4)	(1.4–1.7)	
	[6]	[6]		[6]	[6]	
**8**	124.5	2.6	2.1	300.1	1.6	0.8
	(119.2–129.9)	(2.3–2.9)		(277.2–325.6)	(1.4–1.8)	
	[7]	[7]		[6]	[6]	
**9**	95.1	2.1	1.6	473.7	3.3	1.2
	(91.9–98.3)	(1.9–2.2)		(457.5–490.0)	(3.1–3.6)	
	[12]	[12]		[6]	[6]	
**10**	123.5	2.1	2.1	448.5	2.4	1.2
	(118.4–128.9)	(1.9–2.2)		(431.8–465.9)	(2.2–2.5)	
	[6]	[6]		[6]	[6]	
**11**	745.2	2.1	12.4	No activity		
	(703.8–789.0)	(1.9–2.3)				
	[7]	[7]				
**12**	378.0	3.0	6.3	1707.0	1.2	4.4
	(363.9–392.7)	(2.7–3.4)		(1580.0–1843.0)	(1.1–1.3)	
	[6]	[6]		[6]	[6]	
**13**	1106.0	2.8	18.4	7044.0	2.7	18.3
	(1063.0–1150.0)	(2.4–3.2)		(6659.0–7449.0)	(2.4–3.0)	
	[7]	[7]		[5]	[5]	
**14**	98.9	2.1	1.6	511.2	3.0	1.3
	(95.0–103.0)	(1.9–2.3)		(488.2–535.2)	(2.8–3.2)	
	[8]	[8]		[7]	[7]	
**15**	124.2	2.0	2.1	1470.0	1.4	3.8
	(118.9–129.8)	(1.8–2.1)		(1369.0–1579.0)	(1.2–1.5)	
	[11]	[11]		[5]	[5]	
**16**	167.8	3.8	2.8	482.5	3.2	1.3
	(161.1–174.8)	(3.5–4.1)		(463.2–502.2)	(3.0–3.5)	
	[6]	[6]		[6]	[6]	
**17**	117.3	1.4	1.9	479.1	3.3	1.2
	(110.9–124.1)	(1.3–1.6)		(459.1–499.4)	(3.1–3.6)	
	[10–11]	[10–11]		[5]	[5]	
**18**	89.0	1.8	1.5	550.3	2.2	1.4
	(84.5–93.6)	(1.7–2.0)		(517.6–585.1)	(2.0–2.4)	
	[8–10]	[8–10]		[7]	[7]	
**19**	206.8	1.6	3.4	1016.0	1.9	2.6
	(196.1–218.1)	(1.4–1.7)		(941.6–1094.0)	(1.7–2.2)	
	[7]	[7]		[5]	[5]	
**20**	138.8 (133.2–144.6) [8]	1.7 (1.6–1.8) [8]	2.3	No activity		
**21**	39.1	1.7	0.6	338.2	3.7	0.9
	(36.9–41.5)	(1.5–1.8)		(326.2–350.6)	(3.1–4.2)	
	[6]	[6]		[6]	[6]	
**22**	16.0	1.7	0.3	No activity		
	(15.3–16.8)	(1.6–1.8)				
	[6]	[6]				

a EC_50_, half-maximal effective concentration.

b IC_50_, half-maximal inhibitory concentration.

c *n*_H_, Hill coefficient.

d Values ± 95% CI (confidence interval) were calculated fromFigures [Fig fig1] and 2.

e Values ± 95% CI were calculated from Figures 3 and 4.

f Number in square brackets indicates the number of oocytes used.

**Table 2:** Pharmacological Activity of Compounds (Cpd) at hα9 nAChRs^a^,^b^,^c^,^f^

	α9					
Cpd	IC_50_ ^d^ (nM)	*n*_H0_ ^d^	IC_50_ ratio to Cpd 1	EC_50_ ^e^ (nM)	*n*_H_ ^e^	EC_50_ ratio to Cpd 1
**1**	181.9	3.2	1.0	921.4	2.1	1.0
	(174.0–190.1)	(2.9–3.4)		(860.4–987.5)	(1.8–2.4)	
	[6]	[6]		[5]	[5]	
**2**	83.6	2.1	0.5	354.9	2.3	0.4
	(80.4–86.9)	(1.9–2.2)		(326.3–390.7)	(1.9–2.9)	
	[9]	[9]		[6]	[6]	
**16**	132.9	1.5	0.7	982.6	2.4	1.1
	(125.1–141.2)	(1.4–1.7)		(960.0–1005.0)	(2.2–2.6)	
	[6]	[6]		[5]	[5]	
**21**	170.9	1.6	0.9	905.2	2.2	1.0
	(164.2–177.9)	(1.5–1.7)		(839.3–972.0)	(1.9–2.6)	
	[9]	[9]		[5]	[5]	
**22**	26.2	1.5	0.1	No activity		
	(24.7–27.7)	(1.4–1.6)				
	[6]	[6]				

a EC_50_, half-maximal effective concentration.

b IC_50_, half-maximal inhibitory concentration.

c *n*_H_, Hill coefficient.

d Values ±95% CI (confidence interval) were calculated from Figure 1.

e Values ±95% CI were calculated from Figure 3.

f Number in square brackets indicates the number of oocytes used.

**Table 3:** Pharmacological Activity of Compounds (Cpd) at hα7 nAChRs^a^,^b^,^c^,^e^,^f^

	α7					
Cpd	IC_50_ ^d^ (nM)	*n*_H0_ ^d^	IC_50_ ratio to Cpd 1	EC_50_ (nM)	*n*_H_	EC_50_ ratio to Cpd 1
**1**	433.3	1.9	1.0	No activity		
	(414.7–452.7)	(1.8–2.1)				
	[6]	[6]				
**2**	520.7	1.5	1.2	No activity		
	(497.5–545.1)	(1.4–1.6)				
	[14–15]	[14–15]				
**16**	433.5	1.8	1.0	No activity		
	(408.1–460.6)	(1.7–2.0)				
	[6–7]	[6–7]				
**21**	418.8	1.5	1.0	No activity		
	(393.8–445.4)	(1.4–1.7)				
	[6]	[6]				
**22**	336.2	1.5	0.8	No activity		
	(317.9–355.7)	(1.4–1.7)				
	[6]	[6]				

a EC_50_, half-maximal effective concentration.

b IC_50_, half-maximal inhibitory concentration.

c *n*_H_, Hill coefficient.

d Values ± 95% CI (confidence interval) were calculated from Figure 1.

e Values ± 95% CI were calculated from Figure 3.

f Number in square brackets indicates the number of oocytes used.

In the presence of ACh, compound **1** acted as an antagonist at both hα9 and hα9α10 receptors, with half-maximal inhibitory concentration (IC_50_) values of 181.9 nM and 60.2 nM, respectively (Figure 1A,B, Tables 1 and 2). When applied alone, compound **1** reversibly potentiated currents at α9-containing nAChRs, with half-maximal effective concentration (EC_50_) values of 921.4 nM for hα9 and 385.3 nM for hα9α10 nAChRs (Figure 3A,B, Tables 1 and 2). At the hα7 subtype, compound **1** acted solely as an antagonist with an IC_50_ of 433.3 nM (Table 3).

**Figure 3:**
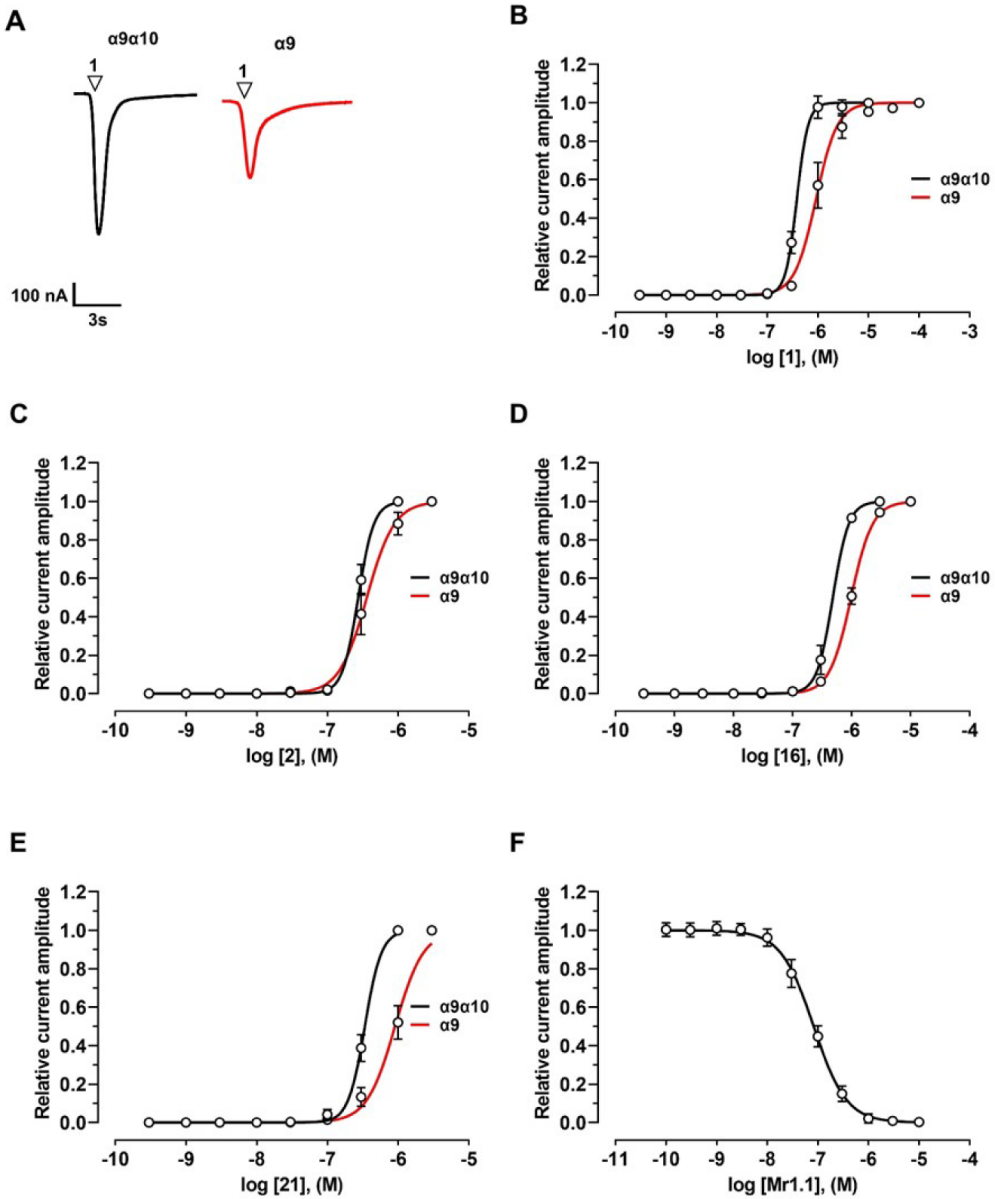
Effects of compounds **1**, **2**, **16**, and **21** on human (h) α9α10 and α9 nAChRs. (**A**) Representative currents evoked by 1 μM compound **1** at hα9α10 and hα9 receptors, recorded at a holding potential of −80 mV. White triangle indicates the time of compound **1** application. (**B–E**) Concentration–response relationships for compounds **1** (*n* = 5), **2** (*n* = 6–7), **16** (*n* = 5–6), and **21** (*n* = 6–9) at hα9α10 and hα9 nAChRs. Current amplitudes were normalized to the response evoked by the highest concentration of each respective compound. EC_50_ and Hill coefficient (*n*_H_) values are provided in Table 1 and 2. (**F**) Concentration–response relationships for α-conotoxin Mr1.1 (*n* = 15) at hα9α10 nAChRs in the presence of 300 nM compound **2.** The resulting IC_50_ was 82.9 nM (95% CI: 79.1–86.9), with a Hill coefficient (*n*_H_) of 1.3 (95% CI: 1.3–1.4). Current amplitudes were normalized to the response elicited by 300 nM compound **2**.

Compound **2** displayed a pharmacological profile similar to compound **1** but with greater preference for α9 and α9α10 subtypes. It inhibited ACh-evoked currents with IC_50_ values of 83.6 nM and 39.1 nM, respectively (Figure 1C, Tables 1 and 2). At hα7, inhibition was weaker (IC_50_ = 520.7 nM) (Table 3). When applied alone, compound **2** evoked currents with EC_50_ values of 354.9 nM at hα9 and 272.2 nM at hα9α10 (Figure 3C, Tables 1 and 2). Notably, currents elicited by 300 nM compound **2** at hα9α10 (near its EC_50_ value) were inhibited by the hα9α10 selective α-conotoxin Mr1.1, with an IC_50_ of 82.9 nM (Figure 3F).^24^

At the α9α10 nAChR, compound **3** exhibited the highest potency with IC_50_ and EC_50_ values of 14.1 nM and 200.3 nM, respectively. Compound **4** showed weaker activity (IC_50_ = 189.5 nM and EC_50_ = 1140.0 nM). Similarly, compound **5** inhibited the α9α10 receptor with an IC_50_ of 46.0 nM and activated with an EC_50_ of 217.6 nM (Figures 2A, 4A and Table 1).

**Figure 4:**
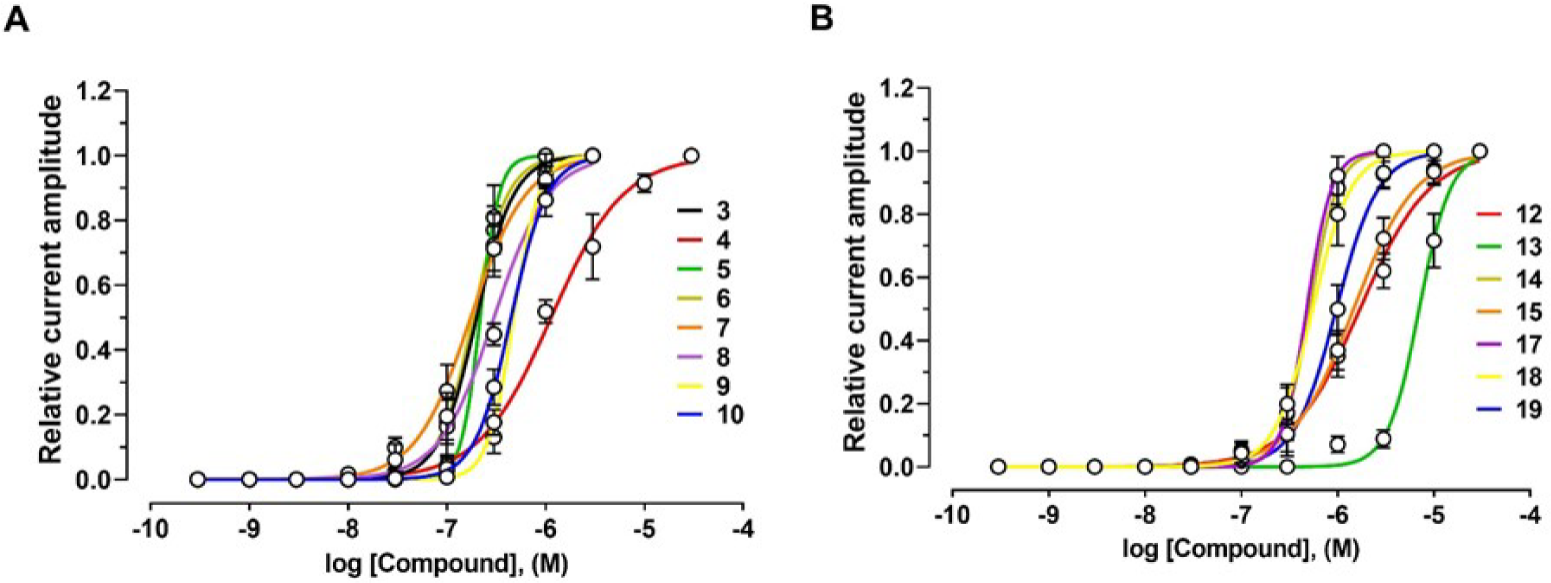
Effects of compounds **3–10**, **12**–**15**, and **17**–**19** on human (h) α9α10 nAChRs. (A, B) Concentration–response relationships for compounds **3–10**, **12**–**15**, and **17**–**19** at hα9α10 nAChRs (*n* = 5–7 oocytes per compound). Current amplitudes were normalized to the maximum response evoked by the highest concentration of each respective compound. The corresponding EC_50_ and Hill coefficient (*n*_H_) values are summarized in Table 1.

Compounds **6** and **7** showed similar dual activities, antagonizing α9α10 with IC_50_ values of 55.0 nM and 22.1 nM, respectively. The EC_50_ values at α9α10 nAChRs were also similar: 184.8 nM for compound **6** and 174.8 nM for compound **7** (Figures 2A, 4A and Table 1).

Compounds **8**, **9**, and **10** displayed comparable inhibitory potency at hα9α10 with IC_50_ values of 124.5 nM, 95.1 nM, and 123.5 nM, respectively. Their EC_50_ values for current induction were also within a similar range: 300.1 nM, 473.7 nM, and 448.5 nM, respectively (Figures 2A, 4A and Table 1). In contrast, compound **11** antagonized ACh-evoked α9α10 currents (IC_50_ = 745.2 nM) but lacked agonist activity (Figure 2B and Table 1).

Compounds **12** and **13** were weak agonists at α9α10 nAChRs, with EC_50_ values of 1707 nM and 7044 nM, respectively, while exhibiting inhibitory effects with IC_50_ values of 378 nM and 1106 nM (Figures 2B, 4B; Table 1). In comparison, compounds **14** and **15** were more potent antagonists at α9α10 receptors (IC_50_ = 98.9 nM and 124.2 nM, respectively), with stronger agonist activity (EC_50_ = 511.2 nM and 1470.0 nM, respectively) (Figures 2B, 4B; Table 1).

Compound **16** displayed similar potencies across hα9 and hα9α10 nAChRs, inhibiting with IC_50_ values of 132.9 nM and 167.8 nM, and activating with EC_50_ values of 982.6 nM and 482.5 nM, respectively (Figures 1D, 3D). However, compound **16** only inhibited hα7 nAChRs (IC_50_ = 433.5 nM) and showed no agonist activity (Table 3).

Compounds **17**, **18**, and **19** inhibited α9α10-mediated ACh currents with IC_50_ values of 117.3 nM, 89.0 nM, and 206.8 nM, respectively (Figure 2B; Table 1). They also activated the receptor with EC_50_ values of 479.1 nM, 550.3 nM, and 1016.0 nM, respectively (Figure 4B; Table 1). Conversely, compound **20** acted only as an antagonist (IC_50_ = 138.8 nM) and failed to evoke currents.

Compounds **21** and **22** selectively inhibited ACh-evoked currents mediated by α9-containing nAChRs (Figure 1E,F). Compound **21** exhibited IC_50_ values of 170.9 nM at hα9 and 39.1 nM at hα9α10, with weaker inhibition at hα7 (IC_50_ = 418.8 nM). In contrast, compound **22** was more potent across all three nAChR subtypes, with IC_50_ values of 16.0 nM at hα9α10, 26.2 nM at hα9, and 336.3 nM at hα7 (Tables 1–3). Compound **21** also acted as an agonist at α9 and α9α10 nAChRs, with EC_50_ values of 905.2 nM and 338.2 nM, respectively (Figure 3E), but not at hα7. In contrast, compound **22** lacked agonist activity at all three subtypes.

### Effects of Compounds 1, 2, 16, 21, and 22 on Interleukin (IL)-1β Release by Human Monocytic THP-1 Cells

Five compounds (**1**, **2**, **16**, **21**, and **22**), fully characterized for their agonist and antagonist activity at the conventional ionotropic α9α10, α9, and α7 nAChRs, were evaluated for their effects on the ATP-mediated interleukin-1β (IL-1β) release by human monocytic THP-1 cells.

Monocytic THP-1 cells were first primed with lipopolysaccharide (LPS, 1 μg/mL for 5 h), followed by stimulation with (2′(3′)-*O*-(4-benzoyl–benzoyl) ATP (BzATP, 100 μM) to induce the maturation and release of IL-1β. As expected,^22,25^ LPS alone did not trigger a significant release of IL-1β into the cell culture supernatant, whereas stimulation with BzATP led to a marked increase in IL-1β levels. IL-1β concentrations in supernatants from LPS-primed and BzATP-stimulated cells were normalized to 100% (Figure 5A–D). When ACh (10 μM) was applied shortly before BzATP, IL-1β release was significantly reduced (Figure 5A–D), consistent with previous findings.^25^

**Figure 5:**
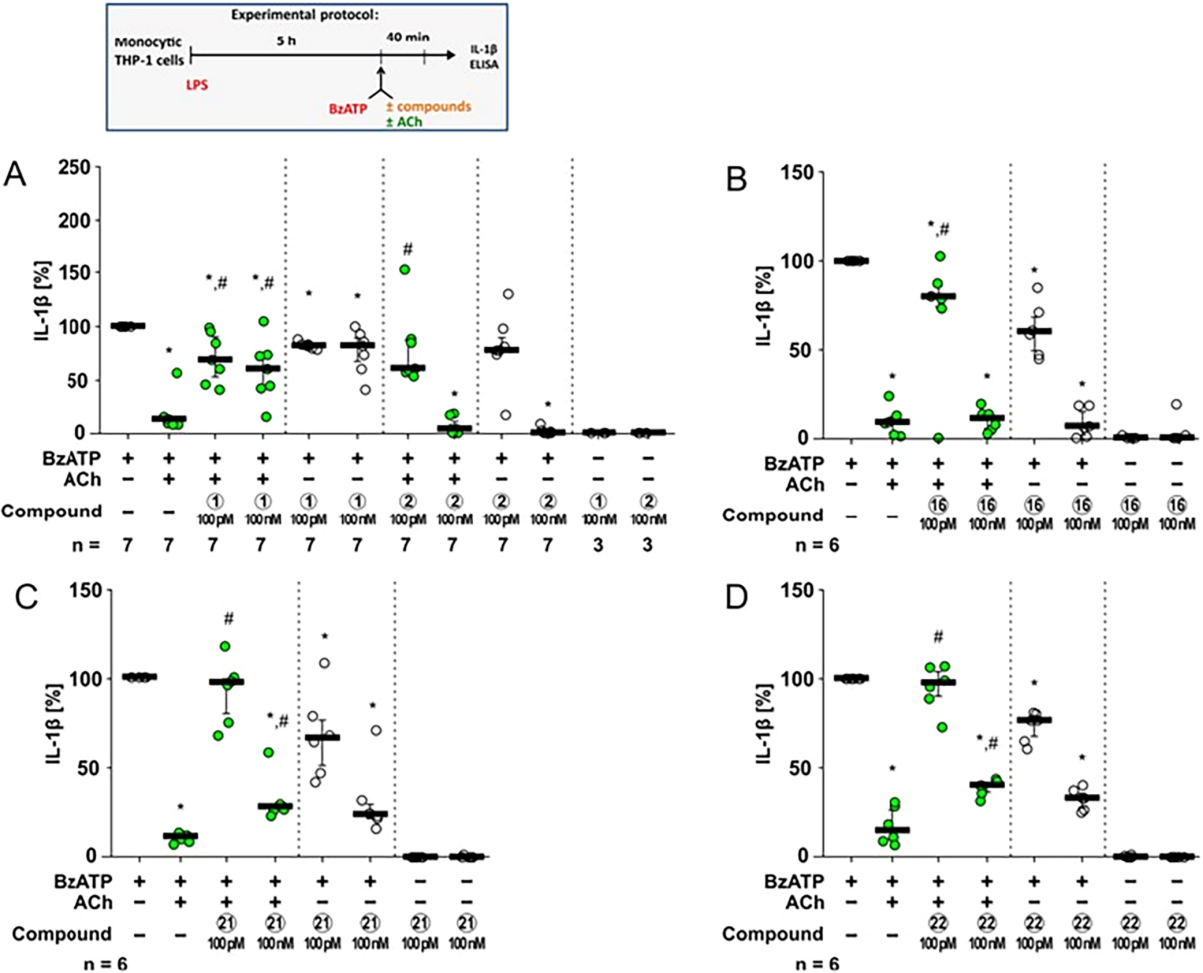
Effects of compounds **1**, **2**, **16**, **21**, and **22** on BzATP-induced release of interleukin-1β (IL-1β) from human monocytic THP-1 cells. THP-1 cells were primed with lipopolysaccharide (LPS; 1 μg/mL, 5 h) followed by stimulation with the P2X7 receptor agonist BzATP (2′/3′-O-(4-benzoylbenzoyl)­adenosine-5′-triphosphate, tri­(triethylammonium) salt) for 40 min to induce IL-1β release, which was quantified by ELISA. The effects of acetylcholine (ACh; 10 μM), compounds **1** (A), **2** (A), **16** (B), **21** (C), and **22** (D), and their combinations with ACh on IL-1β release were evaluated. Of note, all samples containing ACh are highlighted in green. IL-1β concentrations were calculated by subtracting values measured in LPS-only controls. For each experiment, IL-1β release induced by BzATP + solvent was set to 100%, and all other values were expressed relative to this reference. Data are presented as individual data points; bars represent the median and whiskers indicate the 25th to 75th percentile. *p ≤ 0.05 vs LPS + BzATP; #*p* ≤ 0.05 vs LPS + BzATP + ACh. Statistical analysis was performed using the Friedman test followed by the Wilcoxon signed-rank test.

Compounds **1**, **2**, **16**, **21**, and **22** were tested at 100 pM and 100 nM concentrations in the absence or presence of ACh (10 μM). Compound **1** efficiently antagonized the inhibitory effect of ACh on the BzATP-induced release of IL-1β at both high (100 nM) and low (100 pM) concentrations (Figure 5A). In the absence of ACh, compound **1** only slightly reduced the BzATP-induced release of IL-1β (Figure 5A). Similarly, low concentrations (100 pM) of compounds **2**, **16**, **21**, and **22** efficiently antagonized the inhibitory effect of ACh (Figure 5A–D). At higher concentrations (100 nM), mild antagonistic effects were seen only for compounds **21**, and **22** (Figure 5C,D). In contrast to compound **1**, high concentrations (100 nM) of compounds **2**, **16**, **21**, and **22** effectively and significantly inhibited the BzATP-induced release of IL-1β in the absence of ACh, while at the low concentration (100 pM) a minor reduction was seen for compounds **16**, **21**, and **22** (Figure 5B–D). These data suggest that compounds **2**, **16**, **21**, and **22** exert both antagonistic and agonistic functions at the NCNRs of monocytic cells. Notably, in none of the experimental settings, cell death was increased, as measured by the lactate dehydrogenase activity in cell culture supernatants (data not shown).

### Molecular Dynamics (MD) Simulations of Compounds 21 and 22 at the Homopentameric Human α9 nAChR: A Concentration-Dependent Antagonist-to-Agonist Switch

To elucidate the molecular basis for the concentration-dependent activity switch of compound **21**, which functions as an antagonist at low concentration and as an agonist at higher concentrations at the homopentameric hα9 nAChR, molecular dynamics (MD) simulations were conducted. For comparison, compound **22**, which does not exhibit agonist activity even at high concentrations, was also modeled for comparison. Understanding the mechanism underlying the switching behavior of compound **21** may provide a general framework for interpreting dual activity at α9-containing nAChRs.

Docking calculations positioned compound **21** at the canonical agonist binding site, revealing a compact, π-stacked ring conformation within the extracellular domain (ECD) binding pocket (Figure 6A,B). To relax this strained pose and examine its impact on receptor conformation, MD simulations were performed. A key marker of nAChR activation, the closure of loop C (particularly the Cys-Cys motif) of the principal (+) subunit toward the complementary (−) subunit, was monitored by measuring the distance between the Cα atoms of C194 at α9­(+) and T38 at α9(−). This distance serves as a proxy for agonist or antagonist behavior and is consistent with previous MD studies of ligand-nAChR interactions.^26^

**Figure 6:**
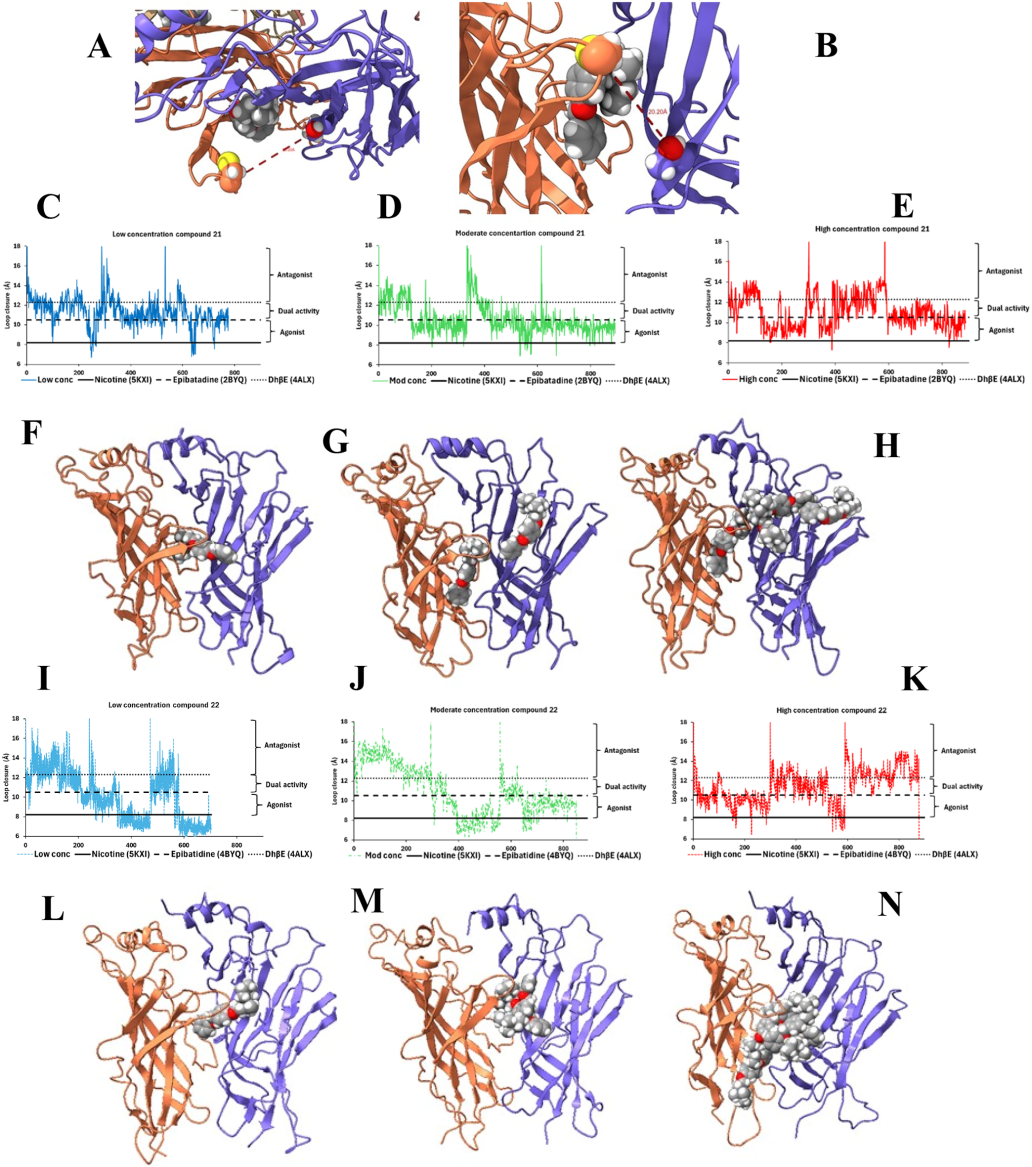
Concentration-dependent modulation of human (h) α9 nAChR loop C conformation by compounds **21** and **22**. (A) Top view of the ECD of the homopentameric hα9 nAChR shown as ribbons and colored by subunit (orange: principal subunit; blue: complementary subunit), with compound **21** docked at the ligand-binding site. The ligand and the Cα atoms of residues C194 (principal subunit) and T38 (complementary subunit) are shown as spheres. Atom colors: yellow = S, gray = C, white = H, red = O, orange = Cα of C194. The red dashed line represents the C194-T38 Cα distance, used as a structural proxy to distinguish agonist- from antagonist-like binding modes. (B) Side view of the ECD with compound **21** initially docked in the canonical agonist binding site. (C–E) Time series plots of the C194-T38 Cα distance at (C) low, (D) moderate, and (E) high concentrations of compound **21**. Reference distances based on known structures of nAChR bound to nicotine (5KXI), AChBP bound to epibatidine (2BYQ) or dihydro-β-erythroidine (DhβE) (4ALX) are shown as solid, dashed, and dotted black lines, respectively; corresponding PDB ID codes are indicated. (F–H) Representative MD simulation snapshots of compound **21** bound to the α9 ECD at (F) low, (G) moderate, and (H) high concentrations. Time series plots of loop C closure for compound **22** are also shown for (I) low, (J) moderate, and (K) high concentrations, with representative MD simulation snapshots of compound **22** bound to the α9 ECD at (L) low, (M) moderate, and (N) high concentrations.

To simulate different ligand concentrations, two approaches were employed: (1) At low concentrations, ligands were docked to the five intersubunit agonist binding sites at the pentameric ECD, and (2) at moderate and high concentrations, “flooding” simulations were performed, in which 5 or 15 additional ligands were randomly distributed in the solvent near the ECD. It is noted that each homomeric hα9 nAChR complex contains five equivalent intersubunit interfaces. We calculated the C194-T38 distance for each subunit interface at every time step and used the minimum distance among the five as a representative measure of the smallest observed loop C closure over time. Concatenated time series plots of this minimum C194-T38 distance (loop C closure) across the five subunits and triplicate runs at low and moderate to high concentrations (Figure 6C-E for compound **21**; and 6I–K for compound **22**) were compared with benchmark distances associated with known ligands bound to ACh binding protein (AChBP) or other nAChR subtypes. These included agonist (epibatidine; 2BYQ),^27^ antagonists (dihydro-β-erythroidine; 4ALX)^28^ and dual-activity ligands (nicotine; 5KXI),^29^ indicated by solid and dashed lines. For compound **21**, at low concentration, distances mostly remained in the 10–12 Å range, occasionally exceeding 12 Å, consistent with antagonist or dual-activity profiles (Figure 6C). In contrast, at moderate (Figure 6D) and high (Figure 6E) concentrations, loop C closure frequently fell within the 8–10 Å range, characteristic of agonist-like conformations.

Analysis of ligand poses from the MD trajectories revealed concentration-dependent changes in compound **21** orientation within the agonist binding pocket. At low concentrations (Figure 6F), the ligand orients perpendicular to the long axis of the ECD, similar in manner to compound **22** (Figure 6L). In contrast, at moderate (Figure 6G) and high (Figure 6H) concentrations, compound **21** aligns parallel to this axis. Notably, at moderate concentrations, a single additional ligand was often observed binding near the tip of loop C (close to the Cys-Cys motif), whereas at high concentrations, multiple ligands aggregated at this site. These interactions appear to stabilize loop C in a more closed agonist-like conformation, providing a plausible mechanism for the observed concentration-dependent switch in activity.

In contrast, MD simulations of compound **22** showed a different binding profile. C194-T38 distance plots for compound **22** (Figure 6I–K) demonstrated that loop C closure distance frequently exceeded 12.3 Å and also fell below 8 Å at all tested concentrations. At moderate and high concentrations (Figure 6M,N), rather than bind to a distinct allosteric site at the complementary subunit, compound **22** tended to form closely bound oligomeric aggregates which insert together inside loop C. This distinct conformational behavior supports the observation that compound **22** exerts different functional consequences on hα9 compared to compound **21**.

### Prediction of Physicochemical Properties

To evaluate the drug-likeness of compound **2** and optimized compounds **21** and **22**, physicochemical, medicinal chemistry, and pharmacokinetic properties were predicted using the SwissADME web tool (Table 4 and Table S1).^30^ The bioavailability radar generated by SwissADME for compound **2** (Figure 7) revealed two suboptimal physicochemical properties, namely XLOGP3 greater than 5 (compound **2**, XLOGP3 = 6.03) and topological polar surface area (TPSA) lower than 20 Å^2^ (compound **2**, TPSA = 9.23 Å^2^). In contrast, the bioavailability radar for compounds **21** and **22** (XLOGP3 = 5.22, TPSA = 18.46 Å^2^) indicate that both XLOGP3 and TPSA have been improved toward optimal parameters for oral bioavailability (Table 4 and Figure 7). Compounds **2**, **21**, and **22** were predicted by SwissADME to be compliant with Lipinski, Ghose, Veber, and Egan rules, with only a single violation of the Muegge rule (Table S1). Moreover, pan-assay interference compounds (PAINS) analysis of these molecules did not identify any problematic fragments, while Brenk filters detected two alerts for compound **2** (i.e., stilbene and quaternary nitrogen), while only one alert for compounds **21** and **22** (i.e., quaternary nitrogen) (Table S1).

**Figure 7:**
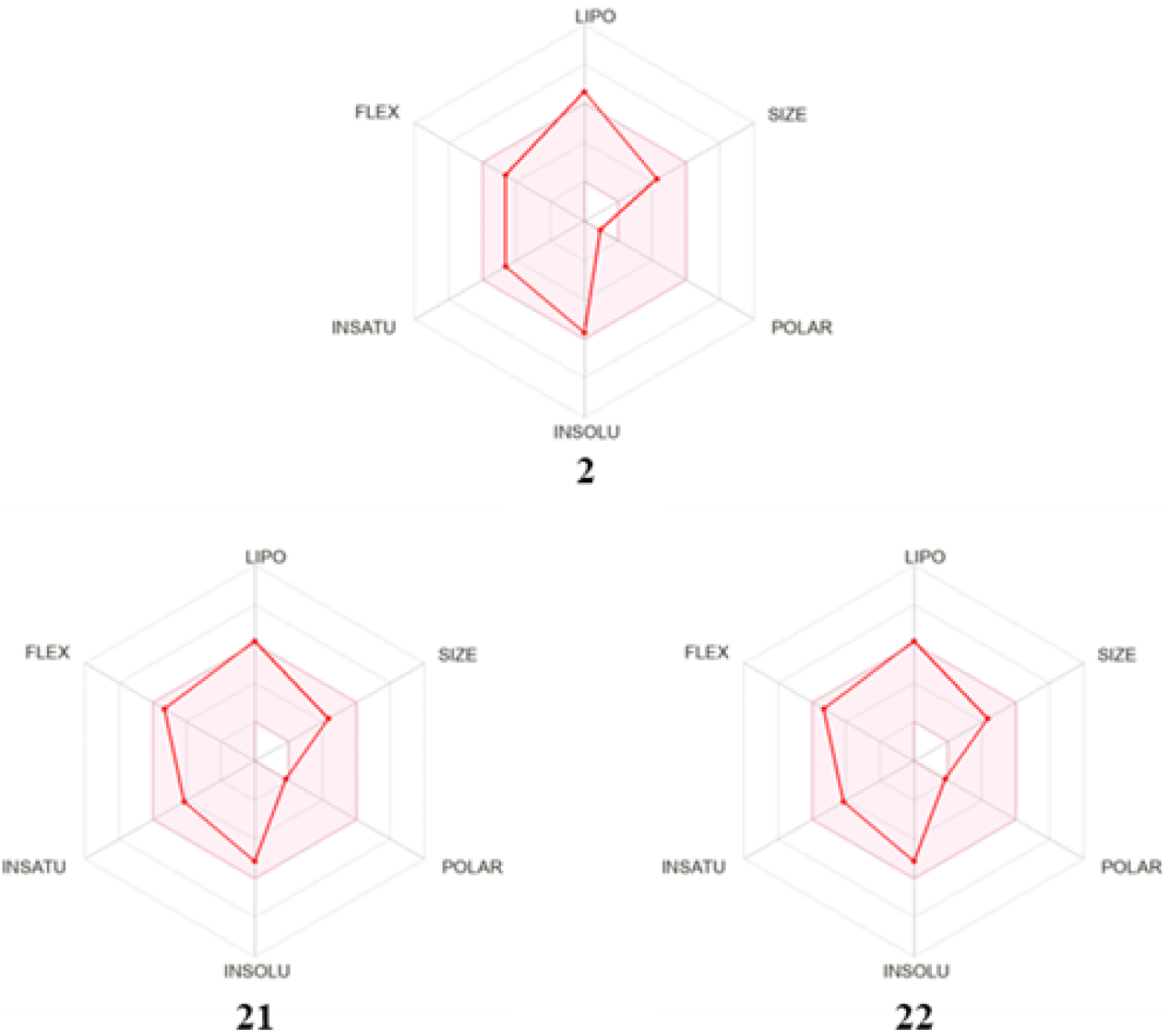
Bioavailability radar of compounds **2**, **21**, and **22**. The pink area represents the optimal range for each physicochemical property: lipophilicity (XLOGP3 between −0.7 and +5.0), size (MW between 150 and 500 g/mol), polarity (TPSA between 20 Å^2^ and 130 Å^2^), solubility (logS not higher than 6), saturation (fraction sp^3^ carbons ≥0.25), and flexibility (≤9 rotatable bonds). The red line (radar plot) represents value of each property for the compound being taken into consideration.

**Table 4:** Predicted Properties of Compounds **2**, **21**, and **22** Considered in the Bioavailability Radar

Compound	XLOGP3	MW (g/mol)	TPSA (Å^2^)	Log S	*n* rotatable bonds	Fraction sp^3^
**2**	6.03	350.52	9.23	–5.69	7	0.42
**21**	5.22	354.51	18.46	–5.14	8	0.48
**22**	5.22	354.51	18.46	–5.14	8	0.48

## Discussion

The present study provides a comprehensive pharmacological characterization of a novel panel of 22 small molecules targeting human α7, α9, and α9α10 nAChR subtypes expressed in *X. laevis* oocytes. The compounds display a broad range of activity profiles, including both concentration-dependent agonist and antagonist actions, some of which are subtype-selective. Notably, compounds **1**, **2**, **16**, **21,** and **22**, exhibited selective antagonism toward α9-containing nAChRs over α7 nAChRs, with the strongest selectivity observed for compounds **2**, **21**, and **22** at α9α10 nAChRs. Notably, except for compound **22**, these compounds elicited detectable currents only from α9-containing nAChRs. Several of these compounds show promise as chemical probes or as scaffolds for the development of α9- and α9α10-selective therapeutics. Importantly, our findings demonstrate the modulatory potential of these small molecules on α9α10 nAChRs, revealing subtype-specific inhibition patterns reminiscent of marine-derived α-conotoxins such as RgIA and Vc1.1. These disulfide-rich peptides are known to bind an allosteric site at the α9­(+)/α10­(−) interface, distinct from the canonical orthosteric binding sites targeted by agonists like ACh or nicotine.^31–34^ Our results suggest that certain compounds with activity profiles such as compound **21**, may engage a similar allosteric domain, stabilizing a nonconducting state of the receptor through interactions within the extracellular vestibule or via deeper allosteric coupling pathways, as proposed for other disulfide-rich peptides.

### Dual Agonist/Antagonist Activity at α9-Containing Receptors

All compounds tested at hα9α10 nAChRs exhibited dual, concentration-dependent actions: low nanomolar IC_50_ values (ranging between 14 and 380 nM) for antagonism and EC_50_ values for agonism that were two-to-14 higher. Exceptions included **11**, **20**, and **22**, which lacked agonist activity, and compound **13**, which showed only weak, supra-micromolar activity as both agonist and antagonist.

Compounds **1–4** have previously been reported to inhibit ACh-evoked currents at hα9α10 nAChRs expressed in *X. laevis* oocytes.^21,23,35^ The IC_50_ value for compound **1** in our study is consistent with earlier reports (10 nM in the presence of 6 μM and 10 μM ACh). In contrast, compounds **2–4** were previously assessed only under saturating ACh conditions (1 mM), likely obscuring their full antagonist potency. Indeed, their previously reported IC_50_ values at 1 mM ACh were over 45-fold higher than those observed under lower ACh conditions in the present study.^21,23^ Although earlier work suggested that compounds **2–4** could weakly activate α9α10 nAChRs,^21^ our study is the first to confirm agonist activity for compound **1** and to report EC_50_ values for compounds **2–4**. Among these, compounds **1–**-**3** showed similar agonist potency, whereas compound **4** exhibited 3- to 6-fold reduced sensitivity at α9α10 receptors. Based on IC_50_ values relative to compound **1**, compounds **2**, **3**, **5–7**, **21**, and **22** were more potent inhibitors (IC_50_ ratio <1.0), followed by compounds **4**, **8–10**, and **14–20** (IC_50_ ratio 1.0–5.0). Compounds **11–13** were the least potent (IC_50_ values >300 nM, ratio >5.0). A similar ranking emerged for agonist potency (EC_50_): compounds **2**, **3**, **5–8**, and **21** were more potent than compound **1** (EC_50_ ratio <1.0), while compounds **4**, **9**, **10, 12,** and **14–19** had moderate potency (EC_50_ ratio between 1.0 and 5.0), and compound **13** was least potent (EC_50_ > 5 μM, ratio >5.0).

At hα9 nAChRs, compounds **1**, **2**, **16**, and **21** also showed both agonist and antagonist activity, with EC_50_ values four to seven times higher than corresponding IC_50_ values. Compound **22** again acted solely as an antagonist. All antagonists were more potent than compound **1** (IC_50_ ratio <1.0), and only compound **2** showed higher agonist potency at hα9 receptors (EC_50_ ratio <1.0).

Compounds with both agonist and antagonist activity (e.g., **1** and **2**) behaved as agonists when applied alone but functioned as potent antagonists in the presence of ACh. This bidirectional action suggests state-dependent or allosteric modulation, which could be therapeutically valuable in conditions such as pain and inflammation where α9-containing nAChRs are upregulated. For example, compound **2** showed an IC_50_ of 39.1 nM for antagonism and an EC_50_ of 272.7 nM for agonism, indicating preferential stabilization of a nonconducting, antagonist-bound state.

### Mechanistic Insights from Molecular Dynamics Simulations

Intriguingly, compounds **21** and **22** differ only in the position of an oxygen atom, although both experiments and MD simulation highlight a different behavior for the two molecules. Inspection of the docked structures of both compounds highlight one possible key role of this positional difference (Figure S1A–B). The position of the benzyloxy or phenoxymethyl oxygen subtly alters the initial interaction between the ligand and the canonical binding site of hα9. In compound **21**, the benzyloxy oxygen is positioned to form a direct hydrogen bond with hα9­(+)-Tyr192 (Figure S1A). In compound **22**, no such hydrogen bond is predicted, but instead, an aromatic π–π stacking interaction is formed with hα9­(+)-Tyr199 (Figure S1B). This “switch” in preferential interatomic contact between Tyr192 or Tyr199 may be a key driver for the subsequent variation in dynamical loop C behavior induced by contact with compound **21** or **22**, respectively.

MD simulations revealed that the minor structural difference also appears to affect the interaction of secondary ligands which bind at allosteric sites. For compound **21**, at moderate concentration, the secondary ligand binds near the tip of loop C (Figure 6G). Inspection of the 2D ligand–receptor interaction diagram (Figure S1C) shows that the benzyloxy ring retains a π-π stacking interaction with Tyr192, thus forming a *direct* connection with hα9, which may help stabilize the loop into a well-defined closure. For compound **22**, in contrast, rather than bind close to the loop C tip, at moderate concentration, the ligands either do not bind close to each other, or else form tightly bound aggregates which insert as dimers into the loop C pocket (Figure 6M). As a result, the secondary compound **22** ligand does not form substantial *direct* contacts with hα9 (Figure S1D) and may therefore be less capable of locking loop C into a relatively rigid closure distance of 8–9 Å as observed for compound **21**. Given that interaction with hα9-Tyr192 appears to be a key point of difference between compound **21** and compound **22**, future work involving mutagenesis of this residue may serve to determine its role in modulating the concentration-dependent agonism or antagonism of these and related compounds.

MD flooding simulations of **21** at α9 nAChRs support an allosteric mechanism underlying its dual activity. At low concentrations, compound **21** adopted a “sideways” orientation in the orthosteric site, preventing loop C closure (>12 Å), consistent with classical antagonism. At higher concentrations, however, the compound reoriented parallel to the ECD’s long axis, promoting loop closure (8–10 Å) and agonist-like activation.

We propose that compound **21** concurrently engages both the orthosteric site and an allosteric region near the conserved Cys-Cys motif, stabilizing receptor conformations resembling those induced by classical agonists. In contrast, compound **22** induced both much wider (>12.3 Å) and tighter loop C closure (∼6–8 Å). These conformations are associated, respectively, with antagonist-bound AChBP (e.g., PDB: 4ALX) or desensitized α4β2 nAChR structures (PDB: 5KXI). This may explain its lack of agonist activity despite potent antagonism. The greater diversity of loop C distances induced by compound **22** may partly explain the relative antagonism (or at least lack of agonism) of this molecule against hα9 compared to compound **21**, the latter promoting more rigid loop C distances more representative of agonist-bound nAChR. Thus, distinct conformational effects likely underlie the divergent pharmacology of compounds **21** and **22**.

### Activity at hα7 nAChRs

In contrast to their effects at α9-containing receptors, compounds **1**, **2**, **16**, **21,** and **22** exhibited relatively weaker and exclusively antagonistic activity at hα7 nAChRs. Compared to compound **1**, the other compounds showed no significant differences in potency as hα7 antagonists. Compound **1** was first reported to inhibit ACh-evoked currents (100 μM ACh) mediated by chick α7 nAChRs expressed in *X. laevis* oocytes, with IC_50_ values in the range of 90–110 nM.^36,37^ More recent studies have characterized it as an antagonist at hα7, reporting an IC_50_ of 41 nM with 200 μM ACh^34^ and 1.99 μM with 1 mM ACh.^21^ In comparison, the IC_50_ value of 433 nM determined in the present study (using 100 μM ACh near the EC_50_ for ACh at hα7) falls approximately 10-fold higher and about 5-fold lower, respectively. These differences likely reflect the influence of ACh concentration on measured potency, consistent with competitive antagonism. In agreement with previous findings, compound **1** did not evoke currents when applied alone to wild-type chick α7 nAChRs but did activate the ACh-sensitive α7­[L247T] mutant receptor, with an EC_50_ of 0.2 nM.^37^

Compound **2** was reported previously to lack antagonistic activity at hα7 nAChRs in the presence of 1 mM ACh,^21^ contrasting with the IC_50_ value of 520 nM observed in this study using 100 μM ACh (near the EC_50_ at hα7 receptors). This discrepancy likely reflects competitive antagonism, where the high concentration of ACh in the earlier study was sufficient to overcome the inhibitory effect of compound **2**.

### Activity and Selectivity across nAChR Subtypes

Analysis of the activity and selectivity profiles across the nAChR subtypes, limited to the five compounds (**1**, **2**, **16**, **21**, and **22**) tested at the α9α10, α9, and α7 nAChRs, reveals several noteworthy trends. All five compounds exhibited consistent antagonistic potency at α7 nAChRs with IC_50_ values around 400 nM. In contrast, their antagonistic potency at α9-containing nAChRs increased progressively across the series. Compounds **1** and **16** showed IC_50_ values near 100 nM, while compounds **2** and **21** demonstrated greater potency, and compound **22** was the most potent, with IC_50_ values around 10 nM at both α9α10 and α9 receptors. This progression reflects an approximate 20-fold selectivity for α9-containing receptors over α7 nAChRs in the most potent compounds.

Within the five compounds, the trend of the agonist activities at the three receptor subtypes is somehow consistent with the trend of the antagonist activities. The α7 agonism profile is flat like the α7 antagonism profile: all the five compounds are devoid of agonist activity. Conversely, the profile of the agonist activity at α9-containing nAChRs is not uniform. Compounds **1**, **2**, **16**, and **21** show submicromolar agonist activity at α9-containing nAChRs with values of EC_50_ three-eight times higher than the corresponding IC_50_ values, which hover around 100 nM. Within the five compounds, **22** stood out as unique. It combined potent and selective antagonism at α9-containing receptors (IC_50_ values of 16 nM at α9α10 and 26 nM at α9) with no detectable agonist activity at any receptor subtype tested, including α7. This distinguishes compound **22** from both agonist-antagonist dual-activity compounds and pure antagonists with lower potency. This unique profile of compound **22** is further emphasized when compared to the broader set of 22 compounds tested at α9α10 nAChRs. For example, compounds **3** and **7** matched the antagonistic potency of **22** but retained moderate agonist activity, while compounds **11** and **20**, like **22**, lacked agonist activity but showed much weaker antagonism. Thus, compound **22** is distinctive in pairing high antagonist potency with complete absence of agonism.

Among all tested compounds, compound **3** emerged as the most potent antagonist at α9α10 nAChRs while also exhibiting moderate agonist potency, positioning it as a particularly promising lead candidate for further development. In contrast, compound **11** functioned solely as an antagonist and lacked agonist activity even at high concentrations. This profile may reflect an orthosteric blocking mechanism or stabilization of a nonconducting receptor.

### Structure–Activity Relationships

Specific structural modifications to compound **1**, at the ammonium head, the O–N linker, and the vinylene bridge of **1**, resulting in compounds **2**, **3**, and **4**, respectively, have been previously shown to significantly alter nicotinic receptor affinity and activity profiles. Compared to **1**, compounds **2**, **3**, and **4** showed markedly reduced binding affinity for α7 nAChRs and acted as antagonists at α9α10 receptors (when coapplied with 1 mM ACh), but not at α7 receptors.^21,23^ At high concentrations, compounds **2–4** also behaved as partial agonists at α9α10 nAChRs, with negligible or no agonism at α7 nAChR. The current study confirms and extends these findings. Both compounds **1** and **2** are antagonists at α7 and α9-containing nAChRs, but compound **2** displays greater potency and selectivity for α9α10 and α9 subtypes compared to **1**. Neither compound exhibits agonist activity at α7, whereas both activate α9-containing nAChRs, with compound **2** showing slightly greater agonist potency.

To further explore structure–activity relationships, we systematically evaluated the ability of compounds **2–4** and a library of compounds built around **2–4** to modulate ACh-evoked responses at α9α10 receptors. The derivatives forming the library incorporate and combine modifications to compound **1** in line with those converting **1** into **2** and **4** and already proved to orient the activity profile to α9-containing nAChRs. A key structural focus was the steric enlargement of the ammonium head through the introduction of one or two alicyclic or aromatic substituents (compounds **5–13**). Results from IC_50_ and EC_50_ assays at α9α10 nAChRs demonstrated that replacement of one of the ethyl residues of **1** with 1-adamantanyl (compound **5**), 4-tetrahydropyranyl (**6**) or cyclopentyl (**7**) preserved or modestly improved both antagonist and agonist potency compared to compound **2** (IC_50_ and EC_50_ ratios <1). In contrast, replacement with bulkier or aromatic groups, cycloheptyl (**8**), phenyl (**9**), or benzyl (**10**), resulted in marked loss of potency to both compounds **1** and **2**. This trend was further exacerbated with double replacement of ethyl residues with alicyclic or aromatic substituents, both with alicycles (**11** and **12**) and aromatic groups (**13**). Notably, the dicyclohexyl derivative (**11**) had modest activity as antagonist and no activity as agonist at the α9α10 nAChR. These findings highlight that a single, nonaromatic, moderately sized monocyclic substituent at the ammonium head favors activity at α9-containing receptors, whereas larger or aromatic groups, especially double substitutions, compromise receptor engagement.

Modifications to other regions of the scaffold produced similarly instructive outcomes. Elongation of the O–N ethylene linker in compound **2**, as in compounds **14** and **15**, significantly reduces both antagonism and agonism, likely compromising the optimal spatial alignment of the cyclohexyldimethylammonium head and/or the terminal phenyl group within the binding site. Similarly, saturation of the vinylene bridge (compound **16**), which removes rigidity from the stilbene scaffold, also reduced activity. These results highlight the importance of maintaining the relative orientation of the ammonium head and aromatic tail for effective receptor binding. Further insights were obtained by modifying the terminal styryl group of compound **2** and its triethylammonium analogue **1** with benzyloxy or phenoxymethyl moieties (compounds **21** and **22** for the 2-series; **19** and **20** for the 1-series). In the **1**-series, these modifications reduced α9α10 antagonist potency. In contrast, the **2**-series analogues retained high antagonist potency, with IC_50_ values in the low ten-nanomolar range. Regarding agonist activity, the benzyloxy substitution preserved α9α10 agonism in both series, whereas the phenoxymethyl group abolished it entirely.

Among all compounds tested across the three receptor subtypes, the three most impactful modifications of compound **1** were (1) the introduction of the cyclohexyldimethylammonium head (compound **2**), and its further combination with (2) a benzyloxy group (compound **21**) or (3) a phenoxymethyl group (compound **22**). Compounds **2** and **21** emerged as potent and selective antagonists at α9-containing nAChRs, with no agonist activity at α7 and moderate agonism at α9-containing subtypes. Compound **22** was notable in that it retained high antagonist potency and selectivity for α9α10 and α9 nAChRs, while completely lacking agonist activity across all tested subtypes.

### Physiological and Therapeutic Implications

Given the growing interest in nAChRs containing subunit α9 as therapeutic targets for conditions such as auditory neuropathy, chronic pain, inflammation, and cancer,^38^ the discovery of highly potent and selective modulators is of considerable translational relevance. However, despite compelling evidence for the druggability of these receptor subtypes, it remains unclear whether therapeutic benefit is best achieved through agonism or antagonism. This primarily reflects the incomplete understanding of the function and signaling mechanism of metabotropic NCNRs in modulating, in particular, inflammatory disease and neuropathic pain. Additional complexity arises from discrepancies between the pharmacological activity profiles at the ionotropic receptors, their corresponding metabotropic forms in cell-based assays, and the outcomes of in vivo studies. Within this context, the selectivity and dual activity profiles (agonist/antagonist or pure antagonist) exhibited by our compounds at the ionotropic α9-containing nAChR provide an opportunity to explore their modulatory potential at metabotropic NCNRs involved in inflammation.

We evaluated the effects of compounds **1**, **2**, **16**, **21**, and **22** on the BzATP-triggered release of the pro-inflammatory cytokine IL-1β by human monocytic THP-1 cells. As described previously,^14,15^ the classical nAChR agonist ACh efficiently inhibited the BzATP-induced release of IL-1β. At low concentrations (100 pM), all five compounds reversed the inhibitory action of ACh and showed minimal effects on the BzATP-induced IL-1β release by human monocytic THP-1 cells. This finding is consistent with their antagonistic activity at ionotropic α9α10 and α9 receptors, suggesting that they also function as NCNR antagonists at low concentrations. Of note, the concentration needed to antagonize the effect of ACh is in the pM range, far below the IC_50_ values measured for the conventional ionotropic function, which might be of advantage, when aiming at their potential therapeutic use. At a higher concentration (100 nM), compound **1** continued to partially antagonize the inhibitory effect of ACh, while at best minor effects were seen with all other compounds investigated.

When applied at a concentration of 100 pM in the absence of ACh, all compounds exerted at best minor inhibitory effects on the BzATP-induced release of IL-1β, which is in line with their antagonist properties. In contrast, high concentrations (100 nM) of compounds **2**, **16**, **21**, and **22** efficiently inhibited IL-1β release, suggesting an agonistic function at NCNRs.

For compounds **2**, **16**, and **21**, each exhibiting dual activity (agonism and antagonism) at α9-containing nAChRs, this suggests a concentration-dependent switch to agonist-like behavior at NCNRs. Compound **22**, however, lacks detectable agonist activity at ionotropic receptors yet still produced strong inhibition of IL-1β release. This finding implies that mechanism of inhibition induced by compound **22** may involve direct inhibition of the NLRP3 inflammasome or engagement of alternative targets or pathways in monocytes that bypass conventional NCNR signaling.

Our results support the notion that compounds **2**, **16**, and **21** retain their dual agonist/antagonist behavior at metabotropic NCNRs, acting as antagonists at low concentrations and as agonists at higher concentrations. However, this parallelism does not extend to compounds **1** and **22**. Compound **1**, despite an ionotropic profile similar to **16** (including comparable agonist potency at α9-containing receptors), failed to reduce IL-1β release at 100 nM. Conversely, compound **22**, which lacks agonist activity entirely, still elicited strong anti-inflammatory effects at high concentrations, indicating an NCNR-independent or noncanonical mechanism.

Overall, our cell-based data demonstrate that compounds **2**, **16**, **21**, and **22** can potently mediate anti-inflammatory activity. However, consistency of such an activity with their profiles of nicotinic antagonists and/or agonists remains partly ambiguous. Given the complexity of CAS, more experiments in vitro and in vivo will be needed to elucidate the mechanism of the demonstrated interference by these compounds with inflammatory processes and the involvement of monocytic NCNRs. Nevertheless, compound **2** and its analogues **16**, **21**, and **22** provide a valuable benchmark for further pharmacological profiling in therapeutic models of inflammation and pain.

## Conclusions

In summary, this study identified and pharmacologically characterized a panel of small molecules for their ionotropic activity at α9α10 nAChRs. Several compounds emerged as potent and full antagonists with IC_50_ values ranging from 10 to 100 nM. Notably, most of these compounds exhibited full agonist activity at higher submicromolar concentration, while a minority lacked agonist function altogether.

Further pharmacological characterization of selected compounds revealed that potent α9α10 antagonism extended to homomeric α9 nAChRs and was consistently selective over α7 nAChRs. Similarly, for most compounds, α9α10 agonist activity also extended to α9 nAChRs, again with complete selectivity over α7. The discovery of molecules with such pronounced selectivity and efficacy for α9 and α9α10 receptors, relative to α7, was enabled by the further pharmacological profiling of the cyclohexyldimethylammonium ethyl ether of 4-stilbenol (compound **2**), a known selective α9α10 antagonist, and systematic refinement of its chemical scaffold. The broader pharmacological characterization confirmed the selectivity of compound **2** for the α9-containing nAChRs and revealed a dual, “Janus-like” profile: functioning as a full antagonist at low concentrations and as a full agonist at higher concentrations, with clearly defined IC_50_ and EC_50_ values. Modification of the compound **2** scaffold demonstrated that certain changes to the terminal styryl portion can retain or even enhance this pharmacological profile. Substitution with a benzyloxy group (compound **21**) preserved the dual-activity and selectivity of compound **2**. In contrast, replacement with a phenoxymethyl group (compound **22**), markedly increased the potency and selectivity of antagonism at α9-containing nAChRs while abolishing all agonist activity across the tested receptor subtypes.

The dual α9* antagonist/agonist profile of **21** was tentatively elucidated conducting MD simulations. These support a concentration-dependent, allosteric mechanism of **21**: sideway occupation of the orthosteric site precluding, consistently with antagonism, loop C closure at low concentration and, with increasing concentration, parallel reorienting inside the orthosteric site and concurrent engagement of an allosteric region promoting, consistently with agonism, loop C closure.

New pharmacological agents discriminating between α9-containing and α7 nAChRs, such as **2**, **21**, and **22**, are invaluable tools for probing the functions of α9 and α9α10 nAChRs, as metabotropic NCNRs, in neuropathic and inflammatory pain and potential therapeutics to treat these pathologies avoiding not only addiction by opioid analgesics. Furthermore, CNS side effects commonly linked to α7 modulation are unlikely, when targeting α9-containing nAChRs, because subunit α9 is exclusively expressed in the periphery. Interference with the ionotropic function of α9* nAChR in the auditory system may be circumvented using compounds of this substance group, as they were efficient at much lower concentrations at the NCNRs of monocytic THP-1 cells compared to conventional ionotropic receptors. Moreover, both α7 and α9* receptors are potential targets in CAS. In particular, recent studies in vivo utilizing α7 nAChR knockout animals strongly support the α9* nAChR-dependent mechanism of CAS.^39^ Whether CAS activity is associated to α9* nAChR activation or inactivation remains matter of ongoing debate.

Our cytokine-release assays show that **2**, **21**, and **22** potently inhibit ATP-mediated release of pro-inflammatory IL-1β at 100 nM concentration, suggesting that they hold therapeutic promise for treatment of inflammation and for pain management. As for the underlying mechanism of action, involvement of modulation of nAChRs in this in vitro activity is indicated by the observed reversion, at lower concentrations, of the CAS activity of ACh by all the three compounds. Such a reversion would match with their antagonist activity profiles at ionotropic nAChRs. The relationship between inhibition of IL-1β release and agonist activity at ionotropic α9* nAChRs is instead ambiguous as **22**, unlike **2** and **21**, completely lacks any ionotropic agonist activity at α9* nAChRs, nevertheless maintaining the inhibition activity of **2** and **21**. Interestingly, the behavior of **22** is reminiscent of that of nicotine. In fact, nicotine, which does not evoke ion currents responses in α9* nAChRs but antagonizes their ionotropic functions, inhibits IL-1β release in monocytes and triggers anti-inflammatory mechanisms metabotropically acting at α9* nAChRs.^40^ Overall, these observations stress that metabotropic effects at NCNRs cannot be univocally predicted and explained in the light of the activity profiles at ionotropic nAChRs, although defining the ionotropic profiles is the necessary premise of any further, more detailed pharmacological characterization.

## Experimental Section

### Chemistry

All chemicals and solvents were used as received from commercial sources or prepared, as described in the literature. Flash chromatography purifications were performed using Sfaṙ Silica D 60 μm cartridges. Thin-layer chromatography (TLC) analyses were carried out on alumina sheets precoated with silica gel 60 F254. *R*_f_ values are given for guidance. ^1^H NMR spectra were recorded at 300 MHz, while ^13^C NMR spectra were recorded at 75 MHz using FT-NMR spectrometers. Chemical shifts are reported in ppm relative to residual solvent as the internal standard. Melting points were determined by a Buchi Melting Point B-540 apparatus. Mass Spectra (MS) were acquired by direct infusion on a AB Sciex 3200 QTRAP (AB Sciex, Concord, ON, Canada) equipped with electrospray ionization TurboIonSpray source operating in positive mode (ESI^+^). Purity was measured by analytical high-performance liquid chromatography (HPLC) on a Hewlett-Packard serie 1050 UV–vis HPLC using a VisionHT C18 Classic column (particle size 5 μm, 250 × 4.6 mm); isocratic elution MeCN/H_2_O/TFA (90:10:0.1) over 13 min. All compounds are >95% pure by HPLC analysis and retention times (*R*_t_) are **1**–**5**, **11**, **13**, **17**–**20**, **23**, **24**, **29**, **32**, **34**, **37**, **39**,^21,23,35^ and **43** ^41^ were synthesized as previously reported.

### Method A

#### General Procedure for the Synthesis of Compounds **6**–**9**, **12**, **14**–**16**, **21**, and **22**

The appropriate tertiary amine (1 equiv) was dissolved in dichloromethane (0.1 M). Iodomethane (50 equiv) was added, and the reaction mixture was stirred 16 h at rt, unless otherwise specified. Et_2_O was added and stirred for 10 min. The solid was isolated by filtration, washed with Et_2_O and hexane and purified as specified to give the desired products.

### Method B

#### General Procedure for the Synthesis of Compounds **40** and **44**

The appropriate phenol derivative (1 equiv) was dissolved in acetone (0.5 M), followed by the addition of K_2_CO_3_ (2.5 equiv). After 20 min of stirring, 1,2-dibromoethane (4.2 equiv) was added and the reaction mixture was stirred at reflux overnight, unless otherwise specified. After cooling, the solvent was evaporated under reduced pressure and the residue was taken in H_2_O and EtOAc. The phases were separated, and the water phase was extracted twice with EtOAc. The combined organic phase was washed with brine, dried over Na_2_SO_4_, and concentrated under reduced pressure. Purification of resultant residue by flash chromatography on silica gel (gradient of cyclohexane/EtOAc from 0 to 15% EtOAc) afforded the desired products.

### Method C

#### General Procedure for the Synthesis of Compounds **31** and **47**

Under a nitrogen atmosphere, the appropriate phenol derivative (1 equiv) was dissolved in *anhydrous* THF (0.3–0.8 M). PPh_3_ (1.2–1.25 equiv) and an appropriate alcohol derivative (1–1.25 equiv) were added at rt, followed by addition of DIAD (1.2–1.25 equiv) at 0 °C. The reaction mixture was then stirred at rt overnight and purified as specified affording desired products.

### Method D

#### General Procedure for the Synthesis of Compounds **33**, **41**, and **45**

The appropriate alkyl halide (1 mmol) was dissolved in a saturated solution of NaI in acetone (7 mL) and the reaction mixture was refluxed overnight. The solvent was evaporated. The residue was taken in EtOAc and 10% solution of Na_2_S_2_O_5_. The phases were separated, and the water phase was further extracted with EtOAc. The combined organic phase was washed with brine, dried over Na_2_SO_4_ and evaporated affording the desired products.

### Method E

#### General Procedure for the Synthesis of Compounds **26**–**28**, **30**, **35**, **36**, **42**, and **46**

The appropriate alkyl iodide (1 equiv) was dissolved in toluene (0.2 M), followed by the addition of the appropriate secondary amine (4–20 equiv). The reaction mixture was stirred at 65 °C overnight. The reaction mixture was taken in CH_2_Cl_2_ and washed twice with 1 M NaOH, and once with brine. The organic phase was dried over anhydrous Na_2_SO_4_ and the solvent was evaporated. The residue was purified by flash chromatography on silica gel (gradient of CH_2_Cl_2_/MeOH from 0 to 10% MeOH) yielding the desired products.

#### Synthesis of (E)-*N*,*N*-Dimethyl-*N*-(2-(4-styrylphenoxy)­ethyl)­tetrahydro-2*H*-pyran-4-aminium Iodide (**6**)

Obtained from **26** (72 mg, 0.21 mmol), iodomethane (664 μL, 10.67 mmol) in CH_2_Cl_2_ (2 mL), following the general METHOD A. Recrystallization from MeOH/Et_2_O afforded compound **6** as pale yellow solid in 80% yield. mp = 195–197 °C. *R*_t_ (HPLC) = 6.616 min. MS (ESI): *m*/*z* calcd for C_23_H_30_NO_2_ [M^+^] = 352.23, found 352.2. ^1^H NMR (300 MHz, DMSO-*d*_6_) δ 7.64–7.53 (m, 4H), 7.42–7.31 (m, 2H), 7.29–7.18 (m, 2H), 7.13 (d, *J* = 16.5 Hz, 1H), 7.02 (d, *J* = 8.8 Hz, 2H), 4.56–4.43 (m, 2H), 4.05 (dd, *J* = 11.4, 4.3 Hz, 2H), 3.88–3.73 (m, 3H), 3.40–3.28 (m, 2H), 3.09 (s, 6H), 2.11–1.99 (m, 2H), 1.78 (qd, *J* = 11.8, 4.4 Hz, 2H). ^13^C NMR (75 MHz, DMSO-*d*_6_) δ 157.0, 137.2, 130.5, 128.7, 127.8, 127.3, 126.6, 126.2, 114.9, 69.2, 65.7, 61.4, 60.6, 48.0, 26.2.

#### Synthesis of (E)-*N*,*N*-Dimethyl-N-(2-(4-styrylphenoxy)­ethyl)­cyclopentanaminium Iodide (**7**)

Obtained from **27** (100 mg, 0.31 mmol), iodomethane (968 μL, 15.55 mmol) in CH_2_Cl_2_ (3 mL), following the general METHOD A. Trituration with hexane gave **7** as white solid in 82% yield. mp = 220–222 °C (dec). *R*_t_ (HPLC) = 6.290 min. MS (ESI): *m*/*z* calcd for C_23_H_30_NO [M^+^] = 336.23, found 336.2. ^1^H NMR (300 MHz, DMSO-*d*_6_) δ 7.65–7.54 (m, 4H), 7.42–7.31 (m, 2H), 7.29–7.18 (m, 2H), 7.13 (d, *J* = 16.5 Hz, 1H), 7.03 (d, *J* = 8.7 Hz, 2H), 4.50 (t, *J* = 4.8 Hz, 2H), 4.07 (p, *J* = 8.2 Hz, 1H), 3.76 (t, *J* = 4.8 Hz, 2H), 3.09 (s, 6H), 2.06–1.83 (m, 4H), 1.79–1.47 (m, 4H). ^13^C NMR (75 MHz, DMSO-*d*_6_) δ 157.0, 137.2, 130.5, 128.7, 127.8, 127.3, 126.6, 126.2, 114.9, 74.4, 62.1, 61.4, 48.2, 25.6, 23.9.

#### Synthesis of (E)-*N*,*N*-Dimethyl-*N*-(2-(4-styrylphenoxy)­ethyl)­cycloheptanaminium Iodide (**8**)

Obtained from **28** (83 mg, 0.24 mmol), iodomethane (739 μL, 11.87 mmol) in CH_2_Cl_2_ (2 mL), following the general METHOD A. Recrystallization from EtOH/Et_2_O afforded compound **8** as white solid in 72% yield. mp = 222–223 °C. *R*_t_ (HPLC) = 6.608 min. MS (ESI): *m*/*z* calcd for C_25_H_34_NO [M^+^] = 364.26, found 364.4. ^1^H NMR (300 MHz, DMSO-*d*_6_) δ 7.66–7.52 (m, 4H), 7.41–7.32 (m, 2H), 7.28–7.18 (m, 2H), 7.13 (d, *J* = 16.5 Hz, 1H), 7.03 (d, *J* = 8.7 Hz, 2H), 4.48 (t, *J* = 4.7 Hz, 2H), 3.82 (t, *J* = 4.7 Hz, 2H), 3.73–3.60 (m, 1H), 3.10 (s, 6H), 2.26–2.10 (m, 2H), 1.85–1.63 (m, 4H), 1.61–1.43 (m, 6H). ^13^C NMR (75 MHz, DMSO-*d*_6_) δ 157.0, 137.2, 130.5, 128.6, 127.8, 127.3, 126.6, 126.2, 114.9, 73.6, 61.5, 60.7, 48.3, 26.8, 26.4, 24.3.

#### Synthesis of (E)-*N*,*N*-Dimethyl-*N*-(2-(4-styrylphenoxy)­ethyl)­benzenaminium Iodide (**9**)

Obtained from **25** (81 mg, 0.25 mmol), iodomethane (765 μL, 12.29 mmol) in CH_2_Cl_2_ (2 mL) at reflux 36 h, following the general METHOD A. Compound **9** was obtained as white solid in 71% yield. mp = 157–158 °C. *R*_t_ (HPLC) = 5.798 min. MS (ESI): *m*/*z* calcd for C_24_H_26_NO [M^+^] = 344.20, found 344.2. ^1^H NMR (300 MHz, DMSO-*d*_6_) δ 8.01–7.91 (m, 2H), 7.69–7.58 (m, 3H), 7.57–7.52 (m, 2H), 7.49 (d, *J* = 8.8 Hz, 2H), 7.40–7.30 (m, 2H), 7.27–7.21 (m, 1H), 7.17 (d, *J* = 16.5 Hz, 1H), 7.08 (d, *J* = 16.5 Hz, 1H), 6.76 (d, *J* = 8.8 Hz, 2H), 4.38 (t, *J* = 4.6 Hz, 2H), 4.16 (t, *J* = 4.6 Hz, 2H), 3.73 (s, 6H). ^13^C NMR (75 MHz, DMSO-*d*_6_) δ 156.7, 144.8, 137.2, 130.5, 130.1, 130.0, 128.6, 127.7, 127.7, 127.3, 126.6, 126.2, 121.3, 114.7, 114.6, 67.1, 62.0, 54.8, 54.7.

#### Synthesis of (E)-*N*-Benzyl-*N*,*N*-dimethyl-2-(4-styrylphenoxy)­ethanaminium Bromide (**10**)

**29** (87 mg, 0.32 mmol) was dissolved in THF (2 mL). Benzyl bromide (194 μL, 1.63 mmol) was added, and the reaction mixture was refluxed 16 h. The mixture was cooled at rt, Et_2_O was added and stirred for 10 min. The solid was isolated by vacuum filtration, washed with Et_2_O and hexane to give compound **10** as white solid in 91% yield. mp = 182–184 °C. *R*_t_ (HPLC) = 5.580 min. MS (ESI): *m*/*z* calcd for C_25_H_28_NO [M^+^] = 358.22, found 358.4. ^1^H NMR (300 MHz, DMSO-*d*_6_) δ 7.68–7.48 (m, 9H), 7.42–7.31 (m, 2H), 7.28–7.19 (m, 2H), 7.13 (d, *J* = 16.5 Hz, 1H), 7.05 (d, *J* = 8.8 Hz, 2H), 4.69 (s, 2H), 4.58 (t, *J* = 4.7 Hz, 2H), 3.79 (t, *J* = 4.7 Hz, 2H), 3.09 (s, 6H). ^13^C NMR (75 MHz, DMSO-*d*_6_) δ 157.1, 137.2, 133.2, 130.5, 130.3, 128.9, 128.7, 128.0, 127.83, 127.81, 127.3, 126.6, 126.2, 115.0, 67.2, 62.5, 61.5, 49.8.

#### Synthesis of (E)-*N*-Cyclopentyl-*N*-methyl-N-(2-(4-styrylphenoxy)­ethyl)­cyclopentanaminium Iodide (**12**)

Obtained from **30** (65 mg, 0.17 mmol), iodomethane (539 μL, 8.65 mmol) in CH_2_Cl_2_ (1.5 mL), following the general METHOD A. Recrystallization from MeOH/Et_2_O afforded compound **12** as white solid in 78% yield. mp = 173–174 °C. *R*_t_ (HPLC) = 6.397 min. MS (ESI): *m*/*z* calcd for C_27_H_36_NO [M^+^] = 390.28, found 390.1. ^1^H NMR (300 MHz, DMSO-*d*_6_) δ 7.64–7.51 (m, 4H), 7.41–7.31 (m, 2H), 7.29–7.17 (m, 2H), 7.12 (d, *J* = 16.5 Hz, 1H), 7.03 (d, *J* = 8.3 Hz, 2H), 4.46 (t, *J* = 4.9 Hz, 2H), 4.07 (p, *J* = 8.5 Hz, 2H), 3.79 (t, *J* = 4.9 Hz, 2H), 3.03 (s, 3H), 2.10–1.83 (m, 8H), 1.83–1.47 (m, 8H). ^13^C NMR (75 MHz, DMSO-*d*_6_) δ 156.9, 137.2, 130.6, 128.7, 127.8, 127.8, 127.3, 126.6, 126.2, 114.9, 74.7, 61.9, 58.3, 42.3, 26.1, 25.9, 23.1, 23.0.

#### Synthesis of (E)-*N*,*N*-Dimethyl-*N*-(3-(4-styrylphenoxy)­propyl)­cyclohexanaminium Iodide (**14**)

Obtained from **35** (70 mg, 0.20 mmol), iodomethane (623 μL, 10.01 mmol) in CH_2_Cl_2_ (2 mL), following the general METHOD A. Recrystallization from EtOH afforded compound **14** as white solid in 62% yield. mp = 206–207 °C. *R*_t_ (HPLC) = 6.682 min. MS (ESI): *m*/*z* calcd for C_25_H_34_NO [M^+^] = 364.26, found 364.3. ^1^H NMR (300 MHz, DMSO-*d*_6_) δ 7.62–7.51 (m, 4H), 7.41–7.30 (m, 2H), 7.29–7.17 (m, 2H), 7.11 (d, *J* = 16.5 Hz, 1H), 6.97 (d, *J* = 8.7 Hz, 2H), 4.09 (t, *J* = 5.9 Hz, 2H), 3.53–3.44 (m, 2H), 3.44–3.34 (m, 1H), 3.01 (s, 6H), 2.24–2.02 (m, 4H), 1.92–1.79 (m, 2H), 1.66–1.54 (m, 1H), 1.54–1.21 (m, 4H), 1.20–1.03 (m, 1H). ^13^C NMR (75 MHz, DMSO-*d*_6_) δ 157.8, 137.3, 130.0, 128.6, 127.9, 127.8, 127.2, 126.3, 126.1, 114.8, 70.7, 64.7, 59.2, 47.8, 25.3, 24.8, 24.3, 22.0.

#### Synthesis of (E)-*N*,*N*-Dimethyl-*N*-(4-(4-styrylphenoxy)­butyl)­cyclohexanaminium Iodide (**15**)

Obtained from **36** (40 mg, 0.11 mmol), iodomethane (342 μL, 5.50 mmol) in CH_2_Cl_2_ (1 mL), following the general METHOD A. Recrystallization from EtOH afforded compound **15** as off-white solid in 60% yield. mp = 171–172 °C. *R*_t_ (HPLC) = 6.762 min. MS (ESI): *m*/*z* calcd for C_26_H_36_NO [M^+^] = 378.28, found 378.2. ^1^H NMR (300 MHz, CD_3_OD) δ 7.57–7.44 (m, 4H), 7.37–7.27 (m, 2H), 7.24–7.17 (m, 1H), 7.12 (d, *J* = 16.4 Hz, 1H), 7.02 (d, *J* = 16.4 Hz, 1H), 6.94 (d, *J* = 8.8 Hz, 2H), 4.11 (t, *J* = 5.7 Hz, 2H), 3.48–3.34 (m, 3H), 3.05 (s, 6H), 2.25–2.13 (m, 2H), 2.06–1.81 (m, 6H), 1.77–1.65 (m, 1H), 1.64–1.33 (m, 4H), 1.33–1.14 (m, 1H). ^13^C NMR (75 MHz, DMSO-*d*_6_) δ 158.1, 137.3, 129.7, 128.6, 128.0, 127.8, 127.2, 126.1, 114.7, 70.8, 66.6, 61.3, 47.7, 25.5, 25.3, 24.9, 24.3, 18.6.

#### Synthesis of *N*,*N*-Dimethyl-*N*-(2-(4-phenethylphenoxy)­ethyl)­cyclohexanaminium Iodide (**16**)

Obtained from **38** (55 mg, 0.16 mmol), iodomethane (507 μL, 8.15 mmol) in CH_2_Cl_2_ (1.5 mL), following the general METHOD A. Recrystallization from EtOH/Et_2_O afforded compound **16** as white solid in 61% yield. mp = 155–156 °C. *R*_t_ (HPLC) = 6.142 min. MS (ESI): *m*/*z* calcd for C_24_H_34_NO [M^+^] = 352.53, found 352.3. ^1^H NMR (300 MHz, DMSO-*d*_6_) δ 7.34–7.11 (m, 7H), 6.89 (d, *J* = 8.6 Hz, 2H), 4.39 (t, *J* = 4.8 Hz, 2H), 3.78 (t, *J* = 4.8 Hz, 2H), 3.55–3.42 (m, 1H), 3.06 (s, 6H), 2.84 (s, 4H), 2.23–2.10 (m, 2H), 1.95–1.82 (m, 2H), 1.69–1.56 (m, 1H), 1.56–1.23 (m, 4H), 1.23–1.09 (m, 1H). ^13^C NMR (75 MHz, DMSO-*d*_6_) δ 155.5, 141.4, 134.3, 129.4, 128.3, 128.2, 125.7, 114.3, 71.9, 61.3, 60.8, 48.3, 37.2, 36.0, 25.5, 24.9, 24.3.

#### Synthesis of *N*-(2-(4-(Benzyloxy)­phenoxy)­ethyl)-*N*,*N*-dimethylcyclohexanaminium Iodide (**21**)

Obtained from **42** (116 mg, 0.34 mmol), iodomethane (1.1 mL, 17.08 mmol) in CH_2_Cl_2_ (3 mL), following the general METHOD A. Recrystallization from EtOH/Et_2_O afforded compound **21** as white solid in 66% yield. mp = 178–182 °C. *R*_t_ (HPLC) = 5.762 min. MS (ESI): *m*/*z* calcd for C_23_H_32_NO_2_ [M^+^] = 354.24, found 354.2. ^1^H NMR (300 MHz, DMSO-*d*_6_) δ 7.49–7.25 (m, 5H), 6.99 (d, *J* = 9.3 Hz, 2H), 6.93 (d, *J* = 9.3 Hz, 2H), 5.06 (s, 2H), 4.37 (t, *J* = 4.7 Hz, 2H), 3.77 (t, *J* = 4.7 Hz, 2H), 3.49 (tt, *J* = 11.7, 3.1 Hz, 1H), 3.07 (s, 6H), 2.24–2.08 (m, 2H), 1.92–1.80 (m, 2H), 1.66–1.55 (m, 1H), 1.55–1.38 (m, 2H), 1.38–1.21 (m, 2H), 1.21–1.03 (m, 1H). ^13^C NMR (75 MHz, DMSO-*d*_6_) δ 152.9, 151.5, 137.2, 128.4, 127.7, 127.5, 115.8, 115.5, 72.0, 69.6, 61.8, 60.8, 48.3, 25.5, 24.9, 24.3.

#### Synthesis of *N*,*N*-Dimethyl-*N*-(2-(4-(phenoxymethyl)­phenoxy)­ethyl)­cyclohexanaminium Iodide (**22**)

Obtained from **47** (150 mg, 0.44 mmol), iodomethane (1.4 mL, 22.09 mmol) in CH_2_Cl_2_ (4 mL), following the general METHOD A. Recrystallization from EtOH/Et_2_O afforded compound **22** as white solid in 55% yield. mp = 136–140 °C. *R*_t_ (HPLC) = 5.801 min. MS (ESI): *m*/*z* calcd for C_23_H_32_NO_2_ [M^+^] = 354.24, found 354.3. ^1^H NMR (300 MHz, DMSO-*d*_6_) δ 7.42 (d, *J* = 8.6 Hz, 2H), 7.36–7.21 (m, 2H), 7.05–6.96 (m, 4H), 6.92 (td, *J* = 7.2, 1.1 Hz, 1H), 5.03 (s, 2H), 4.45 (t, *J* = 4.8 Hz, 2H), 3.81 (t, *J* = 4.8 Hz, 2H), 3.56–3.43 (m, 1H), 3.08 (s, 6H), 2.22–2.09 (m, 2H), 1.94–1.80 (m, 2H), 1.66–1.55 (m, 1H), 1.55–1.21 (m, 4H), 1.21–1.09 (m, 1H). ^13^C NMR (75 MHz, DMSO-*d*_6_) δ 158.2, 157.0, 129.9, 129.5, 129.4, 120.6, 114.8, 114.5, 72.0, 68.6, 61.4, 60.7, 48.3, 25.5, 24.9, 24.3.

#### Synthesis of (E)-*N*-Methyl-*N*-(2-(4-styrylphenoxy)­ethyl)­aniline (**25**)

**24** (200 mg, 0.57 mmol) was dissolved in CH_3_CN (4 mL). *N*-Methylaniline (217 μL, 2.00 mmol) and K_2_CO_3_ (276 mg, 2.00 mmol) were added and the reaction mixture was refluxed for 24 h. The reaction mixture was poured in H_2_O (20 mL) and extracted with EtOAc (2 × 20 mL). The combined organic phase was washed with brine (20 mL), dried over anhydrous Na_2_SO_4_ and concentrated in vacuo. Purification by flash chromatography on silica gel (gradient of petroleum ether/EtOAc from 0 to 5% EtOAc) followed by trituration with *n*-hexane (3 × 2 mL) gave **25** as white solid in 44% yield. mp = 124–127 °C. *R*_f_ = 0.45 (cHex/EtOAc 95:5). ^1^H NMR (300 MHz, CDCl_3_) δ 7.54–7.46 (m, 2H), 7.43 (d, *J* = 8.7 Hz, 2H), 7.39–7.31 (m, 2H), 7.31–7.22 (m, 3H), 7.06 (d, *J* = 16.3 Hz, 1H), 6.97 (d, *J* = 16.3 Hz, 1H), 6.87 (d, *J* = 8.7 Hz, 2H), 6.84–6.71 (m, 3H), 4.17 (t, *J* = 6.0 Hz, 2H), 3.77 (t, *J* = 6.0 Hz, 2H), 3.07 (s, 3H).

#### Synthesis of (E)-*N*-Methyl-N-(2-(4-styrylphenoxy)­ethyl)­tetrahydro-2H-pyran-4-amine (**26**)

Obtained from **24** (130 mg, 0.37 mmol) and *N*-methyl-*N*-tetrahydro-2H-pyran-4yl-amine (214 mg, 1.86 mmol) in toluene (2 mL), following the general METHOD E. Compound **26** was obtained as white solid in 80% yield. mp = 78–80 °C. *R*_f_ = 0.28 (CH_2_Cl_2_/MeOH 95:5). ^1^H NMR (300 MHz, CDCl_3_) δ 7.52–7.47 (m, 2H), 7.45 (d, *J* = 8.7 Hz, 2H), 7.39–7.30 (m, 2H), 7.26–7.20 (m, 1H), 7.07 (d, *J* = 16.3 Hz, 1H), 6.97 (d, *J* = 16.3 Hz, 1H), 6.90 (d, *J* = 8.7 Hz, 2H), 4.08 (t, *J* = 6.1 Hz, 2H), 4.06–4.00 (m, 2H), 3.39 (td, *J* = 11.6, 2.2 Hz, 2H), 2.91 (t, *J* = 6.1 Hz, 2H), 2.67 (tt, *J* = 11.4, 3.9 Hz, 1H), 2.41 (s, 3H), 1.81–1.71 (m, 2H), 1.70–1.54 (m, 2H).

#### Synthesis of (E)-*N*-Methyl-*N*-(2-(4-styrylphenoxy)­ethyl)­cyclopentanamine (**27**)

Obtained from **24** (130 mg, 0.37 mmol), *N*-methylcyclopentanamine (184 mg, 1.86 mmol) in toluene (2 mL), following the general METHOD E. Compound **27** was obtained as white solid in 98% yield. mp = 84–86 °C. *R*_f_ = 0.25 (CH_2_Cl_2_/MeOH 95:5). ^1^H NMR (300 MHz, CDCl_3_) δ 7.52–7.46 (m, 2H), 7.44 (d, *J* = 8.7 Hz, 2H), 7.39–7.30 (m, 2H), 7.26–7.19 (m, 1H), 7.07 (d, *J* = 16.3 Hz, 1H), 6.97 (d, *J* = 16.3 Hz, 1H), 6.90 (d, *J* = 8.7 Hz, 2H), 4.11 (t, *J* = 6.3 Hz, 2H), 2.87 (t, *J* = 6.3 Hz, 2H), 2.84–2.75 (m, 1H), 2.38 (s, 3H), 1.94–1.81 (m, 2H), 1.76–1.64 (m, 2H), 1.64–1.38 (m, 4H).

#### Synthesis of (E)-*N*-Methyl-*N*-(2-(4-styrylphenoxy)­ethyl)­cycloheptanamine (**28**)

Obtained from **24** (135 mg, 0.38 mmol), *N*-methylcycloheptanamine (265 mg, 2.08 mmol) in toluene (2 mL), following the general METHOD E. Compound **28** was obtained as off-white solid in 84% yield. mp = 75–77 °C. *R*_f_ = 0.19 (CH_2_Cl_2_/MeOH 95:5). ^1^H NMR (300 MHz, CDCl_3_) δ 7.51–7.46 (m, 2H), 7.44 (d, *J* = 8.8 Hz, 2H), 7.38–7.30 (m, 2H), 7.25–7.20 (m, 1H), 7.06 (d, *J* = 16.3 Hz, 1H), 6.97 (d, *J* = 16.3 Hz, 1H), 6.90 (d, *J* = 8.8 Hz, 2H), 4.09 (t, *J* = 6.2 Hz, 2H), 2.85 (t, *J* = 6.2 Hz, 2H), 2.77–2.64 (m, 1H), 2.38 (s, 3H), 1.97–1.83 (m, 2H), 1.80–1.38 (m, 10H).

#### Synthesis of (E)-*N*-Cyclopentyl-*N*-(2-(4-styrylphenoxy)­ethyl)­cyclopentanamine (**30**)

Dicyclopentylamine hydrochloride (120 mg, 0.78 mmol) was dissolved in water. The pH was corrected to 13–14 with 1 M NaOH and then extracted with dichloromethane (3 × 10 mL). The organic phase was washed with brine, dried over Na_2_SO_4_ and the solvent was evaporated affording dicyclopentylamine that was used in the following reaction. The title product was then synthesized from obtained dicyclopentylamine, and **24** (68.5 mg, 0.20 mmol) in toluene (1 mL), following the general METHOD E. Compound **30** was obtained as light brown solid in 99% yield. ^1^H NMR (300 MHz, CDCl_3_) δ 7.52–7.46 (m, 2H), 7.44 (d, *J* = 8.7 Hz, 2H), 7.39–7.29 (m, 2H), 7.26–7.19 (m, 1H), 7.06 (d, *J* = 16.3 Hz, 1H), 6.96 (d, *J* = 16.3 Hz, 1H), 6.88 (d, *J* = 8.7 Hz, 2H), 3.97 (t, *J* = 7.5 Hz, 2H), 3.29–3.10 (m, 2H), 2.84 (t, *J* = 7.5 Hz, 2H), 1.93–1.75 (m, 4H), 1.75–1.34 (m, 12H).

#### Synthesis of (E)-1-(3-Chloropropoxy)-4-styrylbenzene (**31**)

Obtained from (*E*)-4-hydroxystilbene (300 mg, 1.52 mmol), 3-chloro-1-propanol (160 μL, 1.91 mmol), PPh_3_ (501 mg, 1.91 mmol), DIAD (375 μL, 1.91 mmol) in *anhydrous* THF (2 mL), following the general METHOD C. The volatiles were evaporated, and the residue was purified by flash chromatography on silica gel (gradient of cyclohexane/EtOAc from 0 to 5% EtOAc) to give **31** as white solid in 58% yield. mp = 117–119 °C. *R*_f_ = 0.53 (cyclohexane/EtOAc 95:5). ^1^H NMR (300 MHz, CDCl_3_) δ 7.53–7.48 (m, 2H), 7.46 (d, *J* = 8.8 Hz, 2H), 7.39–7.30 (m, 2H), 7.26–7.20 (m, 1H), 7.07 (d, *J* = 16.3 Hz, 1H), 6.98 (d, *J* = 16.3 Hz, 1H), 6.91 (d, *J* = 8.8 Hz, 2H), 4.14 (t, *J* = 5.8 Hz, 2H), 3.76 (t, *J* = 6.3 Hz, 2H), 2.25 (p, *J* = 6.1 Hz, 2H).

#### Synthesis of (E)-1-(3-Iodopropoxy)-4-styrylbenzene (**33**)

Obtained from **31** (237 mg, 0.87 mmol) in a saturated solution of NaI in acetone (6 mL), following the general METHOD D. Compound **33** was obtained as white solid in 94% yield. mp = 119–121 °C (lit.^35^ 117 °C). *R*_f_ = 0.60 (cyclohexane/EtOAc 95:5).^1^H NMR was in accordance with previously published spectrum.^35^

#### Synthesis of (E)-*N*-Methyl-*N*-(3-(4-styrylphenoxy)­propyl)­cyclohexanamine (**35**)

Obtained from **33** (150 mg, 0.41 mmol), *N*-methylcyclohexanamine (1.1 mL, 8.24 mmol) in toluene (2 mL) following the general METHOD E. Compound **35** was obtained as yellow solid in 97% yield. mp = 77–78 °C. *R*_f_ = 0.10 (CH_2_Cl_2_/MeOH 95:5). ^1^H NMR (300 MHz, CDCl_3_) δ 7.52–7.46 (m, 2H), 7.44 (d, *J* = 8.7 Hz, 2H), 7.38–7.30 (m, 2H), 7.26–7.19 (m, 1H), 7.07 (d, *J* = 16.3 Hz, 1H), 6.96 (d, *J* = 16.3 Hz, 1H), 6.90 (d, *J* = 8.8 Hz, 2H), 4.04 (t, *J* = 6.2 Hz, 2H), 2.65 (t, *J* = 7.2 Hz, 2H), 2.49–2.35 (m, 1H), 2.30 (s, 3H), 2.05–1.89 (m, 2H), 1.90–1.69 (m, 3H), 1.69–1.56 (m, 1H), 1.33–1.02 (m, 6H).

#### Synthesis of (E)-*N*-Methyl-N-(4-(4-styrylphenoxy)­butyl)­cyclohexanamine (**36**)

Obtained from **34** (150 mg, 0.40 mmol), *N*-methylcyclohexanamine (1.0 mL, 7.93 mmol) in toluene (2 mL) following the general METHOD E. Compound **36** was obtained as beige solid in 81% yield. mp = 79–81 °C. *R*_f_ = 0.15 (CH_2_Cl_2_/MeOH 95:5). ^1^H NMR (300 MHz, CDCl_3_) δ 7.53–7.47 (m, 2H), 7.44 (d, *J* = 8.7 Hz, 2H), 7.39–7.30 (m, 2H), 7.26–7.18 (m, 1H), 7.07 (d, *J* = 16.3 Hz, 1H), 6.97 (d, *J* = 16.3 Hz, 1H), 6.89 (d, *J* = 8.7 Hz, 2H), 4.00 (t, *J* = 6.4 Hz, 2H), 2.50 (t, *J* = 7.3 Hz, 2H), 2.44–2.31 (m, 1H), 2.27 (s, 3H), 1.88–1.73 (m, 6H), 1.70–1.56 (m, 2H), 1.33–1.03 (m, 6H).

#### Synthesis of *N*-Methyl-*N*-(2-(4-phenethylphenoxy)­ethyl)­cyclohexanamine (**38**)

**37** (58 mg, 0.173 mmol) was dissolved in MeOH (6 mL). Pd/C (15 mg) was added and the reaction mixture was stirred vigorously under H_2_ atmosphere at rt. After 6 h the reaction mixture was filtered on a short layer of Celite. The Celite was washed with MeOH and the solvent was evaporated affording **38** as a pale brownish oil in 99% yield. ^1^H NMR (300 MHz, CDCl_3_) δ 7.32–7.23 (m, 2H), 7.22–7.12 (m, 3H), 7.08 (d, J = 8.6 Hz, 2H), 6.82 (d, J = 8.6 Hz, 2H), 4.14 (t, *J* = 6.0 Hz, 2H), 2.98 (t, *J* = 6.0 Hz, 2H), 2.87 (s, 4H), 2.69–2.55 (m, 1H), 2.46 (s, 3H), 2.01–1.91 (m, 2H), 1.87–1.78 (m, 2H), 1.72–1.59 (m, 1H), 1.38–1.01 (m, 5H).

#### Synthesis of 1-(Benzyloxy)-4-(2-bromoethoxy)­benzene (**40**)

Obtained from 4-(benzyloxy)­phenol (491 mg, 2.45 mmol), K_2_CO_3_ (847 mg, 6.13 mmol), 1,2-dibromoethane (888 μL, 10.30 mmol) in acetone (5 mL) at reflux, following the general METHOD B. Compound **40** was obtained as white solid in 43% yield. mp = 79–80 °C (lit.^42^ 75–77 °C). *R*_f_ = 0.50 (petroleum ether/EtOAc 95:5). ^1^H NMR (300 MHz, CDCl_3_) δ 7.47–7.28 (m, 5H), 6.95–6.82 (m, 4H), 5.02 (s, 2H), 4.25 (t, *J* = 6.3 Hz, 2H), 3.61 (t, *J* = 6.3 Hz, 2H).

#### Synthesis of 1-(Benzyloxy)-4-(2-iodoethoxy)­benzene (**41**)

Obtained from **40** (323 mg, 1.05 mmol) in a saturated solution of NaI in acetone (7 mL), following the general METHOD D. Compound **41** was obtained as white solid in 91% yield. mp = 75–76 °C (lit.^43^ 80–81 °C). *R*_f_ = 0.48 (petroleum ether/EtOAc 98:2). ^1^H NMR (300 MHz, CDCl_3_) δ 7.46–7.30 (m, 5H), 6.96–6.81 (m, 4H), 5.02 (s, 2H), 4.20 (t, *J* = 6.9 Hz, 2H), 3.39 (t, *J* = 6.9 Hz, 2H).

#### Synthesis of *N*-(2-(4-(Benzyloxy)­phenoxy)­ethyl)-*N*-methylcyclohexanamine (**42**)

Obtained from **41** (175 mg, 0.49 mmol), *N*-methylcyclohexanamine (1.3 mL, 9.88 mmol) in toluene (2.5 mL) following the general METHOD E. Compound **42** was obtained as light orange amorphous solid in 80% yield. *R*_f_ = 0.28 (CH_2_Cl_2_/MeOH 95:5). ^1^H NMR (300 MHz, CDCl_3_) δ 7.48–7.28 (m, 5H), 6.90 (d, *J* = 9.3 Hz, 2H), 6.83 (d, *J* = 9.3 Hz, 2H), 5.01 (s, 2H), 4.03 (t, *J* = 6.3 Hz, 2H), 2.88 (t, *J* = 6.3 Hz, 2H), 2.56–2.44 (m, 1H), 2.40 (s, 3H), 1.93–1.76 (m, 4H), 1.69–1.60 (m, 1H), 1.35–0.99 (m, 5H).

#### Synthesis of (4-(2-Bromoethoxy)­phenyl)­methanol (**44**)

^44^Obtained from 4-(hydroxymethyl)­phenol (786 mg, 6.33 mmol), K_2_CO_3_ (2.19 g, 15.83 mmol), 1,2-dibromoethane (2.3 mL, 26.59 mmol) in acetone (13 mL) at reflux, following the general METHOD B. Compound **44** was obtained as white solid in 38% yield. mp = 73–75 °C. *R*_f_ = 0.55 (CH_2_Cl_2_/EtOAc 7:3). ^1^H NMR (300 MHz, CDCl_3_) δ 7.30 (d, *J* = 8.7 Hz, 2H), 6.91 (d, *J* = 8.7 Hz, 2H), 4.63 (d, *J* = 4.2 Hz, 2H), 4.30 (t, *J* = 6.3 Hz, 2H), 3.64 (t, *J* = 6.3 Hz, 2H).

#### Synthesis of (4-(2-Iodoethoxy)­phenyl)­methanol (**45**)

Obtained from **44** (564 mg, 2.44 mmol) in a saturated solution of NaI in acetone (17 mL), following the general METHOD D. Compound **45** was obtained as white solid in 91% yield. mp = 70–71 °C. *R*_f_ = 0.56 (cyclohexane/EtOAc 1:1). ^1^H NMR (300 MHz, CDCl_3_) δ 7.30 (d, *J* = 8.6 Hz, 2H), 6.90 (d, *J* = 8.6 Hz, 2H), 4.63 (s, 2H), 4.26 (t, *J* = 6.9 Hz, 2H), 3.42 (t, *J* = 6.9 Hz, 2H).

#### Synthesis of (4-(2-(Cyclohexyl­(methyl)­amino)­ethoxy)­phenyl)­methanol (**46**)

Obtained from **45** (618 mg, 2.22 mmol), *N*-methylcyclohexanamine (5.8 mL, 44.45 mmol) in toluene (10 mL) following the general METHOD E. Compound **46** was obtained as light brown oil in 99% yield. *R*_f_ = 0.18 (CH_2_Cl_2_/MeOH 95:5). ^1^H NMR (300 MHz, CDCl_3_) δ 7.27 (d, *J* = 8.7 Hz, 2H), 6.89 (d, *J* = 8.7 Hz, 2H), 4.61 (s, 2H), 4.04 (t, *J* = 6.3 Hz, 2H), 2.86 (t, *J* = 6.3 Hz, 2H), 2.53–2.41 (m, 1H), 2.37 (s, 3H), 1.92–1.56 (m, 5H), 1.34–1.04 (m, 5H).

#### Synthesis of *N*-Methyl-*N*-(2-(4-(phenoxymethyl)­phenoxy)­ethyl)­cyclohexanamine (**47**)

Obtained from phenol (189 mg, 2.01 mmol), **46** (530 mg, 2.01 mmol), PPh_3_ (633 mg, 2.41 mmol), DIAD (474 μL, 2.41 mmol) in *anhydrous* THF (7 mL), following the general METHOD C. The volatiles were evaporated and purification by flash chromatography on silica gel (gradient of CH_2_Cl_2_/acetone from 0 to 50% acetone) afforded **47** as yellow oil in 55% yield. *R*_f_ = 0.42 (DCM/MeOH 95:5 + 0.1% TEA); ^1^H NMR (300 MHz, CDCl_3_) δ 7.35–7.18 (m, 4H), 6.98–6.78 (m, 5H), 4.95 (s, 2H), 4.03 (t, *J* = 6.3 Hz, 2H), 2.85 (t, *J* = 6.3 Hz, 2H), 2.50–2.39 (m, 1H), 2.36 (s, 3H), 1.92–1.69 (m, 4H), 1.38–0.95 (m, 6H).

### Biological Assays

#### *Xenopus laevis* Oocyte Preparation and Microinjection

The protocols were approved by the Animal Ethics Committees of the Victor Chang Cardiac Research Institute, Sydney, and the University of Wollongong (project number AE 20/17). Human α9 and α10 nAChR clones were obtained from OriGene (Rockville, MD, USA) and subcloned into the pT7TS vector. The human α7 nAChR clone, in the plasmid pMXT, was kindly provided by J. Lindstrom (University of Pennsylvania, USA). Constructs of human nAChR subunits were linearized and used for *in vitro* cRNA transcription with the SP6/T7 mMessage mMachine transcription kit (AMBION, Forster City, CA, USA).

A maximum of 4 female *X. laevis* were housed in a 15L aquarium maintained at 20–26 °C with a controlled light–dark cycle. Stage V–VI oocytes (Dumont’s classification; 1200–1300 μm diameter) were harvested from five-year-old frogs anesthetized with 1.7 mg/mL ethyl 3-aminobenzoate methanesulfonate (adjusted to pH 7.4, adjusted with NaHCO_3_). The oocytes were then defolliculated by incubation with 1.5 mg/mL collagenase Type II (GIBCO, Grand Island, NY, USA) at room temperature for 1 h in OR-2 solution containing (in mM): 82.5 NaCl, 2 KCl, 1 MgCl_2_ and 5 HEPES (pH 7.4).

Oocytes were microinjected with 10 ng cRNA for α7 nAChRs or 35 ng cRNA for α9 and α9α10 nAChRs (cRNA ratio was 1:1) using glass pipettes (3-000-203 GX, Drummond Scientific Co., Broomall, PA, USA). The cRNA concentration was verified spectrophotometrically and by gel electrophoresis. The injected oocytes were incubated at 18 °C in sterile ND96 solution, composed of (in mM): 96 NaCl, 2 KCl, 1 CaCl_2_, 1 MgCl_2_, and 5 HEPES (pH 7.4), supplemented with 5% fetal bovine serum (Bovogen, East Keilor, VIC, Australia), 0.1 mg/L gentamicin (GIBCO), and 100 U/mL penicillin–streptomycin (GIBCO).

### Oocyte Two-Electrode Voltage Clamp Recording and Data Analysis

Electrophysiological recordings were conducted 2–5 days after cRNA injection using a HiClamp automated two-electrode voltage-clamp screening system (MultiChannel Systems GmbH, Reutlingen, Germany) with a holding potential of –80 mV. To prevent the activation of endogenous Ca^2+^-activated chloride channels due to the Ca^2+^ permeability of α9-containing nAChRs, oocytes were preincubated with 100 μM BAPTA-AM for approximately 3 h. Oocytes expressing α7 nAChRs oocytes were superfused with ND96 solution, whereas oocytes expressing α9-containing nAChRs were superfused with ND115 solution containing (in mM): 115 NaCl, 2.5 KCl, 1.8 CaCl_2_, and 10 HEPES (pH 7.4).

Concentration–response experiments assessing compound-evoked currents were performed by initially superfusing α9-containing nAChRs oocytes with ND115 solution. Test compounds were then applied at concentrations ranging from 300 pM to 30 μM, followed by a 3 min washout period.

For concentration–response experiments assessing modulation of ACh-evoked responses, oocytes were initially superfused with ND96 or ND115 solution, depending on the receptor subtype. ACh was then applied at the half-maximal effective concentration (EC_50_) for each receptor subtype: 6 μM for α9α10, 50 μM for α9, and 100 μM for α7 nAChRs. After ACh application, a 3 min washout was performed. Oocytes were then incubated with the test compound for 5 min, followed by coapplication of the compound and ACh in a continuously flowing bath solution. All compounds were prepared in ND96 or ND115 with 0.1% bovine serum albumin (BSA).

### IL-1β Release Experiments

Experiments on human monocytic THP-1 cells (German Collection of Microorganisms and Cell Cultures, Braunschweig, Germany, Cat# ACC16) were performed as described before. In brief, cells were cultured in RPMI 1640 medium (Capricorn Ebsdorfergrund, Germany, Cat# RPMI-A) supplemented with 10% vol/vol FBS from Capricorn (Cat# FBS-16A) under 5% CO_2_ atm at 37 °C. On the day of the experiment, cells were resuspended in FBS-free RPMI medium and seeded in a density of 0.5 × 10^6^ cells/0.5 mL per well in 48-well plates. Cells were primed with 1 μg/mL LPS (*E. coli* O26:B6, Merck, Darmstadt, Germany, Cat# L2654) for 5 h, followed by 40 min of BzATP (2′(3′)-O-(4-benzoyl–benzoyl)­ATP triethylammonium salt; Jena Bioscience, Jena, Germany, Cat# NU-1620-5) treatment with or without test compounds. The supernatants were collected and stored for later IL-1β measurements using the Human IL-1β/IL-1F2 DuoSet ELISA from R&D Systems (Minneapolis MN, USA, Cat# DY201). Cell viability was assessed by measuring lactate dehydrogenase (LDH) activity in the supernatants using the CytoTox 96 Non-Radioactive Cytotoxicity Assay (Promega, Madison WI, USA; Cat# G1780). LDH activities were expressed as a percentage of total LDH activity from lysed control cells. Data on the BzATP-induced IL-1β release and LDH activity were analyzed using SPSS (Version 29.0.2.0, IBM, Armonk, NY, United States) and visualized using Inkscape version 0.48.5 r10040 (Free and Open Source Software licensed under the GPL). The data were analyzed first by the Friedman test. When the *P* value was below 0.05, the two-tailed Wilcoxon signed-rank test was performed for pairwise comparison.

### Modeling

The extracellular domain (ECD) of the human α9 nAChR was constructed using Modeller v9,^45^ with the apo form of nAChR (PDB ID: 7EKI)^46^ serving as the structural template. Molecular docking calculations were performed using Autodock Vina^47,48^ to predict the binding poses of compounds **21** and **22** at the hα9 nAChR. PDB structures were converted to PDBQT format, and other preprocessing tasks were completed using the PyRx^49^ graphical user interface (GUI). The docking grid box was centered on the interior of the loop C agonist binding pocket, with box dimensions of 22.5 × 15 × 15 Å^3^. The lengthiest axis of the box was aligned parallel to the long axis of the ECD subunit. All rotatable torsion angles of compounds **21** and **22** were set as flexible, while the receptor was treated as rigid. An exhaustiveness value of 128 was used for all docking calculations.

MD simulations were conducted using GROMACS^50^ versions 2023 and 2024 with the CHARMM27 ^51^ force field. To simulate low ligand concentrations, ligands were docked only to the five intersubunit agonist binding sites of the pentameric ECD. To simulate higher concentrations, “flooding” simulations were performed in which, additional copies of each compound were randomly placed in the solvent surrounding the ECD: 5 additional ligands were added for moderate concentration simulations, and 15 for high concentration simulations.

Force field parameters for compounds **21** and **22** were generated using the SwissParam server.^52^ Ligand–receptor complexes were solvated in a dodecahedral simulation box filled with TIP3P water molecules, maintaining a minimum distance of 2.0 nm between any protein atoms to the closest box edge. Na^+^ and Cl^–^ were added to achieve an approximate salt concentration of 150 mM and to neutralize the overall system charge. Energy minimization was performed using the steepest-descent gradient. Equilibrium simulations were performed under isothermal-isochloric (NVT) and isothermal–isobaric (NPT) conditions for 1 ns, with positional restraints applied to all non-hydrogen atoms. The system temperature and pressure were maintained at 310 K and 1.0 bar using a modified Berendsen^53^ thermostat and a Parrinello–Rahman^54^ barostat, respectively. The LINCS^55^ algorithm was used to constrain bond lengths, and long-range electrostatics were calculated using the particle-mesh Ewald scheme (PME)^56^ method with a grid spacing of 0.16 nm. Short-range nonbonded interactions were calculated using cutoff distances of 1.2 nm for both Coulombic and van der Waals interactions. Following the position-restrained equilibration, production simulations of at least 300 ns were performed for each concentration condition (low, moderate, and high), with simulations run in triplicate using a 2 fs time step. Initial atomic velocities were generated randomly based on a Maxwell–Boltzmann distribution. Trajectory analysis and visualization were carried out using Visual Molecular Dynamics (VMD)^57^ 1.9.3 and ChimeraX.^58–60^ Replicate trajectories for each system were concatenated for analysis.

## Supplementary Material









## Notes

NMR spectra and HPLC chromatograms of key final compounds, 2D ligand–receptor docking (Figure S1), and SwissADME predictions (Table S1) (PDF)
Molecular formula strings (CSV)
Homology model of homomeric hα9 nAChR with docked compound **21** (PDB)
Homology model of homomeric hα9 nAChR with docked compound **22** (PDB)

## Abbreviations Used

AChBP: ACh binding protein
CAS: cholinergic anti-inflammatory system
CNS: central nervous system
ECD: extracellular domain
EC_50_: half-maximal effective concentration
IC_50_: half-maximal inhibitory concentration
IL: interleukin
LPS: lypopolysaccharide
MD: molecular dynamics
nAChR: nicotinic acetylcholine receptor
NCNR: noncanonical nicotinic receptors
TPSA: topological polar surface area
