# Supplementary material for: Dual Agonist/Antagonist Modulation of α9-Containing Nicotinic Acetylcholine Receptors by 2‑Ammoniumethyl Ethers of Stilbenol and Stilbenol Analogues
Source: J Med Chem. 2025 Dec 8;68(24):26099–120. doi: 10.1021/acs.jmedchem.5c02173 (PMC12751000; doi:10.1021/acs.jmedchem.5c02173)
Supplement: Supplementary file 1 [file jm5c02173_si_001.pdf]

Supporting Information

Dual agonist/antagonist modulation of α9-containing nicotinic acetylcholine receptors by 2-ammoniumethyl ethers of stilbenol and stilbenol analogues

Alessandro Giraudo,[1#] Han-Shen Tae,[2#] Andrew Hung,[3] Katrin Richter,[4,5] Bhavana Shivankar,[3,6] Edoardo Armano,[1] Veronika Grau,[4] Marco Pallavicini,[1] David J. Adams,[2] and Cristiano Bolchi[1*]*

[1] Department of Pharmaceutical Sciences, Università degli Studi of Milan, I-20133 Milano, Italy

[2] Molecular Horizons, Faculty of Science, Medicine and Health, University of Wollongong, Wollongong, NSW 2522, Australia

[3] School of Science, STEM College, RMIT University, Melbourne, Victoria 3001, Australia

[4] Department of General and Thoracic Surgery, Laboratory of Experimental Surgery, Justus-Liebig-University, German Center for Lung Research [DZL], Cardio-Pulmonary Institute [CPI], 35385 Giessen, Germany

[5] Department of Natural Sciences, Bonn-Rhein-Sieg University of Applied Sciences, 53359 Rheinbach, Germany

[6] Physical and Materials Chemistry Division, CSIR-National Chemical Laboratory, 41108 Pune, India

Cristiano Bolchi – E-mail: cristiano.bolchi@unimi.it
David J. Adams - E-mail: djadams@uow.edu.au

Index

^{1}H NMR and ^{13}C NMR spectra of final compounds...S3

(E)-N,N-dimethyl-N-(2-(4-styrylphenoxy)ethyl)tetrahydro-2H-pyran-4-aminium iodide (**6**)...S3

(E)-N,N-dimethyl-N-(2-(4-styrylphenoxy)ethyl)cyclopentanaminium iodide (**7**)...S4

(E)-N,N-dimethyl-N-(2-(4-styrylphenoxy)ethyl)cycloheptanaminium iodide (**8**)...S6

(E)-N,N-dimethyl-N-(2-(4-styrylphenoxy)ethyl)benzenaminium iodide (**9**)...S7

(E)-N-benzyl-N,N-dimethyl-2-(4-styrylphenoxy)ethanaminium iodide (**10**)...S8

(E)-N-cyclopentyl-N-methyl-N-(2-(4-styrylphenoxy)ethyl)cyclopentanaminium iodide (**12**)...S9

(E)-N,N-dimethyl-N-(3-(4-styrylphenoxy)propyl)cyclohexanaminium iodide (**14**)...S11

(E)-N,N-dimethyl-N-(4-(4-styrylphenoxy)butyl)cyclohexanaminium iodide (**15**)...S12

N,N-dimethyl-N-(2-(4-phenethylphenoxy)ethyl)cyclohexanaminium iodide (**16**)...S13

N-(2-(4-(benzyloxy)phenoxy)ethyl)-N,N-dimethylcyclohexanaminium iodide (**21**)...S14

N,N-dimethyl-N-(2-(4-(phenoxymethyl)phenoxy)ethyl)cyclohexanaminium iodide (**22**)...S15

HPLC analysis of key final compounds...S16

(E)-N,N-dimethyl-N-(2-(4-styrylphenoxy)ethyl)cyclopentanaminium iodide (**7**)...S16

(E)-N,N-dimethyl-N-(3-(4-styrylphenoxy)propyl)cyclohexanaminium iodide (**14**)...S16

N-(2-(4-(benzyloxy)phenoxy)ethyl)-N,N-dimethylcyclohexanaminium iodide (**21**)...S16

Figure S1...S17

Table S1...S18

References...S19

^{1}H NMR and ^{13}C NMR spectra of final compounds

*(E)-N,N-dimethyl-N-(2-(4-styrylphenoxy)ethyl)tetrahydro-2H-pyran-4-aminium iodide (**6**).*

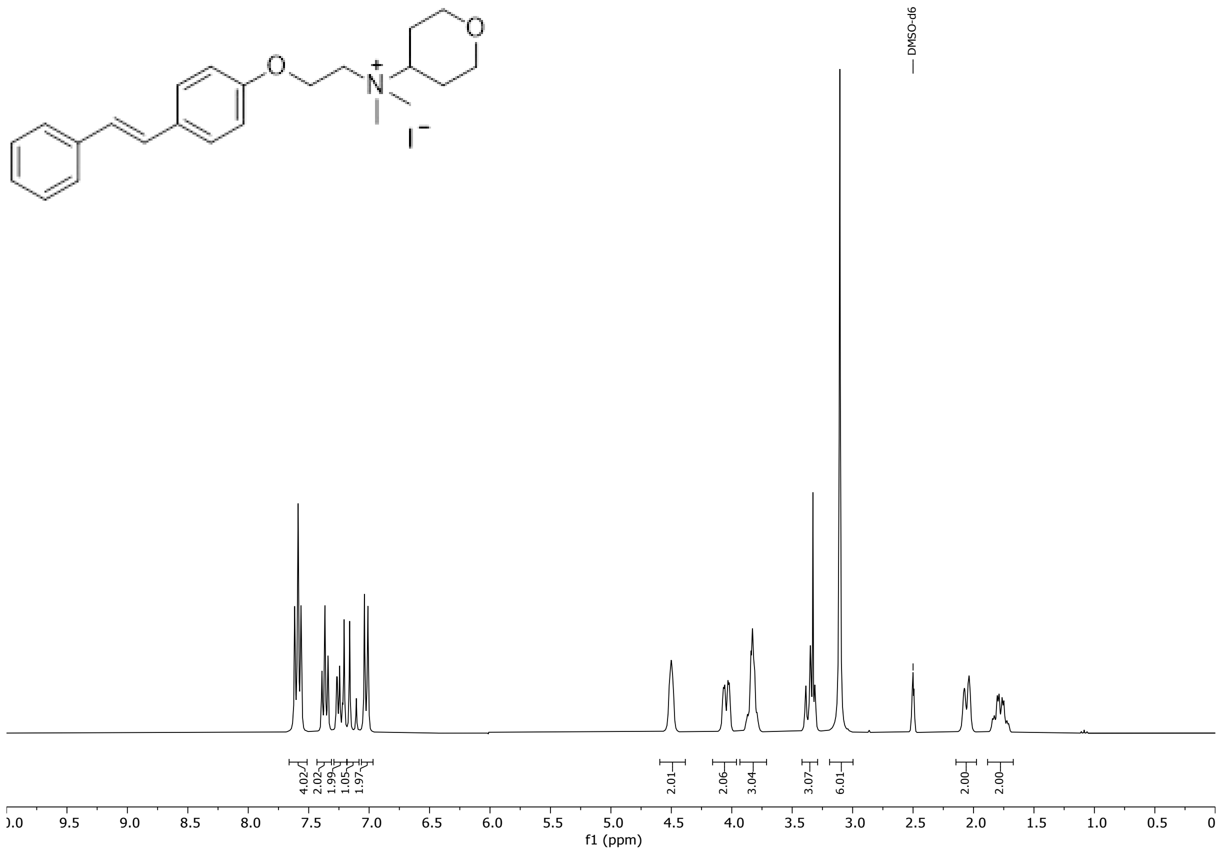

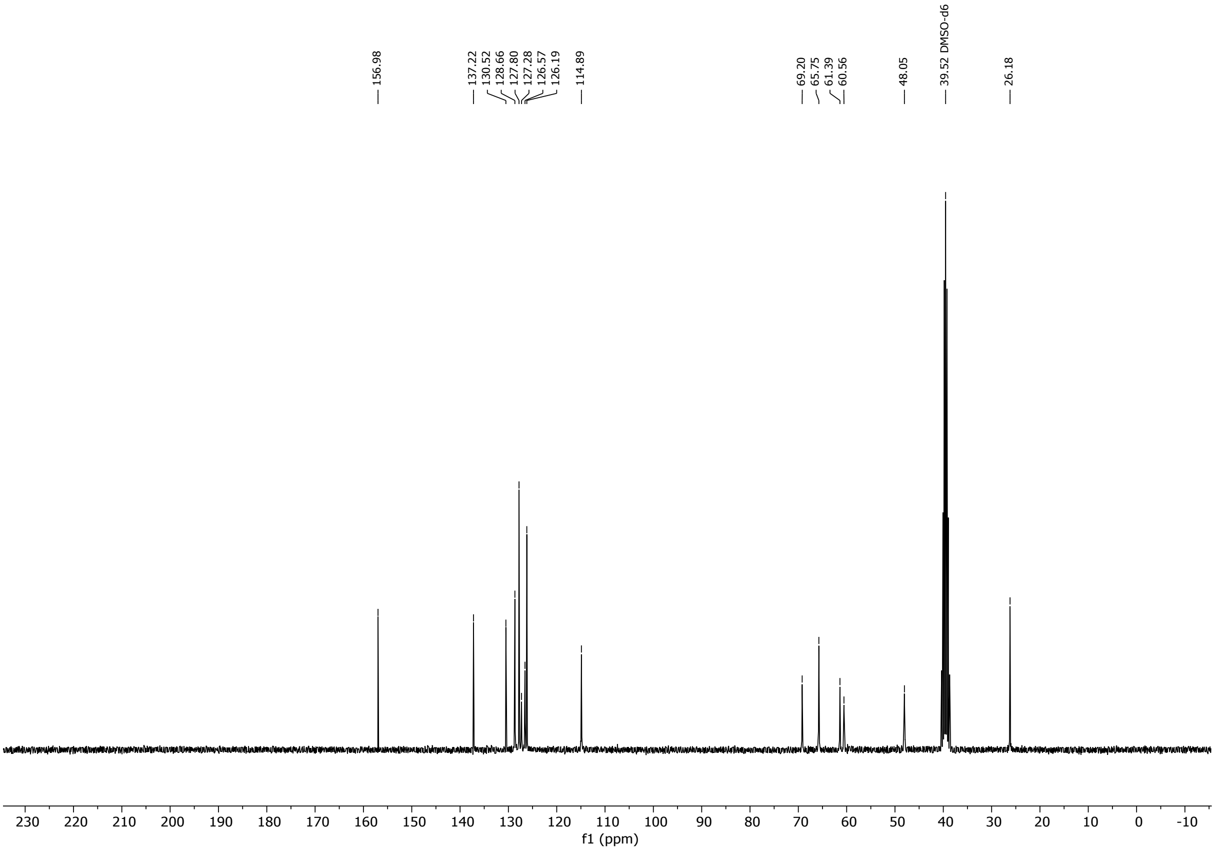

(*E*)-*N*,*N*-dimethyl-*N*-(2-(4-styrylphenoxy)ethyl)cyclopentanaminium iodide (**7**).

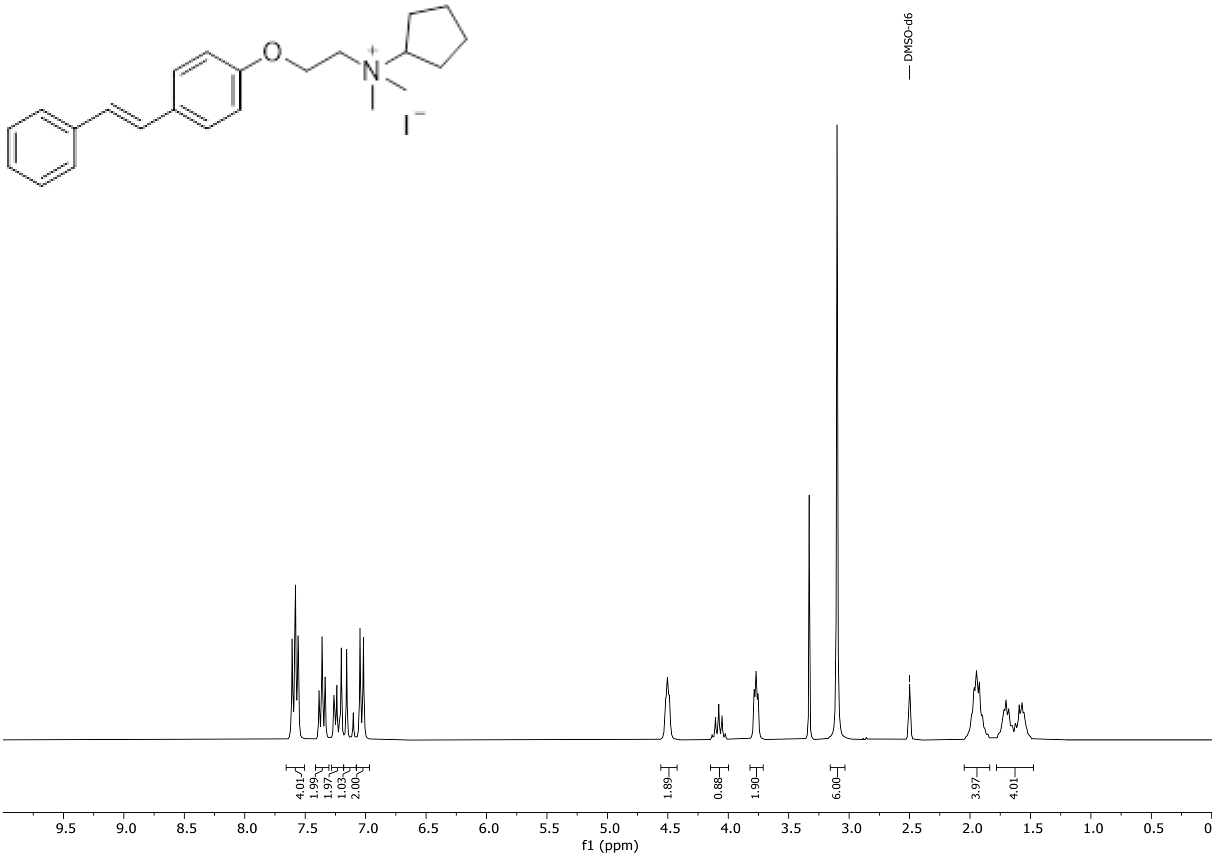

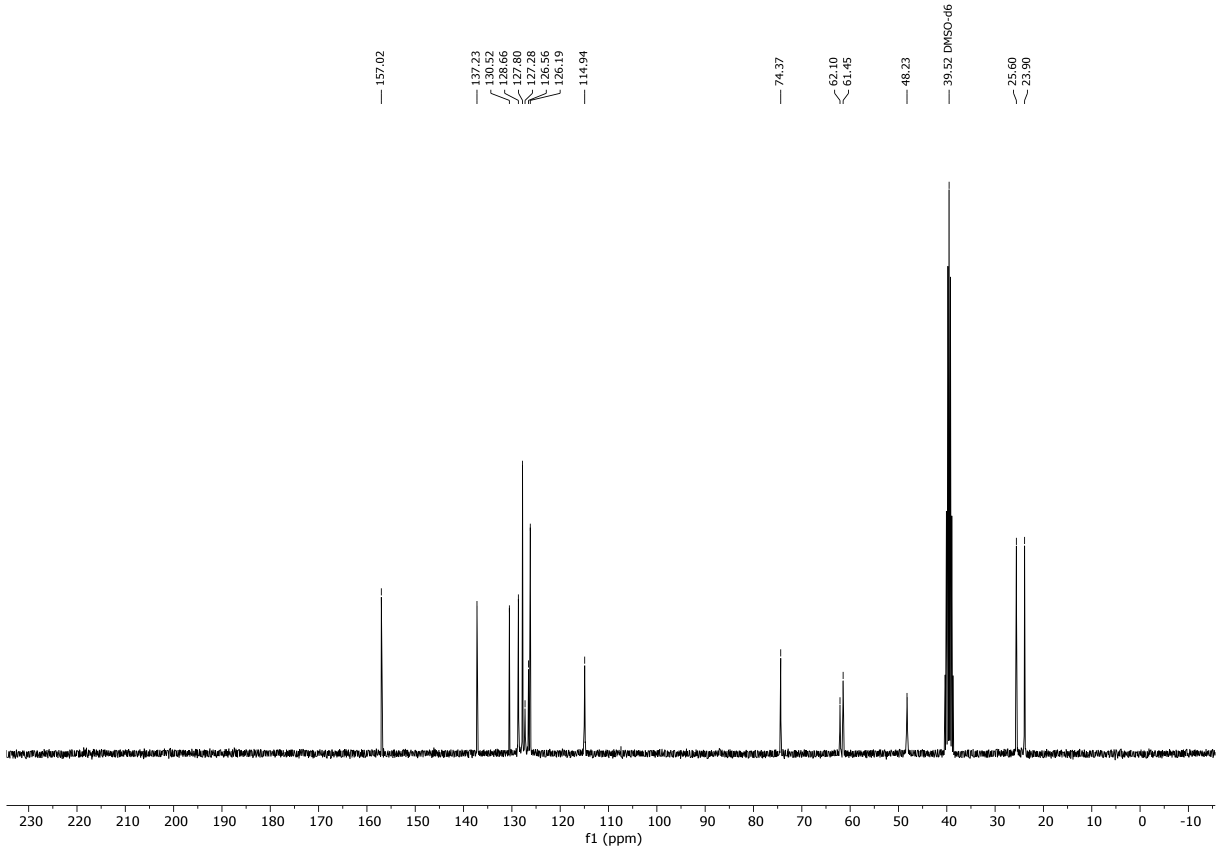
157.02
137.23
130.52
128.66
127.80
127.28
126.56
126.19
114.94
74.37
62.10
61.45
48.23
39.52 DMSO-d6
25.60
23.90
230 220 210 200 190 180 170 160 150 140 130 120 110 100 90 80 70 60 50 40 30 20 10 0 -10
f1 (ppm)

*(E)-N,N-dimethyl-N-(2-(4-styrylphenoxy)ethyl)cycloheptanaminium iodide (**8**).*

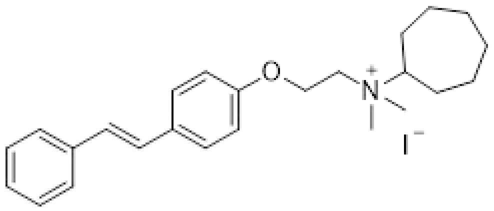

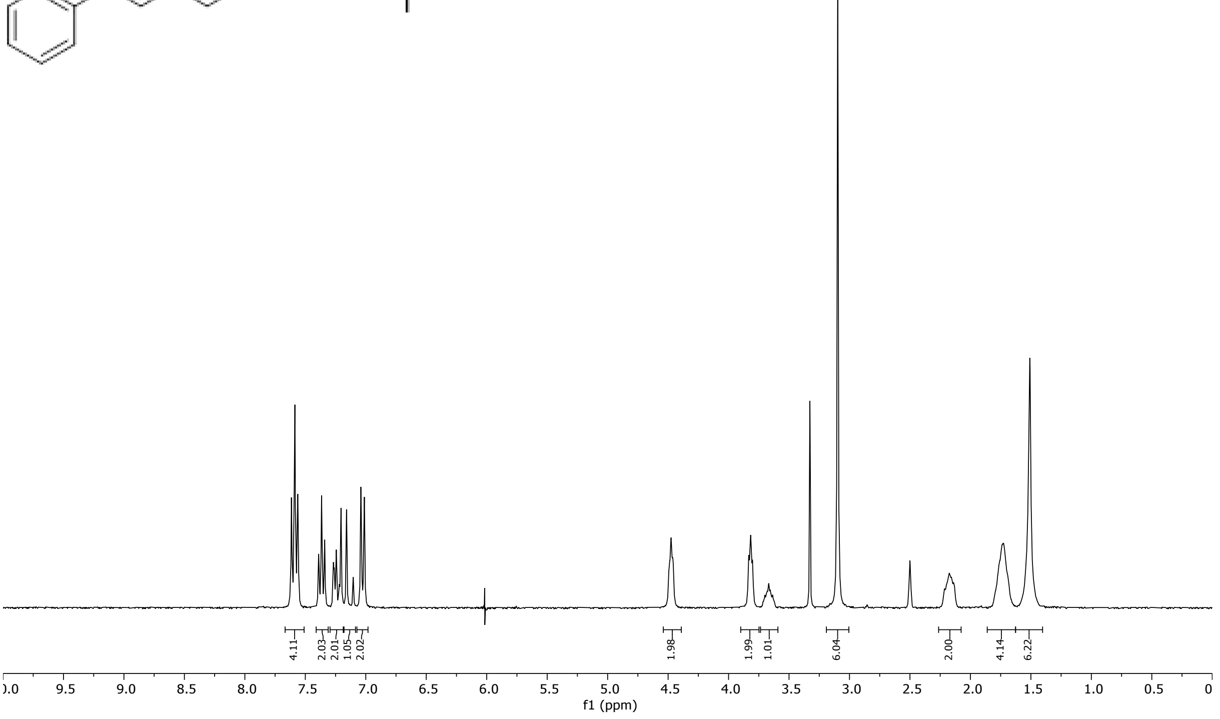

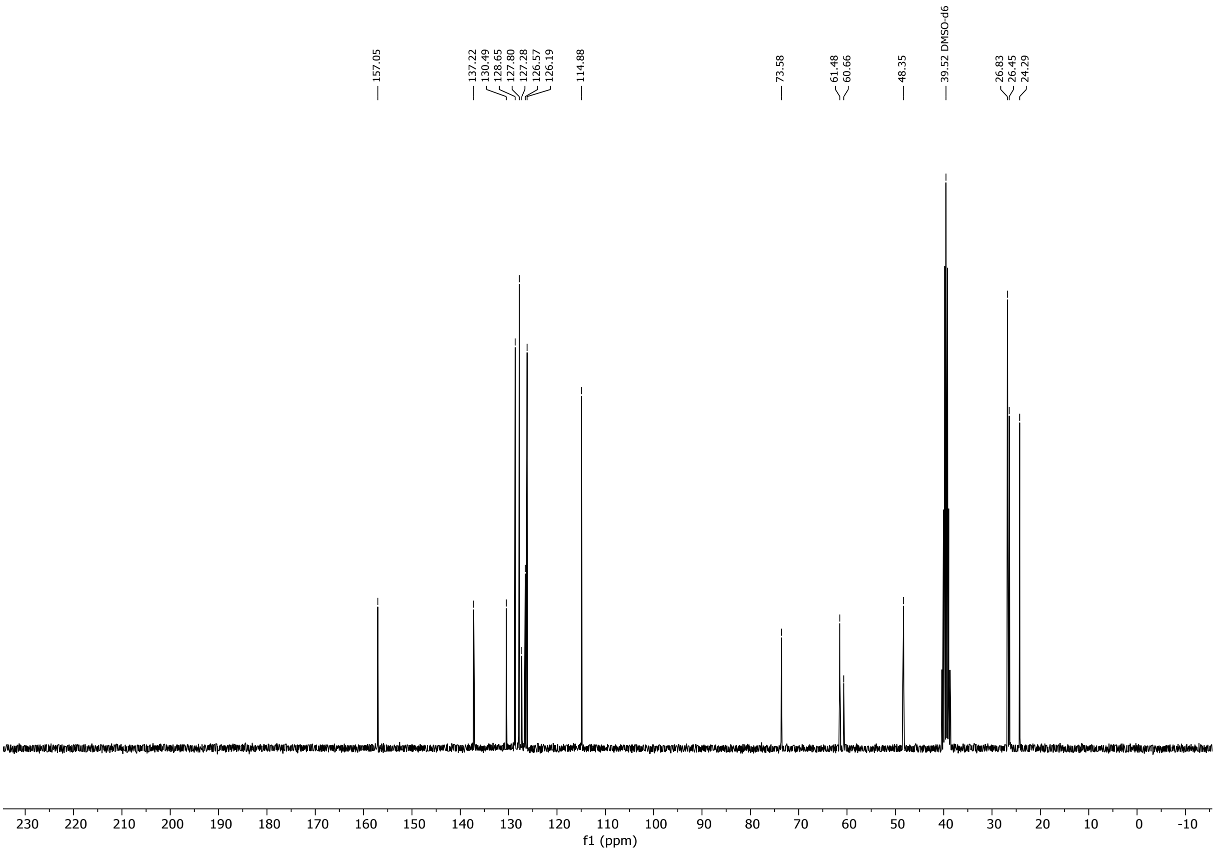

*(E)-N,N-dimethyl-N-(2-(4-styrylphenoxy)ethyl)benzenaminium iodide (**9**).*

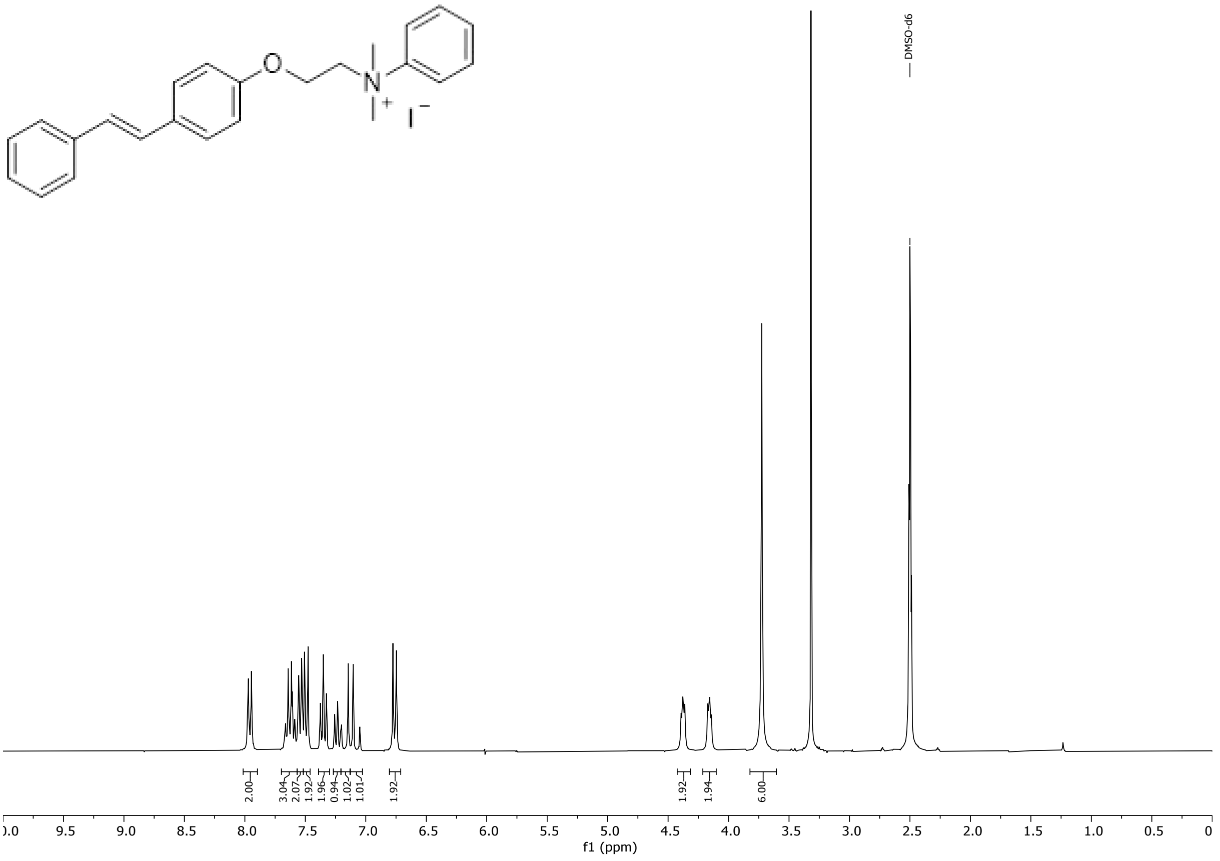

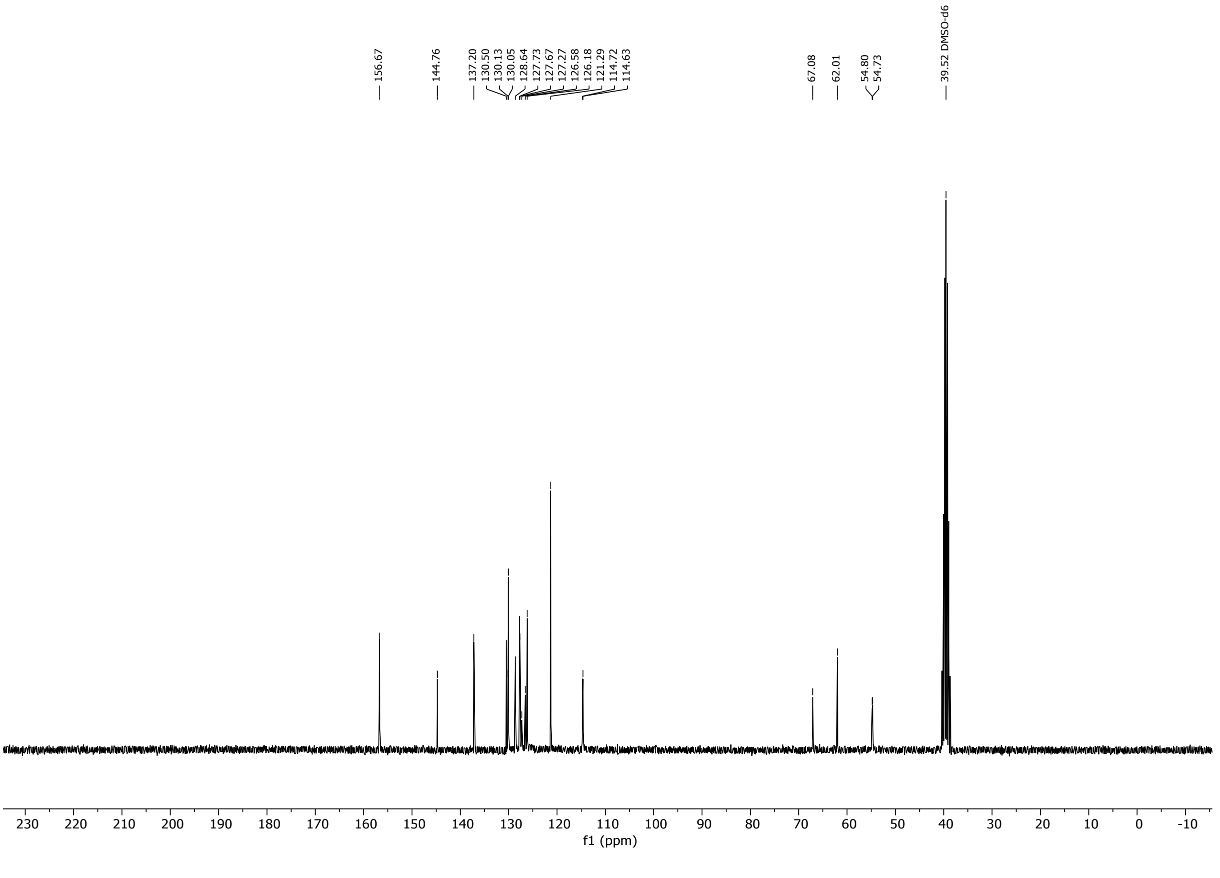

(E)-N-benzyl-N,N-dimethyl-2-(4-styrylphenoxy)ethanaminium iodide (**10**).

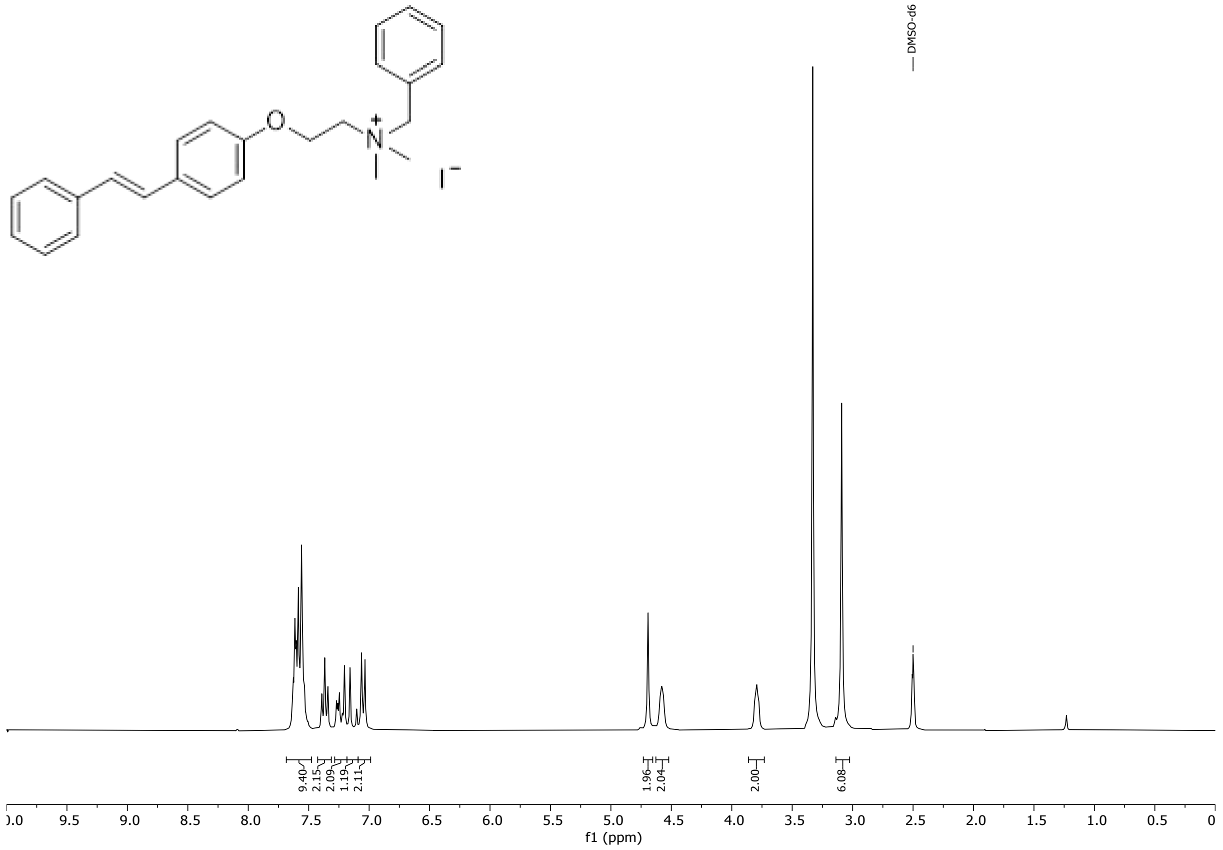

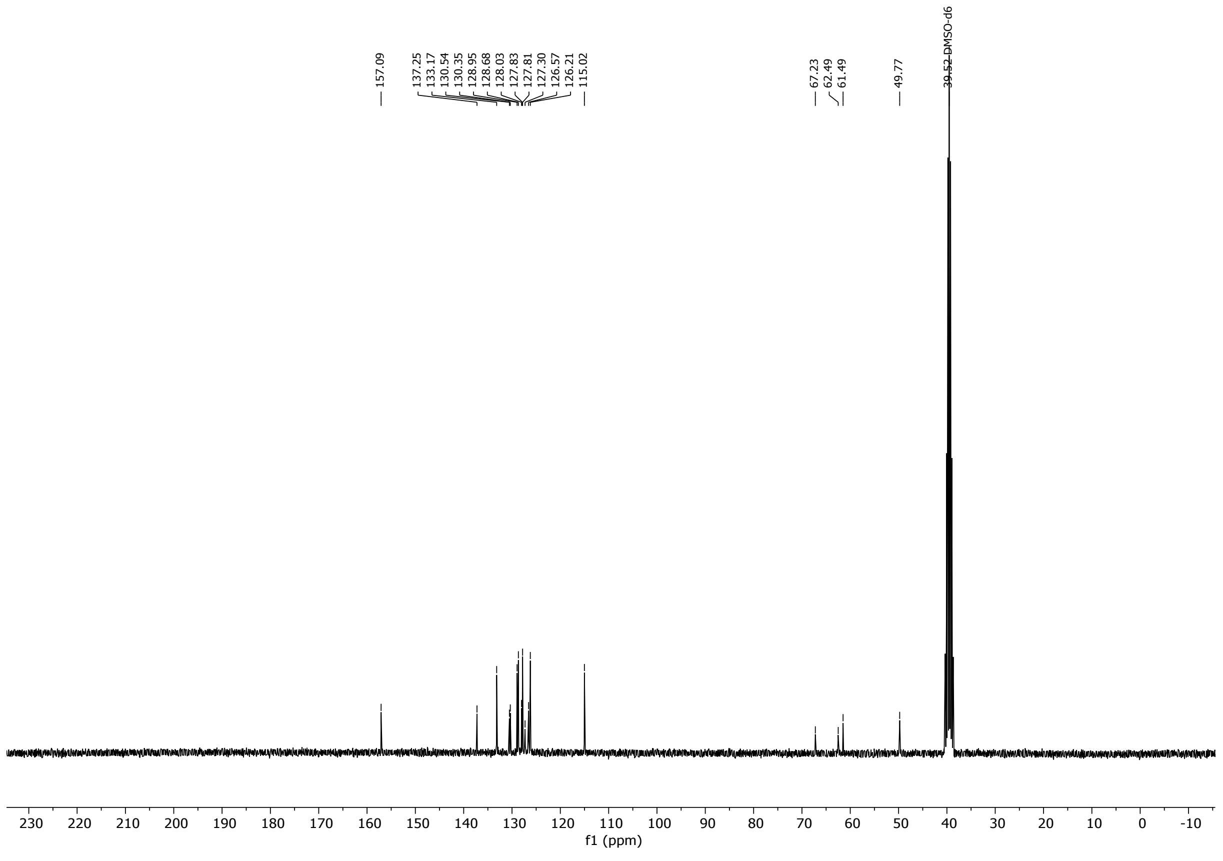

(E)-N-cyclopentyl-N-methyl-N-(2-(4-styrylphenoxy)ethyl)cyclopentanaminium iodide ***(12).***

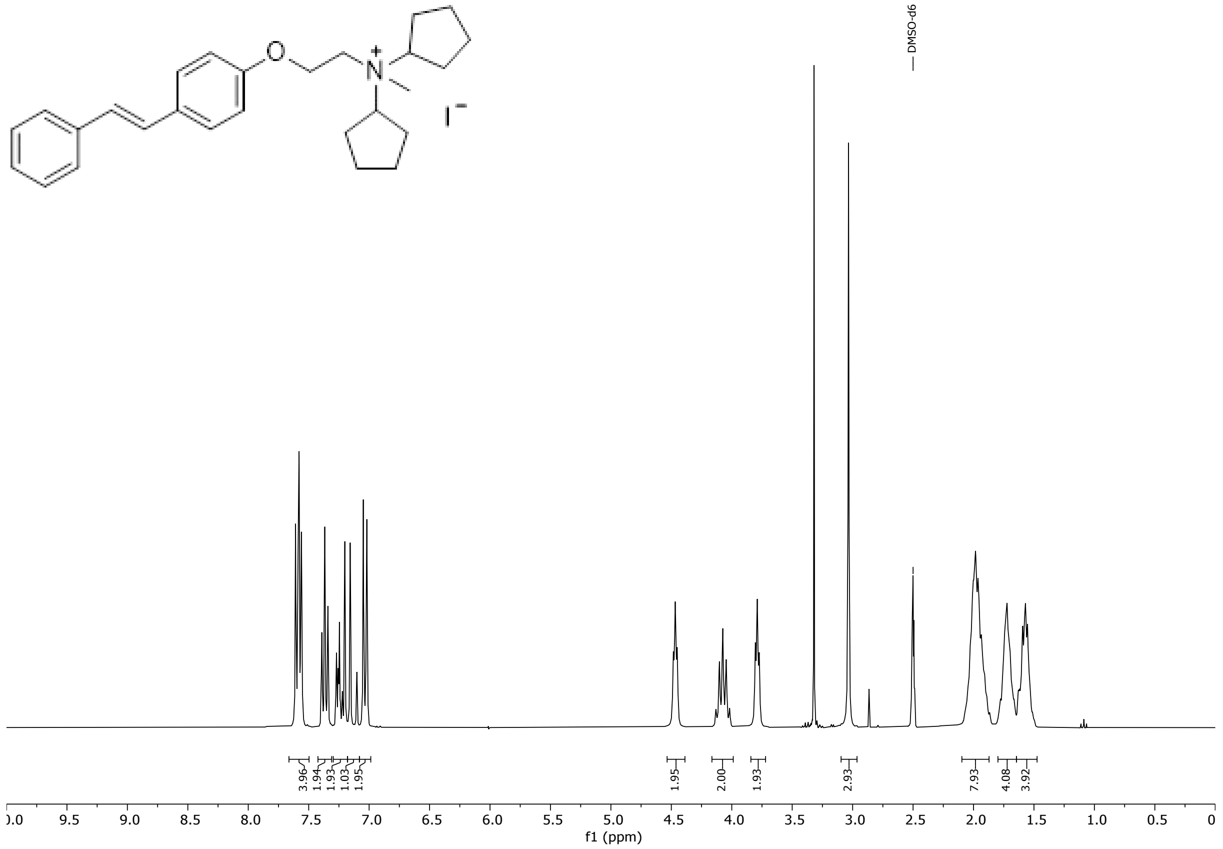

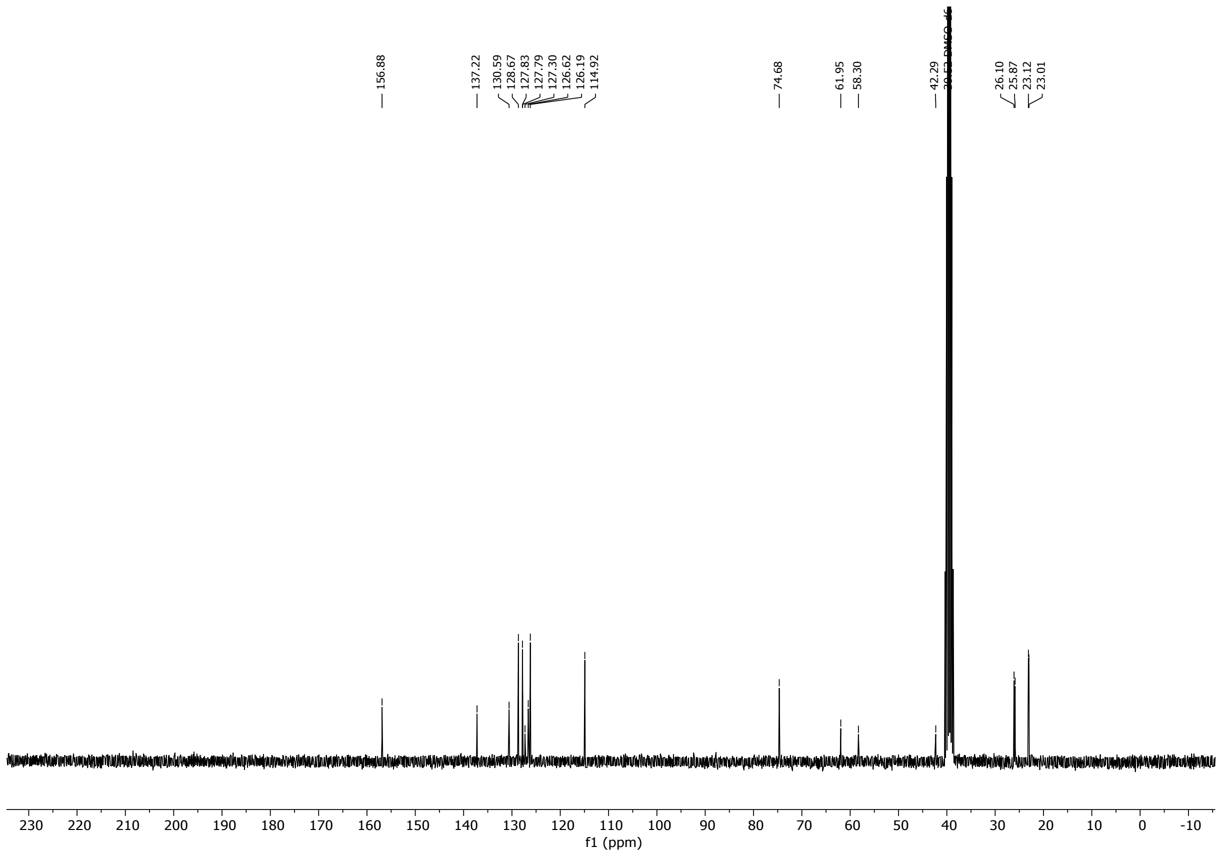

156.88
137.22
130.59
128.67
127.83
127.79
127.30
126.62
126.19
114.92
74.68
61.95
58.30
42.29
26.10
25.87
23.12
23.01
230 220 210 200 190 180 170 160 150 140 130 120 110 100 90 80 70 60 50 40 30 20 10 0 -10
f1 (ppm)

(E)-N,N-dimethyl-N-(3-(4-styrylphenoxy)propyl)cyclohexanaminium iodide (**14**).

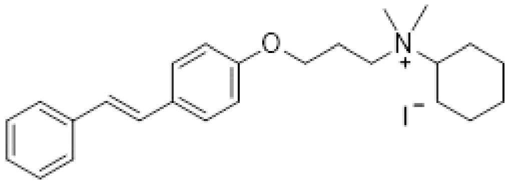

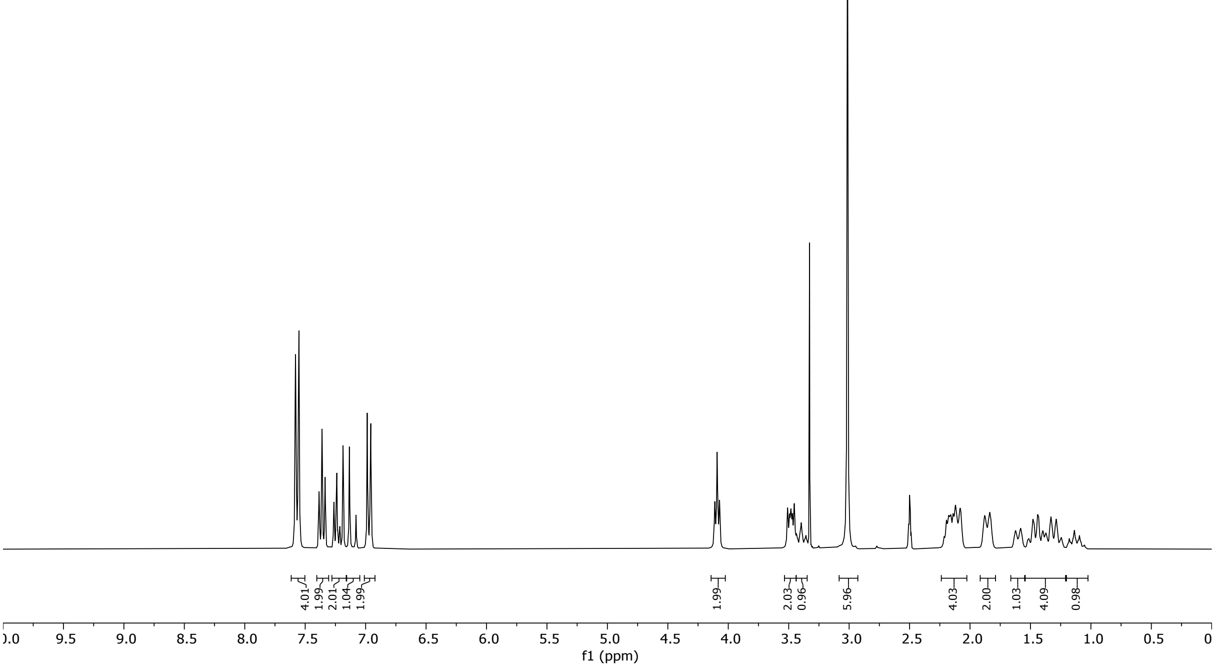

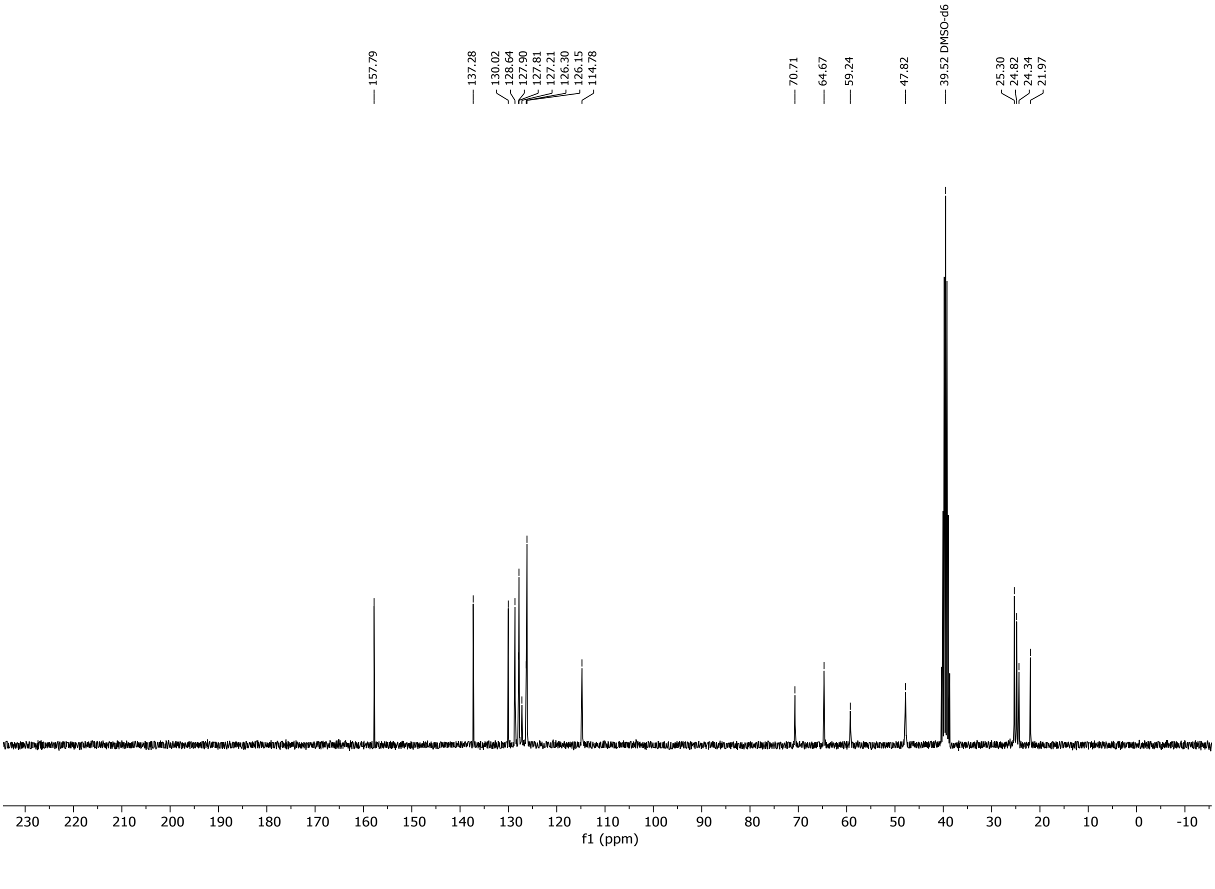

*(E)-N,N-dimethyl-N-(4-(4-styrylphenoxy)butyl)cyclohexanaminium iodide (**15**).*

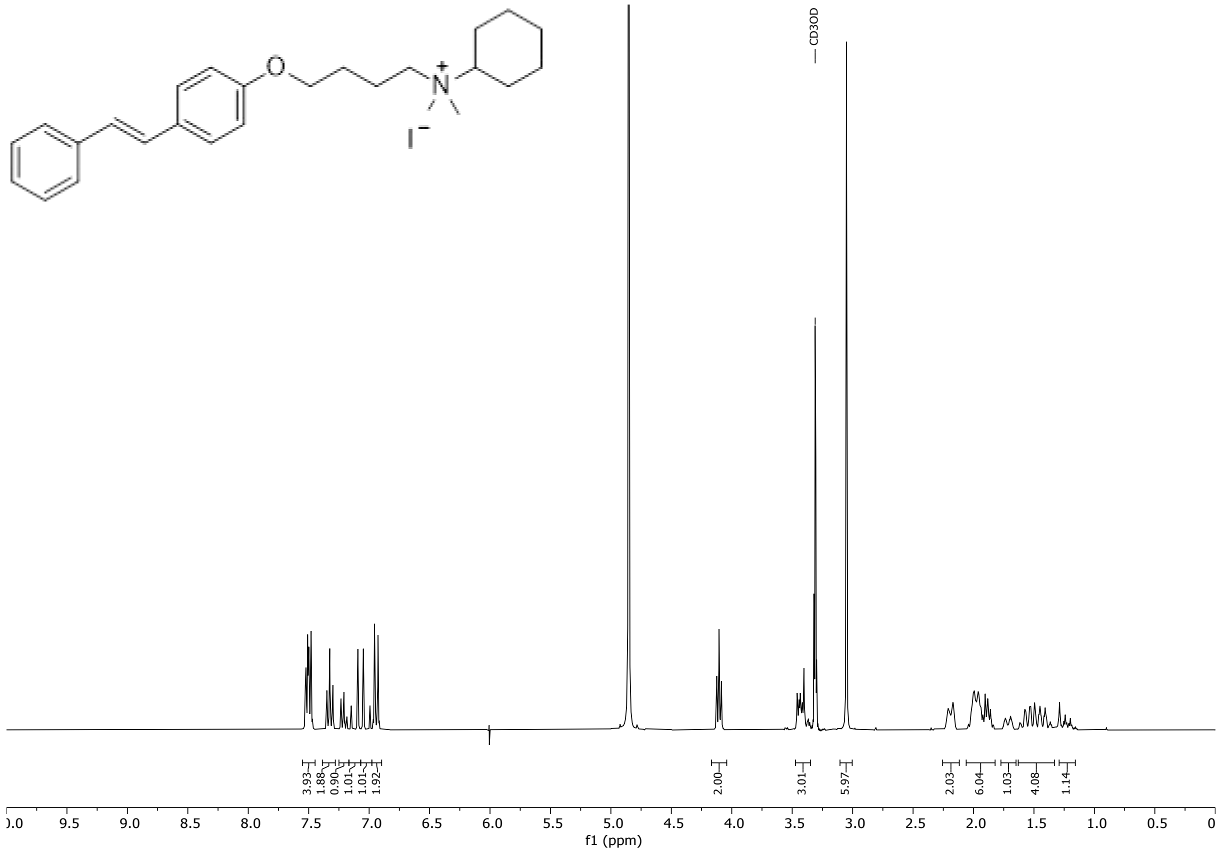

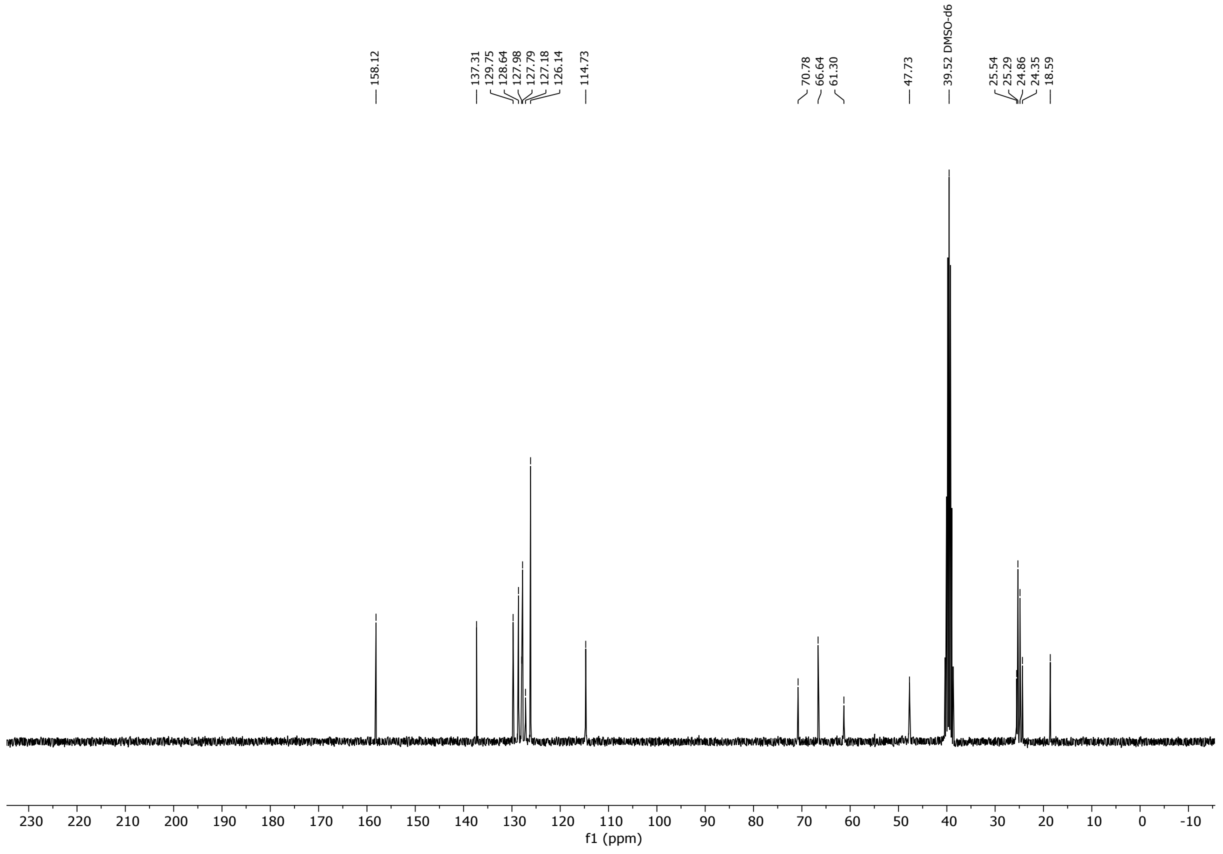

N,N-dimethyl-N-(2-(4-phenethylphenoxy)ethyl)cyclohexanaminium iodide (**16**).

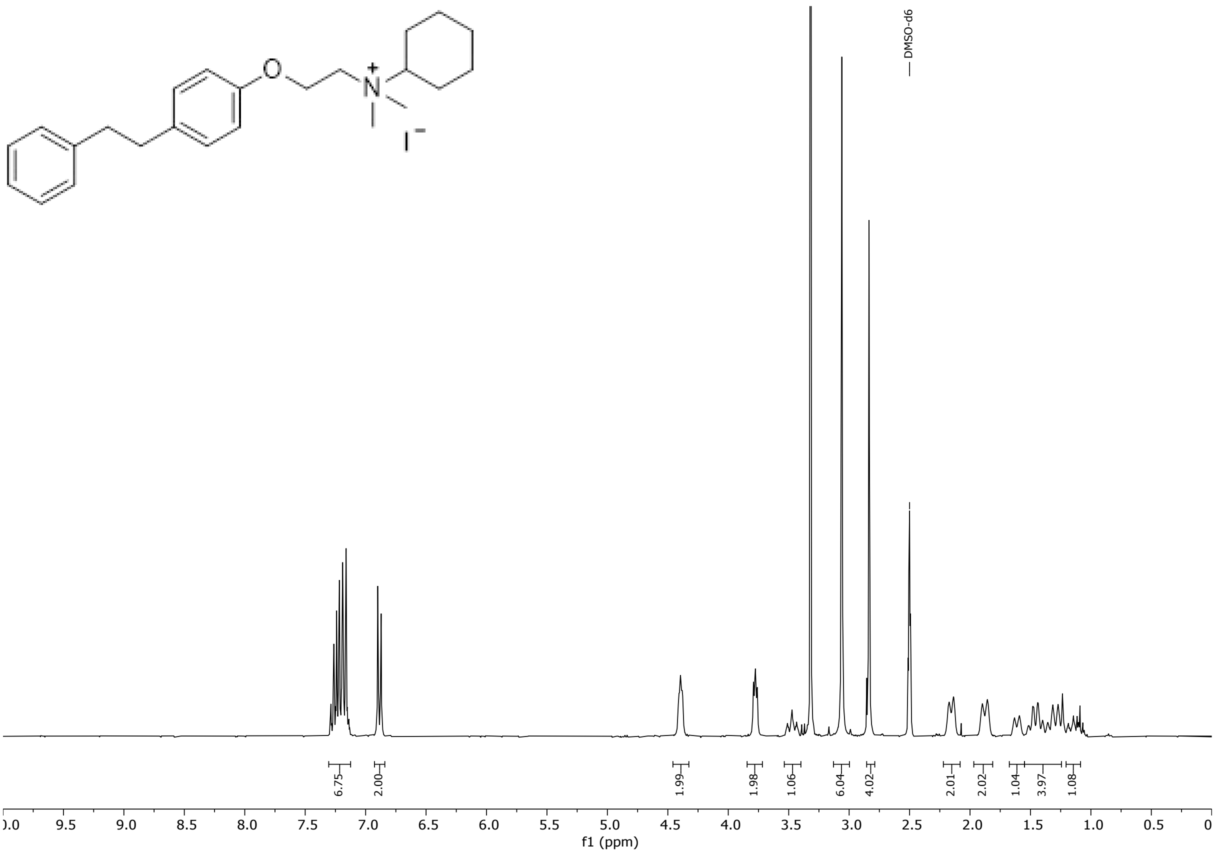

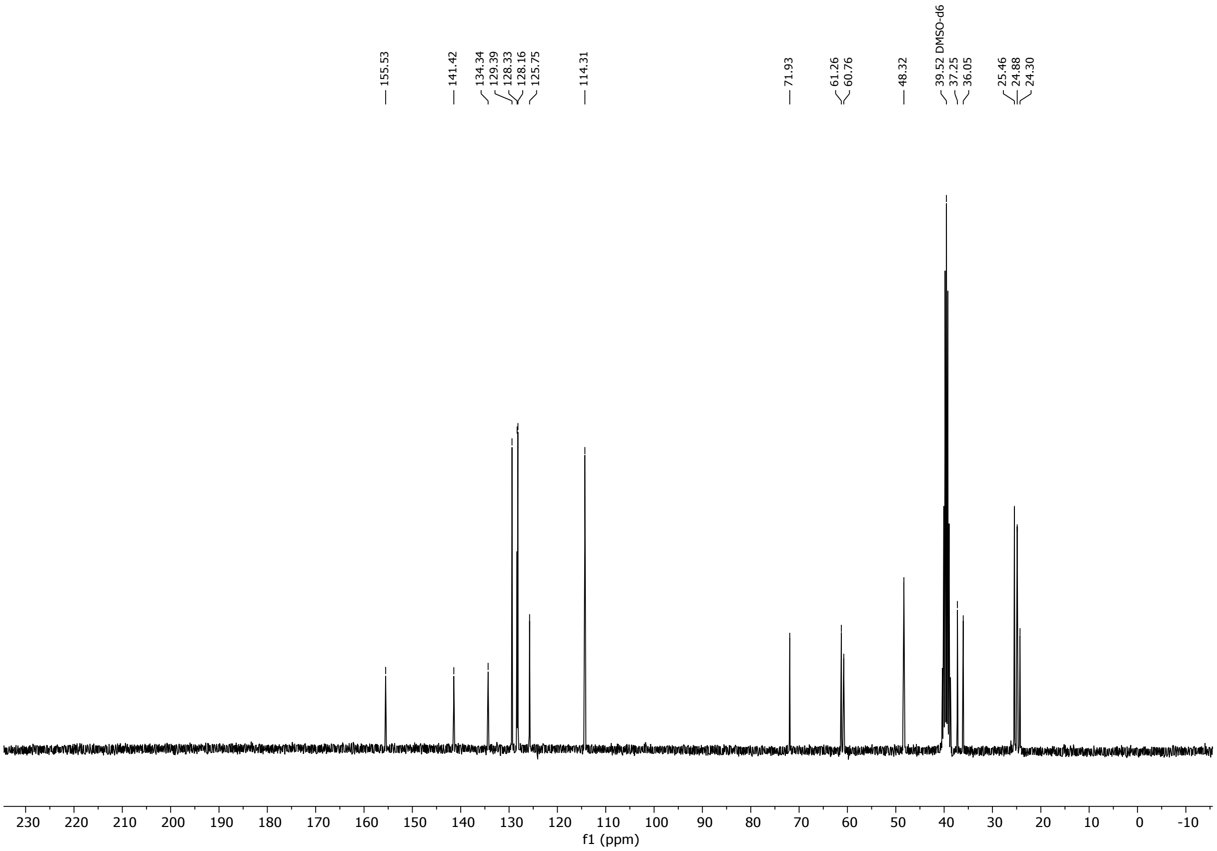

N-(2-(4-(benzyloxy)phenoxy)ethyl)-N,N-dimethylcyclohexanaminium iodide (**21**).

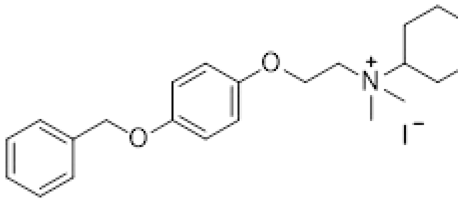

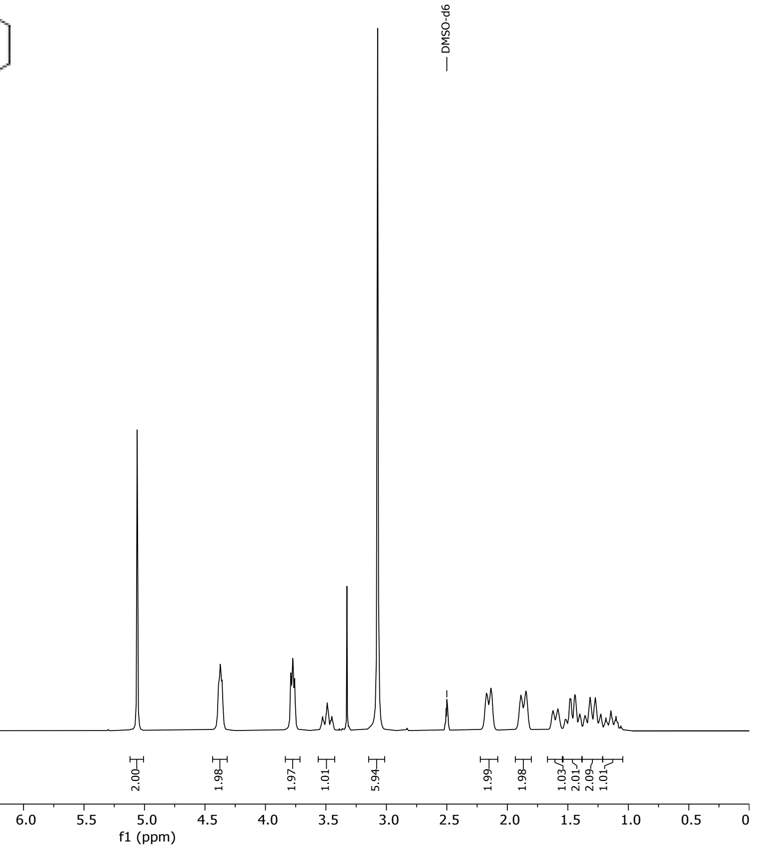

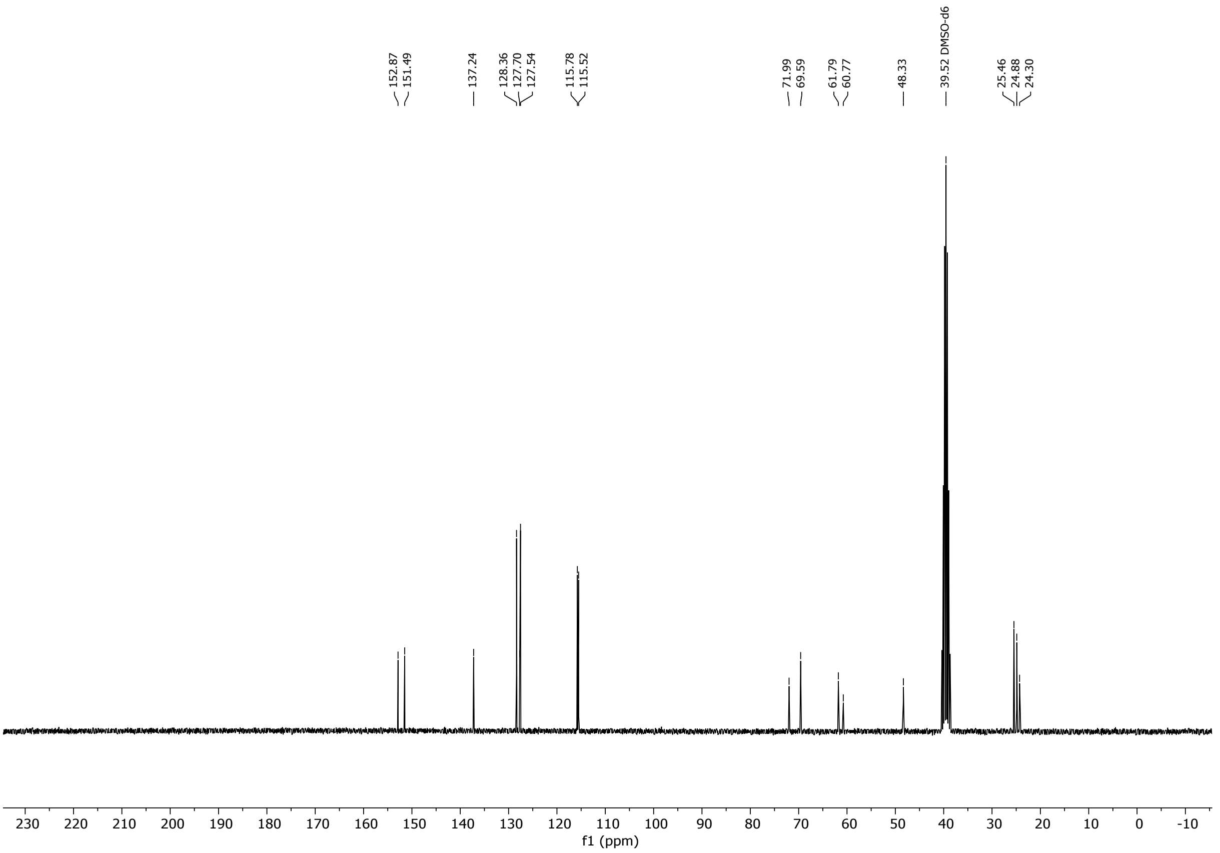

N,N-*dimethyl*-N-*(2-(4-(phenoxymethyl)phenoxy)ethyl)cyclohexanaminium iodide* (**22**).

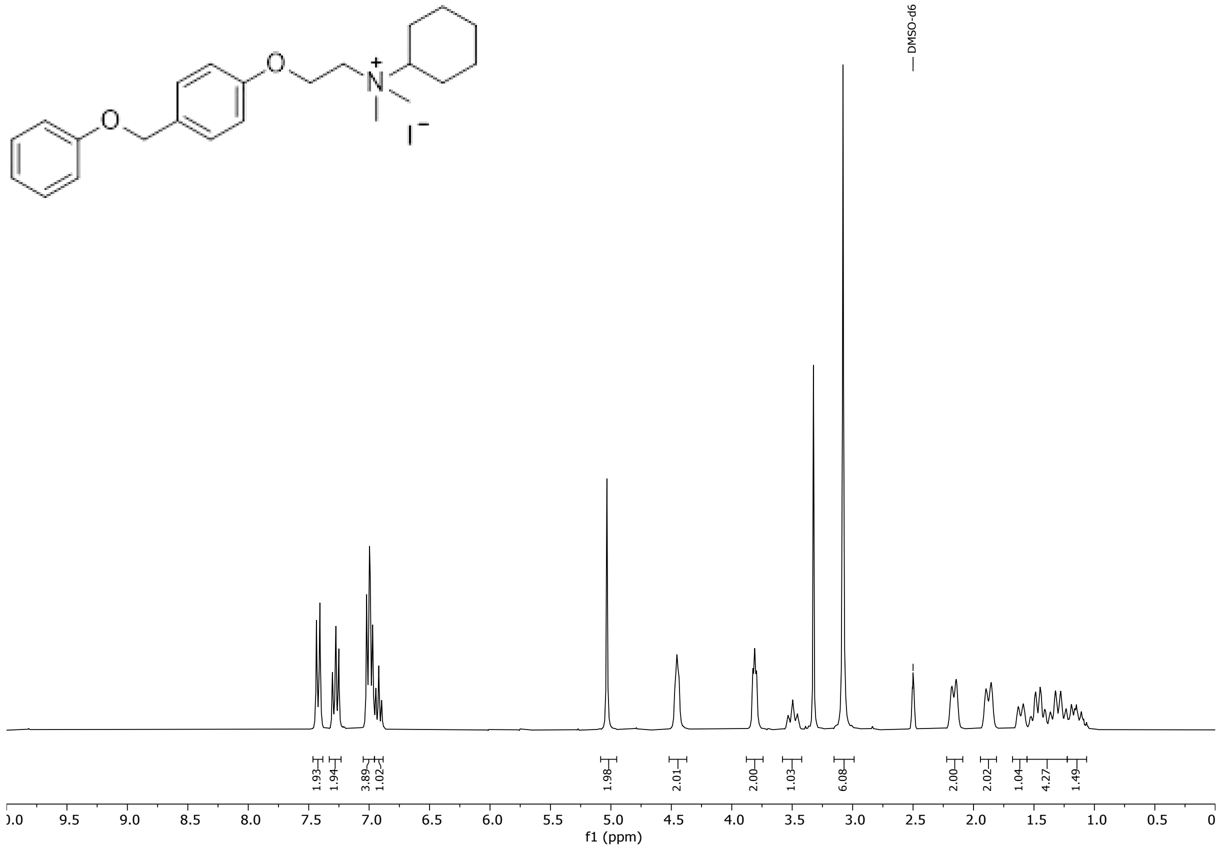

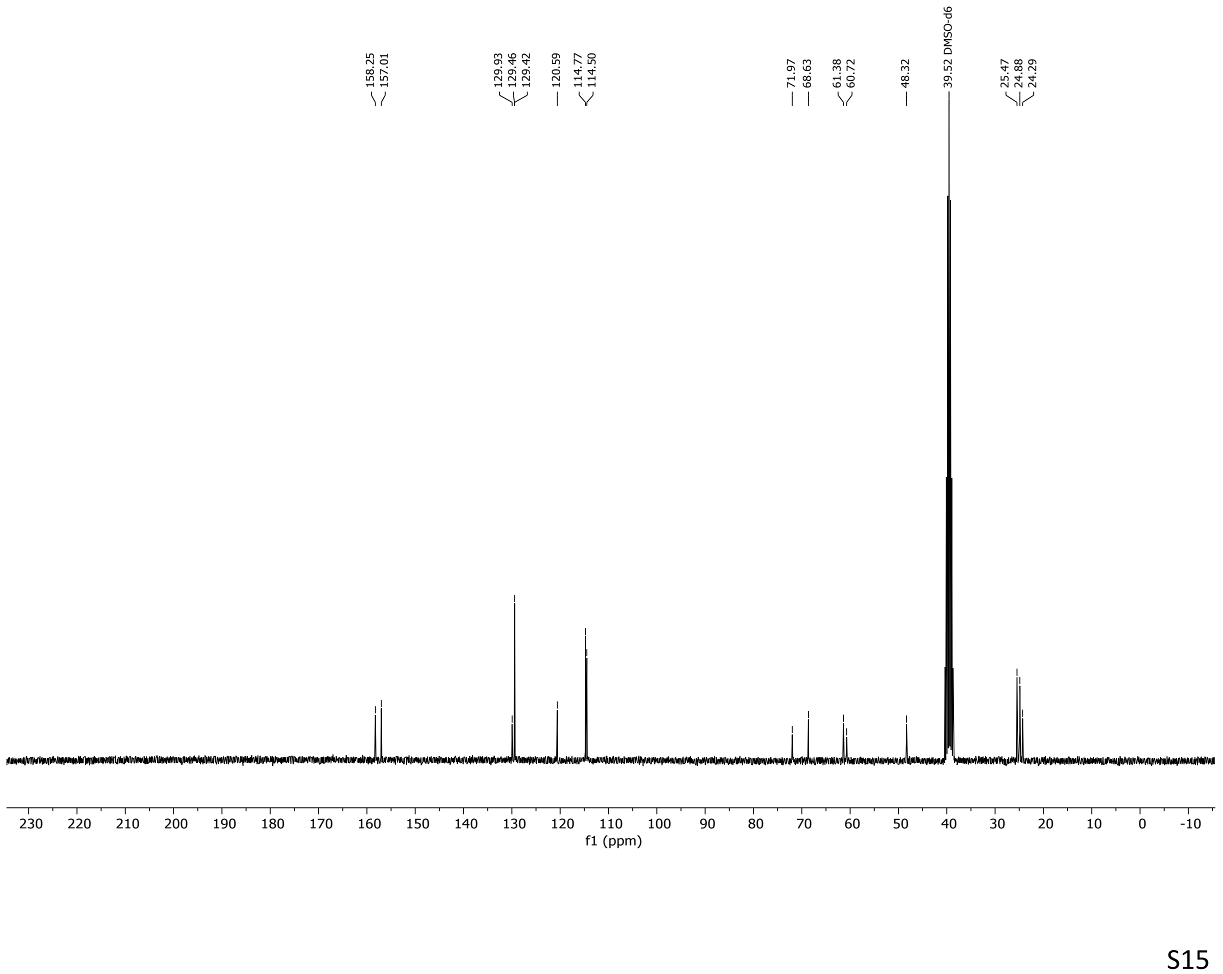

HPLC analysis of key final compounds

*(E)-N,N-dimethyl-N-(2-(4-styrylphenoxy)ethyl)cyclopentanaminium iodide (**7**).*

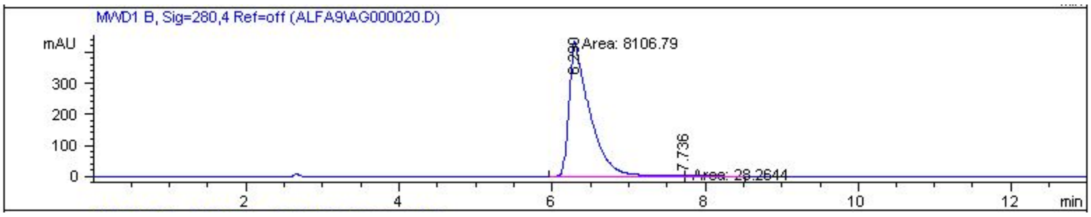

Signal 2: MWD1 B, Sig=280,4 Ref=off

Peak #	RetTime [min]	Type	Width [min]	Area [mAU*s]	Height [mAU]	Area %
1	6.290	MF	0.3096	8106.78760	436.34604	99.6526
2	7.736	FM	0.3965	28.26437	1.18819	0.3474
Totals :				8135.05197	437.53423	

*(E)-N,N-dimethyl-N-(3-(4-styrylphenoxy)propyl)cyclohexanaminium iodide (**14**).*

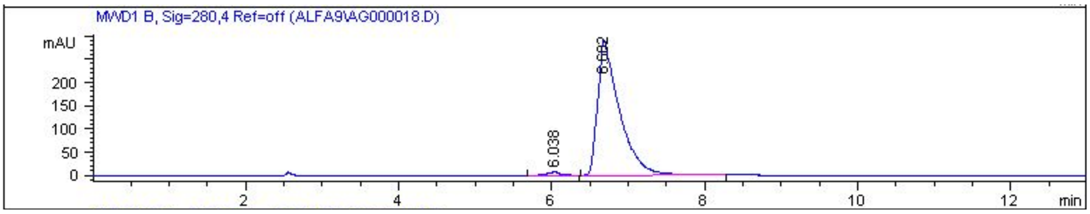

Signal 2: MWD1 B, Sig=280,4 Ref=off

Peak #	RetTime [min]	Type	Width [min]	Area [mAU*s]	Height [mAU]	Area %
1	6.038	PB	0.1648	80.54035	7.07369	1.3855
2	6.682	BB	0.2606	5732.67139	290.03561	98.6145
Totals :				5813.21174	297.10930	

*N-(2-(4-(benzyloxy)phenoxy)ethyl)-N,N-dimethylcyclohexanaminium iodide (**21**).*

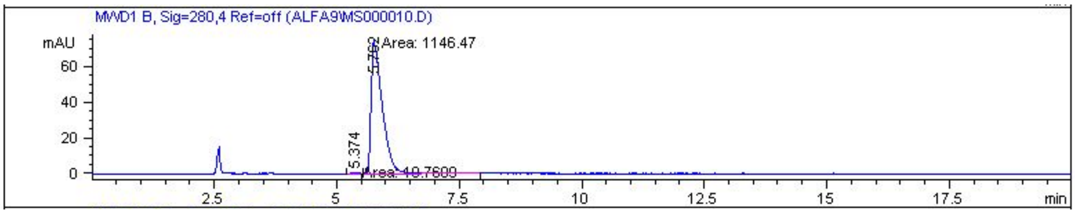

Signal 2: MWD1 B, Sig=280,4 Ref=off

Peak #	RetTime [min]	Type	Width [min]	Area [mAU*s]	Height [mAU]	Area %
1	5.374	MM	0.2527	10.76091	7.09594e-1	0.9299
2	5.762	FM	0.2552	1146.47412	74.88698	99.0701
Totals :				1157.23503	75.59657	

A Cpd 21, canonical site **B Cpd 22, canonical site**

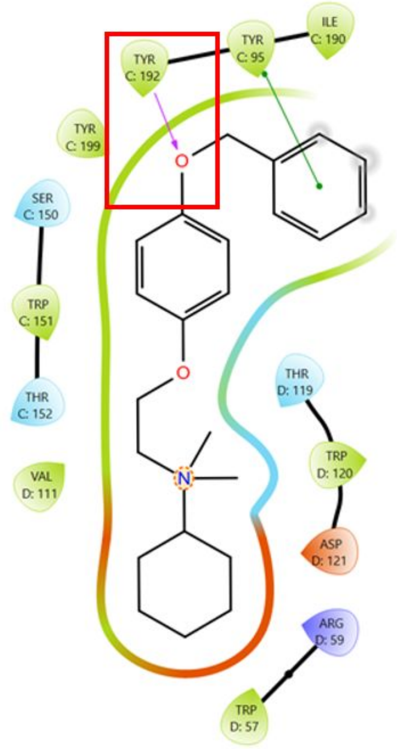

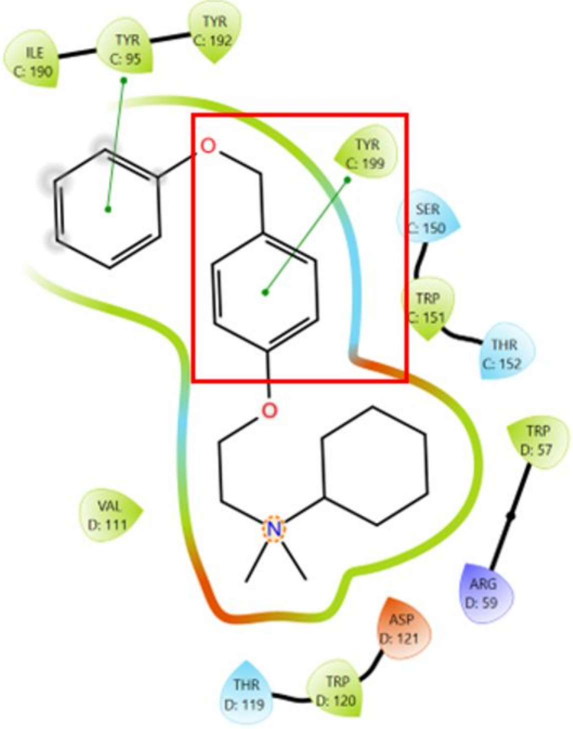

C Cpd 21, allosteric site **D Cpd 22, allosteric site**

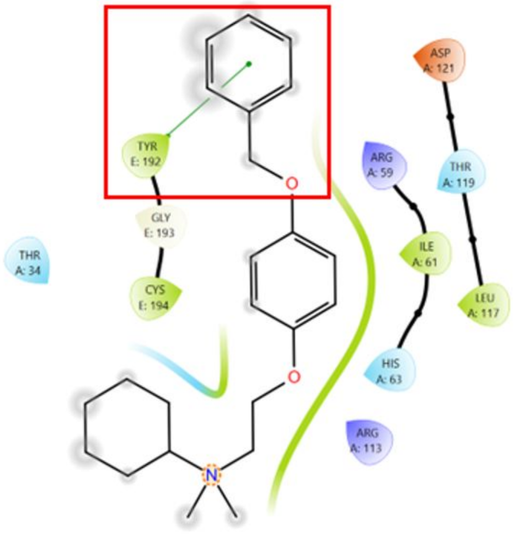

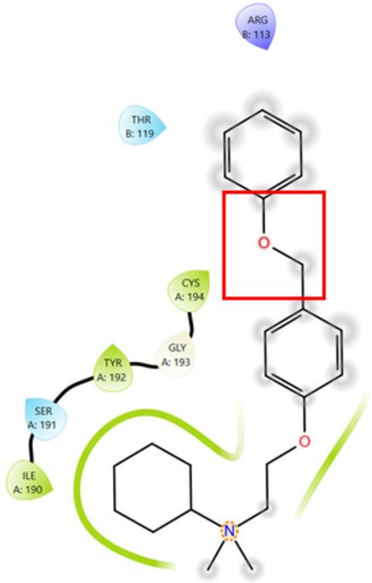

Figure S1. 2D ligand-receptor interaction diagrams for initial docking models, showing human α9 canonical agonist site residues interacting with **A)** compound **21**; **B)** compound **22**. Residues at the allosteric site just outside the tip of loop C (see **Figure 6D**) which bind with secondary ligands are shown for **C)** compound **21** and **D)** compound **22**. The benzyloxy oxygen (**21**) and phenoxymethyl oxygen (**22**) are circled, being the only structural difference between the two ligands. The purple arrow indicates hydrogen bond, while green lines terminated with small circles indicate π-π stacking interactions. Figures obtained using Schroedinger Maestro 2025.[1]

Table S1. Computed parameters values of physicochemical properties, lipophilicity, pharmacokinetics, drug-likeness and medicinal chemistry for compounds **2**, **21**, and **22** predicted using SwissADME web tool.[2]

Molecule	Compound **2**	Compound **21**	Compound **22**
Formula	$C_{24}H_{32}NO^+$	$C_{23}H_{32}NO_2^+$	$C_{23}H_{32}NO_2^+$
MW	350.52	354.51	354.51
#Heavy atoms	26	26	26
#Aromatic heavy atoms	12	12	12
Fraction Csp3	0.42	0.48	0.48
#Rotatable bonds	7	8	8
#H-bond acceptors	1	2	2
#H-bond donors	0	0	0
MR	111.83	107.44	107.44
TPSA	9.23	18.46	18.46
iLOGP	0.92	0.87	0.66
XLOGP3	6.03	5.22	5.22
WLOGP	5.43	4.90	4.90
MLOGP	0.84	0.10	0.10
Silicos-IT Log P	2.85	2.17	2.17
Consensus Log P	3.21	2.65	2.61
ESOL Log S	-5.69	-5.14	-5.14
ESOL Solubility (mg/ml)	7.13e-04	2.57e-03	2.57e-03
ESOL Solubility (mol/l)	2.03e-06	7.24e-06	7.24e-06
ESOL Class	Moderately soluble	Moderately soluble	Moderately soluble
Ali Log S	-6.00	-5.36	-5.36
Ali Solubility (mg/ml)	3.49e-04	1.56e-03	1.56e-03
Ali Solubility (mol/l)	9.95e-07	4.41e-06	4.41e-06
Ali Class	Poorly soluble	Moderately soluble	Moderately soluble
Silicos-IT LogSw	-7.78	-7.83	-7.83
Silicos-IT Solubility (mg/ml)	5.82e-06	5.21e-06	5.21e-06
Silicos-IT Solubility (mol/l)	1.66e-08	1.47e-08	1.47e-08
Silicos-IT class	Poorly soluble	Poorly soluble	Poorly soluble
GI absorption	High	High	High
BBB permeant	No	Yes	Yes
Pgp substrate	Yes	Yes	Yes
CYP1A2 inhibitor	Yes	Yes	Yes
CYP2C19 inhibitor	No	No	No
CYP2C9 inhibitor	No	No	No
CYP2D6 inhibitor	Yes	Yes	Yes
CYP3A4 inhibitor	Yes	Yes	Yes
log Kp (cm/s)	-4.16	-4.76	-4.76
Lipinski #violations	0	0	0
Ghose #violations	0	0	0
Veber #violations	0	0	0

Egan #violations	0	0	0
Muegge #violations	1	1	1
Bioavailability Score	0.55	0.55	0.55
PAINS #alerts	0	0	0
Brenk #alerts	2	1	1
Leadlikeness #violations	2	3	3
Synthetic Accessibility	2.81	2.69	2.69

References

1. Schrödinger Release 2025-3: BioLuminate, Schrödinger, LLC, New York, NY, 2025.

2. Daina, A.; Michielin, O.; Zoete, V; SwissADME: a free web tool to evaluate pharmacokinetics, drug-likeness and medicinal chemistry friendliness of small molecules. *Sci Rep.* **2017**, *7*, 42717.
